# Diethylene Glycol‐Linked Ring‐Fused Bis‐Azoles: Target‐Oriented Synthesis, In Silico Screening, and Antitumor Activity

**DOI:** 10.1002/cmdc.70504

**Published:** 2026-09-29

**Authors:** Sami A. Al‐Hussain, Abdulrahman S. Alharbi, Zeinab A. Muhammad, Alyaa S. Abdel Halim, Magdi E. A. Zaki, Mark C. Bagley, Refaie M. Kassab

**Affiliations:** ^1^ Department of Chemistry Faculty of Science Imam Mohammad Ibn Saud Islamic University (IMSIU) Riyadh KSA; ^2^ Department of Chemistry College of Science and Humanities‐Dawadmi Shaqra University Sajir Saudi Arabia; ^3^ Department of Pharmaceutical Chemistry Egyptian Drug Authority (EDA) Giza Egypt; ^4^ Department of Biochemistry Faculty of Science Ain Shams University Cairo Egypt; ^5^ Department of Chemistry University of Sussex Brighton East Sussex UK; ^6^ Department of Chemistry Faculty of Science Cairo University Giza Egypt

**Keywords:** antitumor agents, cytotoxicity, fused‐ring systems, heterocycles, VEGFR‐2/FGFR‐2 inhibitors

## Abstract

The target‐oriented synthesis of diethylene glycol‐linked bis‐imidazo‐ and bis‐thiazolo ring‐fused mono‐, bi‐, and tricyclic derivatives was accomplished using inexpensive reagents, bis‐phenacyl bromide derivative **4**, and thiosemicarbazone **6**. The diethylene glycol‐linked architectures included bis(thiazolo[3,2‐*a*]pyrimidinone) derivatives **8a,b**, bis(imidazo‐azole) derivatives **12‐14**, bis(imidazo‐thiazole) derivatives **17,18**, and bis‐thiazoles **20a‐g**. Cytotoxic evaluation against liver, lung, colon, and prostate human tumor cell lines found remarkable selectivity to hepatocellular carcinoma (HepG2) cells. The most potent bis‐imidazo‐ and bis‐thiazolo‐fused derivatives **8a,b**, **12** and **13** demonstrated cytotoxicity against HepG2 cells (IC_50_ 6.8–17.8 µM) with exceptional selectivity (SI 5.6–14.6) compared with cisplatin (SI 2.4). Further screening of their VEGFR‐2 TK and FGFR‐2 TK inhibition showed that **8a** had moderate activity, whereas phenyl‐substituted derivative **8b** displayed promising anti‐VEGFR‐2 and remarkable anti‐FGFR‐2 activities, with IC_50_ 0.19 ± 0.01 and 0.03 ± 0.01 µM, respectively, compared with the reference drugs sorafenib (IC_50_ 0.12 ± 0.01) and staurosporine (IC_50_ 0.03 ± 0.01 µM). The inhibition of VEGFR‐2 and FGFR‐2 TKs was supported by molecular docking studies, with compound **8b** showing binding affinity to the VEGFR‐2 and FGFR‐2 ATP binding pockets. Pharmacokinetics and ADMET drug‐likeness evaluation indicated that bis(thiazolo[3,2‐*a*]pyrimidinone) derivatives **8a** and **8b** have drug‐like properties.

## Graphical abstract

A series of diethylene glycol‐linked cyclic derivatives with diversity in the heterocyclic motif have been synthesized and screened for antitumor activity. Two bis‐thiazolo[3,2‐a]pyrimidinone derivatives displayed moderate cytotoxicity, high selectivity against hepatocellular carcinoma cells, promising VEGFR2, and remarkable FGFR2 inhibitory activities supported by molecular docking experiments.

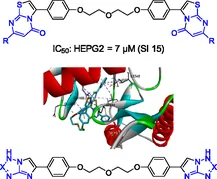

## 1 Introduction

With 10 million fatalities and close to 20 million new cases worldwide in 2022, cancer is considered one of the major threats to humankind, responsible for almost 1 in 6 deaths [1]. Every organ in the human body is susceptible to cancer, which can spread swiftly through either lymphatic or blood vessels to other bodily organs. The development of improved cancer treatments is a priority target for the clinic [2], where the location and stage of the disease impact upon the effectiveness of treatments. Besides radiation therapy and/or surgical removal, chemotherapy is one of the most widely used and effective therapeutic strategies [3]. Between 1950 and 2015, the US FDA registered 150 chemotherapeutic drugs [4], and almost 60% of their database comprises unique small‐molecule nitrogen‐based heterocycles [5]. For example, FDA approval of imidazole‐containing anticancer drugs included mercaptopurine and its pro‐drug azathioprine for the treatment of lymphoblastic leukemia [6, 7], dacarbazine for the treatment of malignant melanoma [8], and temozolomide for the treatment of newly diagnosed glioblastoma multiforme (Figure 1) [9]. Similarly, many thiazole‐based candidates have emerged as promising anticancer agents [10, 11], such as the topoisomerase II inhibitor vosaroxin [12], the pro‐apoptotic agent KUD773 for the treatment of human prostate tumors [13], and tiazofurin analogs as IMP dehydrogenase inhibitors [14]. More than 18 thiazole‐containing chemotherapeutic agents have been approved by the FDA for use in the clinic [15], including the anticancer agents dabrafenib, as an inhibitor of BRAF from the proto‐oncogene B‐RAF [16], and dasatinib, a tyrosine kinase inhibitor for the treatment of acute lymphoblastic leukemia and chronic myelogenous leukemia (Figure 2) [17]. Thus, evidentially, imidazole and thiazole scaffolds have demonstrable anticancer potential [18, 19].

**FIGURE 1:**
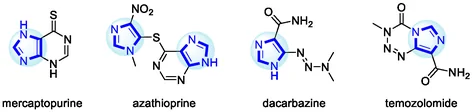
Examples of imidazole‐containing and clinically approved anticancer drugs.

**FIGURE 2:**
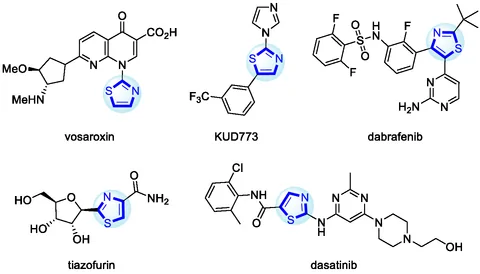
Examples of thiazole‐containing anticancer agents under clinical study or approved by the FDA.

Previous research has demonstrated that combining two or more heterocycles to create a “hybrid” molecule could ultimately result in a bis‐functionalized motif that is more effective than either of its original components [20, 21]. Numerous examples of combinatorial libraries of bis‐heterocyclic compounds where two distinct heterocyclic rings are joined by a linker have been assembled in the last 20 years [22]. However, bis‐heterocyclic templates in general, and bis‐imidazoles and bis‐thiazoles in particular, have not been investigated extensively, even though the majority of small‐molecule approved drugs historically and anticancer compounds specifically have been nitrogen‐containing heterocycles [23]. Recently, the anticancer properties of bis‐heterocyclic motifs has attracted significant interest [24], including those of ether‐linked symmetrical 1,3‐thiazine derivatives [25]. Pharmacological inhibitors of cyclin‐dependent kinase (CDK) have been evaluated extensively for therapeutic use against cancer and other diseases [26], and recently aryl hydrazinylthiazoles **I** and **II** have been shown to have remarkable antiproliferative effects and CDK2 inhibitory properties (IC_50_ 0.4 μM or 0.02–1.6 nM, respectively) [27, 28]. The aryl or coumarin surface in hydrazinylthiazole **I** or its coumarin congener **II** provided vital hydrophobic (HPO) binding connections for appropriate fitting to CDK2 HPO side chains, while the hydrazinylthiazole fragment provided hydrophilic (HPI) H‐bonds with the polar ATP‐binding site (Figure 3) [30].

**FIGURE 3:**
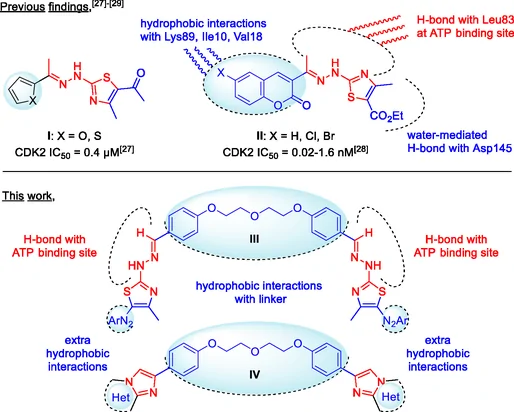
Remarkable CDK binding of bis‐heterocycles **I** and **II** and a generic structural‐based drug design (SBDD) hypothesis [29] for diethylene glycol‐linked bis‐heterocycles **III** and **IV**.

The synthesis and biological properties of nitrogen‐containing fused heterocycles have attracted increasing attention in recent years [31], employing nonconventional strategies [32] and exploring novel reactivity [33] to access new chemotypes in medicinal chemistry [34, 35, 36, 37, 38, 39, 40] and new FDA‐approved medicines containing *N*‐heterocycles [41]. Encouraged by the previously mentioned data and in extension of our well‐established interest in the properties of bis‐heterocyclic scaffolds in general [42], and imidazole [43] and thiazole frameworks in particular [44, 45, 46, 47, 48, 49], we now report, in the present study, the architectural design and synthesis of diethylene glycol‐linked bis‐imidazo‐fused and bis‐thiazolo‐fused mono‐, bi‐, and tricyclic templates as anticancer prototypes and describe their structure‐based drug design as potential inhibitors of vascular endothelial growth factor receptor‐2 (VEGFR‐2) and fibroblast growth factor receptor (FGFR‐2). Additionally, cytotoxic evaluation of these templates has been carried out and is discussed against four different human cancers derived from liver (HepG‐2), lung (A‐549), colorectal (HCT‐116), and prostate (PC‐3) cell lines, utilizing cisplatin as a reference standard. Finally, comprehensive molecular docking analyses of all novel bis‐heterocyclic scaffolds as potential anticancer prototypes are provided.

## 2 Results and Discussion

### 2.1 Chemistry

Since the diethylene glycol‐linked bis‐phenacyl bromide template **4** would represent the core building block for a synthetic strategy toward bis‐hydrazinyl thiazole derivatives **III** [50], its construction was investigated from readily available bis‐carbonyl scaffolds **3** [51, 52]. The diethylene glycol‐linked bis‐phenacyl bromide **4** should be accessible by acid‐promoted α‐bromination of bis‐acetophenone derivative **3b**. The latter was acquired via simple alkylation of *p*‐hydroxy acetophenone **1b** with the corresponding dihalo‐derivative **2** by a modified method of Katrizky [51]. Thereafter, upon boiling an acetonitrile solution of bis‐acetyl ketone derivative **3b**, *N*‐bromosuccinimide (NBS), and *p*‐toluenesulfonic acid (PTSA), the target bis(α‐bromoacetophenone) derivative **4** was obtained in 96% yield (Scheme 1).

**SCHEME 1:**
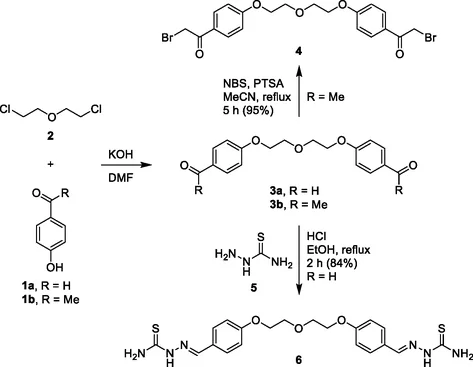
Synthesis of diethylene glycol‐linked scaffolds: bis‐phenacyl bromide derivative **4** and bis‐thiosemicarbazone derivative **6**.

Bis‐phenacyl bromide derivative **4** was heated at reflux with pyrimidinedione derivatives **7a** and **7b** in dioxane solution containing triethylamine (Et_3_N) to give bis‐thiazolopyrimidine derivatives **8a,b** (Scheme 2). Confirmation of the fused structure was established by spectral and other fundamental analyses. From the IR spectroscopic data, along with the disappearance of N–H and C = S bands, a strong carbonyl absorption band at 1658 cm^−1^ was observed. Furthermore, the loss of the N–H singlet and the appearance of the two expected singlets for the thiazole H‐2 and pyrimidine H‐6 at *δ* 7.22 and 8.55 ppm, respectively, were observed in the ^1^H NMR spectrum of **8a** and confirmed the thiazolo[3,2‐*a*]pyrimidin‐5‐one structure.

**SCHEME 2:**
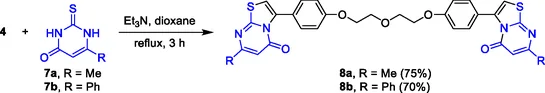
Synthesis of diethylene glycol‐linked bis‐thiazolopyrimidinone derivatives **8a,b**.

To further probe the versatility of the bis‐phenacyl bromide template **4** as a useful synthetic scaffold, it was reacted with three different heteroaromatic amines **9‐11** (Scheme 3) and two aminothiazole derivatives **15,16** (Scheme 4). Heating a solution of bis‐phenacyl bromide derivative **4** with *N*‐heterocyclic amines **9‐11** in dry ethanol gave the corresponding bis‐imidazo‐azole derivatives **12‐14** in good yield (Scheme 3) or reaction with 2‐aminothiazole (**15**) or its benzothiazole analog **16** under identical conditions gave imidazothiazoles **17** and **18**, respectively (Scheme 4).

**SCHEME 3:**
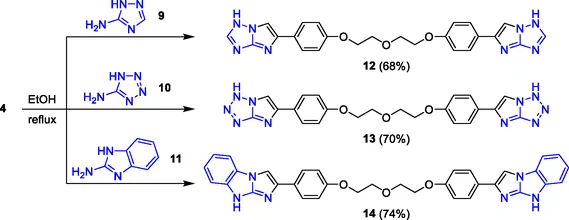
Synthesis of diethylene glycol‐linked bis‐imidazo‐azole derivatives **12‐14**.

**SCHEME 4:**
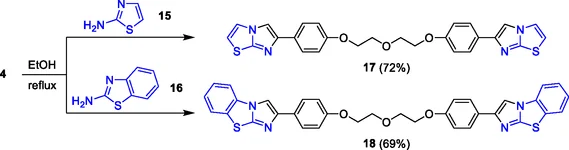
Synthesis of diethylene glycol‐linked bis‐imidazo‐thiazole derivatives **17** and **18**.

Our final synthetic goal was to exploit the versatility of bis‐thiosemicarbazone derivative **6** as a novel ether‐linked bis‐thiosemicabazone platform for bis‐thiazole synthesis [53, 54, 55]. Building block **6** was easily prepared by the acid‐catalyzed addition‐elimination reaction of bis‐aldehyde **3a** with two equivalents of thiosemicarbazide **5** (Scheme 1). The diversity of bis‐thiosemicarbazone derivative **6** was demonstrated by a one‐step reaction with a group of hydrazonoyl chlorides **19a‐g** according to our literature method [55]. Heating a solution of bis‐thiosemicarbazone **6**, a hydrazonoyl halide **19a‐g** [56, 57], and a catalytic quantity of triethylamine at reflux in dioxane gave the corresponding diethylene glycol‐linked bis‐thiazole **20a‐g** in good yield (Scheme 5).

**SCHEME 5:**
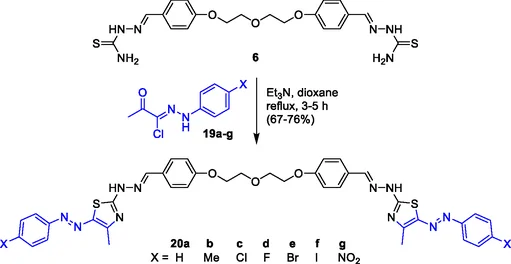
Synthesis of diethylene glycol‐linked bis‐thiazole derivatives **20a‐g**.

Various spectroscopic and analytical methods were used to thoroughly elucidate the structure of targets **20a‐g**. For example, in the spectroscopic analysis of bis‐thiazole **20a** ^1^H NMR spectroscopy revealed three singlets at *δ* 2.46, 7.15, and 8.22 ppm, corresponding to two homotopic methyl (Me) groups, hydrazone methines (CH = N), and NH groups, respectively. In addition to two distinct pairs of CH_2_ group resonances observed from the polyether linker at *δ* 3.83 and 4.20 ppm, the aromatic methine (ArH) resonances were observed at *δ* 7.36–7.89 ppm. Additionally, the infrared spectrum of bis‐thiazole **20a** showed the typical NH stretching absorption at 3433 cm^−1^, mass spectrometric analysis observed the molecular ion (*m/z* 744, M^•+^), and combustion analysis confirmed elemental compositions to give additional confirmation of identity and purity.

### 2.2 Biological Evaluation

#### 2.2.1 In Vitro Cytotoxicity

The newly synthesized diethylene glycol‐linked bis‐imidazo/thiazolo‐fused mono‐, bi‐, and tricyclic templates **8a, b**, **12**, **13**, **14**, **17**, and **18**, and the bis‐hydrazothiazole derivatives **20a‐g**, together with their respective bis‐phenacyl bromide **4** and bis‐carbazone precursors **6**, were all examined for their cytotoxic potential using four distinctive human tumor cell lines, explicitly, hepatocellular carcinoma (HepG‐2), lung carcinoma epithelial cells (A‐549), colorectal carcinoma cells (HCT‐116), and prostate tumor cells (PC‐3), measured against the potency of cisplatin as the reference standard by MTT (3‐(4,5‐dimethylthiazol‐2‐yl)‐2,5‐diphenyltetrazolium bromide) assay. The cytotoxicity data were collected as growth inhibitory concentration IC_50_ values in μM (Table 1). These bis‐scaffolds displayed varying levels of antiproliferative activity versus the examined cell lines, with more prominent cytotoxicity exhibited against HepG2 cells by compounds **8a**, **8b**, **12**, and **13**, which showed the lowest recorded IC_50_ values in the range 6.8–17.8 µM. It was observed that A‐549 cells were more sensitive to bis‐compounds **8a**, **8b**, and **12**, with IC_50_ values for these agents ranging from 21 to 33 µM, and this cytotoxicity profile was repeated in all cancer cell lines under study (Table 1). Although the synthesized bis‐imidazo‐fused heterocyclic derivatives were generally less potent than cisplatin, their biological activity should be interpreted in light of their distinct mechanism of action. Cisplatin exerts its cytotoxic effect primarily through DNA cross‐linking, leading to irreversible DNA damage and apoptosis. In contrast, the present compounds were designed as targeted kinase inhibitors, aiming to interfere with VEGFR‐2‐ and FGFR‐2‐mediated signaling pathways involved in tumor proliferation and angiogenesis. Consequently, direct comparison of IC_50_ values should consider these mechanistic differences.

**TABLE 1:** In vitro cytotoxic activity, IC_50_/μM, of diethylene glycol‐linked bis‐derivatives on the cell viability of HepG2, A‐549, HCT‐116, and PC‐3 cancer cell lines.^a^

Compound	HepG‐2	A‐549	HCT‐116	PC‐3
**4**	35.4 ± 3.6	47 ± 4.5	28.4 ± 3.6	55.5 ± 3.6
**6**	30.22 ± 3.60	38.8 ± 4.60	52.2 ± 3.60	62.21 ± 3.6
**8a**	6.87 ± 1.5	21.11 ± 1.6	18.20 ± 1.5	25.24 ± 1.5
**8b**	8.71 ± 3.7	24.30 ± 3.7	20.42 ± 3.7	28.32 ± 3.7
**12**	14.42 ± 2.3	33.20 ± 3.6	14.2 ± 2.3	20.42 ± 2.3
**13**	17.8 ± 2.80	42.10 ± 2.8	22.9 ± 2.3	26.3 ± 2.80
**14**	80.20 ± 2.90	58.20 ± 2.0	44.48 ± 2.9	38.1 ± 2.90
**17**	32.10 ± 5.3	72 ± 1.9	70 ± 1.9	72.1 ± 5.3
**18**	62.12 ± 1.9	56.71 ± 1.8	44.8 ± 1.9	34.8 ± 1.9
**20a**	85.20 ± 4.6	47.52 ± 2.1	34.18 ± 4.6	42.14 ± 4.6
**20b**	70.12 ± 8.52	63.08 ± 1.9	44.72 ± 8.52	56.3 ± 8.52
**20c**	27.20 ± 0.3	57.9 ± 2.1	87.44 ± 0.3	47.44 ± 0.3
**20d**	35.62 ± 2.4	34 ± 4.3	63.8 ± 2.4	34.9 ± 2.4
**20e**	74.35 ± 6.2	56.30 ± 7.3	54.23 ± 6.2	43.12 ± 6.2
**20f**	115.23 ± 0.8	66.13 ± 1.3	111.41 ± 0.8	69.20 ± 0.8
**20g**	66.24 ± 0.8	44.68 ± 1.3	62.31 ± 0.8	38.42 ± 0.8
Cisplatin	3.67 ± 0.15	7.53 ± 0.51	7.20 ± 0.15	17.6 ± 0.15

a IC_50_, the concentration of the compound (μM) producing 50% inhibition of cell growth after 24 h of compound exposure. Results are represented as the mean standard deviation (SD) of three independent experiments. Highly active, 1 μM < IC_50_ < 10 μM; moderately active, 10 μM < IC_50_ < 100 μM; inactive, IC_50_ > 100 μM.

Structure‐activity relationship indicates that the substitution pattern on the bis‐imidazo‐fused scaffold plays a critical role in determining cytotoxic activity. Compounds **8a** and **8b** were the most active members of the series against HepG2 cells (IC_50_ = 6.87 and 8.71 μM, respectively), suggesting that their structural features provide favorable steric and electronic characteristics for efficient interaction with the kinase binding pocket. Molecular docking studies could further support this observation (see later) by demonstrating stable binding interactions with the catalytic residues of VEGFR‐2 and FGFR‐2. Conversely, derivatives containing bulkier or less optimally substituted groups showed markedly reduced cytotoxic activity, indicating that excessive steric hindrance or unfavorable electronic effects may weaken target binding affinity. These findings establish an initial SAR that may serve as a valuable framework for future structural optimization aimed at improving potency while maintaining target specificity.

Screening the selectivity profile of the most potent members of the newly synthesized bis‐imidazo/thiazolo‐fused mono‐, bi‐, and tricyclic templates, derivatives **8a,b**, **12**, and **13** not only outperformed the rest of their bis‐heterocyclic counterparts but also outclassed the reference standard itself as shown (Table 2). For A‐549, HCT‐116, or PC‐3 malignant cell lines, the bis‐imidazo/thiazolo‐fused derivatives **8a**, **8b**, **12**, and **13** displayed selectivity indices (SI) between 2.4 and 7.0, compared with selectivity indices of 0.5–1.2 for cisplatin itself. However, for the hepatocellular cancer cell line HepG‐2, the bis‐imidazo/thiazolo‐fused derivatives **8a**, **8b**, **12**, and **13** showed outstanding selectivity against HepG‐2 carcinoma over the normal cell line WI‐38, with selectivity indices (SI) between 5.6–14.6, compared with a selectivity index of 2.4 for cisplatin itself (see Table 2 and Figure 4).

**TABLE 2:** Selectivity indices (SI) of the most potent bis‐compounds against HepG‐2, A‐549, HCT‐116, and PC‐3 cancer cell lines as compared with normal cells (WI‐38).^a^

Comp.	WI‐38 cytotoxicity IC_50_, μM	HepG2 SI^a^	A549 SI^a^	HCT116 SI^a^	PC3 SI^a^
**8a**	>100	14.6	4.7	5.5	4.0
**8b**	>100	11.5	4.1	4.9	3.5
**12**	>100	6.9	3.0	7.0	4.9
**13**	>100	5.6	2.4	4.4	3.8
**14**	70 ± 3	0.9	1.2	1.6	1.8
**17**	>100	3.1	1.4	1.4	1.4
**18**	>100	1.6	1.8	2.2	2.9
Cisplatin	9.0	2.4	1.2	1.2	0.5

a Selectivity index (SI) values were determined by dividing the IC_50_ of each normal cell line (IC_50_‐normal) by the IC_50_ of the cancerous cell line (IC_50_‐cancerous) after three independent experiments.

**FIGURE 4:**
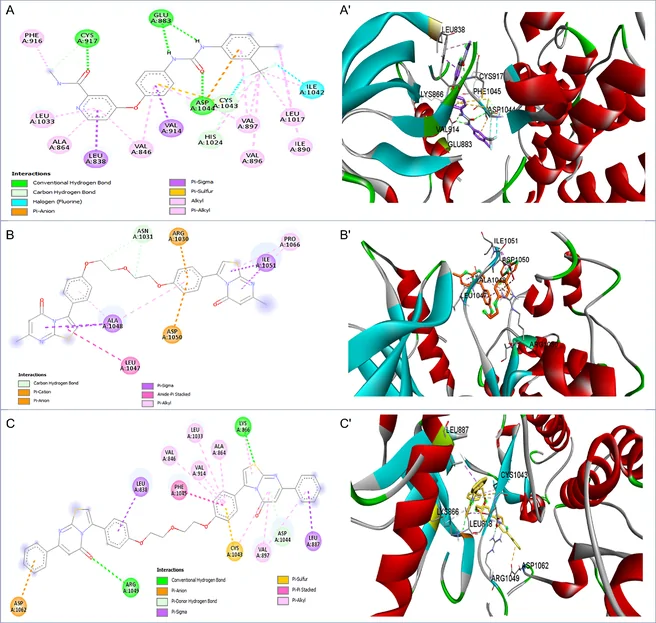
(A–C) Two‐dimensional (2D) interactions of co‐crystallized (A) sorafenib, (B) compound **8a**, and (C) compound **8b**, with the VEGFR‐2 active site and their corresponding 3D representations (A′)–(C′).

#### 2.2.2 Structure Activity Relationships (SAR) Assessment

Correlation between the observed antiproliferative cytotoxic activity of the newly synthesized ethylene glycol‐linked bis‐imidazo‐fused and bis‐thiazolo‐fused templates and their respective structural topographies concluded there was no unambiguous evidence for any general SAR pattern. However, multiple specific structure–activity relationships (SARs) could be postulated. First, despite the high potency of both bis‐thiazolopyrimidines derivatives **8a,b**, substituting the bulkier phenyl group (Ph) in **8b** with the smaller methyl group (Me) in **8a** gave a small enhancement in cytotoxic activity (IC_50_) from 8.71 to 6.87 μM. Additionally, both bis‐imidazo‐azole derivatives **12** and **13** showed good antiproliferative potency with IC_50_ of 14.42 and 17.8 μM, respectively, possibly due to additional binding interactions provided by H‐bond formation via the free N–H group. On the other hand, extending conjugation in the bis‐imidazo‐thiazole derivative **17** with an additional benzene ring, as shown in its counterpart **18**, seemed to dramatically reduce the antiproliferative activity (IC_50_) from 30.22 to 62.12 μM. A similar argument could be made to explain the poor cytotoxic performance of the benzo‐fused bis‐imidazo‐imidazole counterpart **14** (IC_50_ 80.20 μM). Unexpectedly, from all the synthesized bis‐arylazothiazole derivatives **20a‐g**, only chloro‐substituted and fluoro‐substituted compounds **20c** and **20d** showed enhanced cytotoxic potencies with IC_50_ of 27.20 and 35.62 μM, respectively, which could be ascribed to an ideal charge‐over‐radius proportion of the two halogen substituents.

Based on the cytotoxicity screening, compounds **8a** and **8b** were selected for further biological evaluation because they exhibited the lowest IC_50_ values against HepG2 cells among all synthesized derivatives, with IC_50_ values of 6.87 ± 1.50 and 8.71 ± 3.70 μM, respectively. These findings identified them as the lead compounds of the series and justified further investigation of their underlying mechanism of action. VEGFR‐2 and FGFR‐2 were selected as molecular targets because both receptor tyrosine kinases are critically involved in hepatocellular carcinoma progression [58, 59], through regulation of angiogenesis, cell proliferation, survival, and tumor progression. Therefore, compounds **8a** and **8b** were evaluated for VEGFR‐2 and FGFR‐2 kinase inhibition, followed by molecular docking studies, to determine whether their potent antiproliferative activity could be associated with inhibition of these therapeutically relevant targets.

#### 2.2.3 VEGFR‐2 TK Inhibitory Activity

Vascular endothelial growth factor receptor 2 tyrosine kinase (VEGFR‐2 TK) is a tyrosine kinase subordinate present in vascular endothelial tissues that responds to VEGF transmission at receptor ATP binding sites, developing vascular proliferation [60]. Therefore, discovering novel anticancer agents that can inhibit VEGFR‐2 has been recognized as an important therapeutic objective [61]. Consequently, our attention shifted toward screening the two most potent ethylene glycol‐linked bis‐thiazolopyrimidinone derivatives **8a** and **8b** as potential VEGFR‐2 inhibitors utilizing sorafenib as a standard drug. The IC_50_ values for the anti‐VEGFR‐2 potency of the most active derivatives are presented (Table 3 and Figure 5). While the bis‐thiazolopyrimidine derivative **8a** had a moderate anti‐VEGFR‐2 potency with IC_50_ value of 0.605 ± 0.015 µM, its phenyl‐substituted counterpart **8b** presented a comparable VEGFR‐2 inhibitory potency with IC_50_ estimated at 0.189 ± 0.006 µM, measured against sorafenib (IC_50_ 0.123 ± 0.002 µM).

**TABLE 3:** IC_50_ values/µM of VEGFR2 and FGFR2 kinase inhibition activities.^a^

Compound	VEGFR‐2 IC_50_, µM^a^	FGFR‐2 IC_50_, µM^a^
**8a**	0.605 ± 0.015	0.268 ± 0.011
**8b**	0.189 ± 0.006	0.089 ± 0.004
Sorafenib	0.123 ± 0.002	—
Staurosporine	—	0.032 ± 0.001

a IC_50_, the concentration of the compound/μM producing 50% inhibition of VEGFR‐2 and FGFR‐2b activity. Results are represented as the mean standard deviation (SD) of three independent experiments.

**FIGURE 5:**
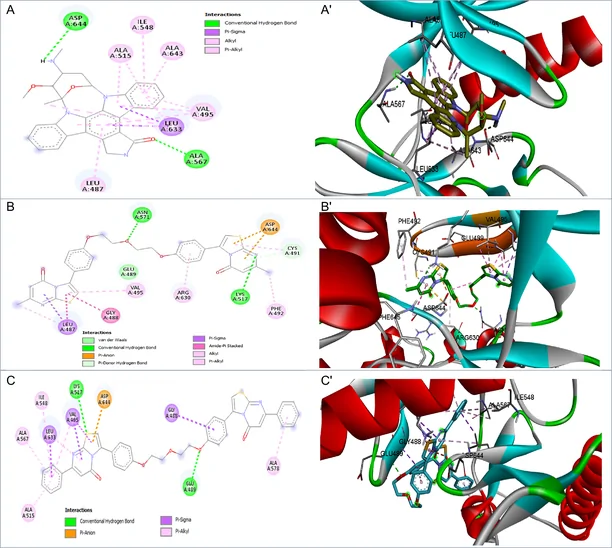
(A–C) Two‐dimensional (2D) interactions of co‐crystallized (A) staurosporine, (B) compound **8a**, and (C) compound **8b**, with the FGFR‐2 active site and their corresponding 3D representations (A′)–(C′).

#### 2.2.4 FGFR‐2 TK Inhibitory Activity

Fibroblast growth factor receptor 2 tyrosine kinase (FGFR‐2 TK) is a tyrosine kinase subordinate present in normal and malignant cells and is correlated with several biological activities that involve angiogenic and mitogenic proliferation. Therefore, discovering novel anticancer agents that can inhibit FGFR‐2 has been recognized as an excellent therapeutic target [62]. Consequently, our attention was shifted toward screening the two most potent diethylene glycol‐linked bis‐thiazolopyrimidinone derivatives **8a** and **8b** as plausible FGFR‐2 inhibitors. Staurosporine was employed as the positive control in the FGFR‐2 kinase inhibition assay because it is a potent ATP‐competitive broad‐spectrum protein kinase inhibitor that has been widely used as a reference compound in kinase profiling studies. Although staurosporine is not selective for FGFR‐2, it exhibits potent inhibitory activity against numerous receptor protein kinases. Therefore, its use in the present study was intended to validate the assay performance and provide a standardized positive control for comparing the inhibitory activities of the synthesized compounds rather than to represent a selective FGFR‐2 inhibitor. Also, staurosporine was chosen as a high‐affinity pan‐kinase ligand useful in docking studies [63]. The IC_50_ estimates for the anti‐FGFR‐2 potency of these bis‐thiazolopyrimidines derivatives are shown (Table 3). While thiazolopyrimidine derivative **8a** had moderate anti‐FGFR‐2 activity with IC_50_ estimate of 0.268 ± 0.011 µM, its phenyl‐substituted counterpart **8b** displayed a comparable FGFR‐2 inhibitory activity (IC_50_) estimated at 0.032 ± 0.001 µM, measured against staurosporine (IC_50_ 0.032 ± 0.001 µM).

### 2.3 In Silico Evaluation

#### 2.3.1 Molecular Docking with the VEGFR‐2 Active Site

To gain supplementary insights into the anticancer efficacy and inhibitory effects on VEGFR‐2 and FGFR‐2 tyrosine kinases, the diethylene glycol‐linked bis‐thiazolopyrimidine derivatives **8a** and **8b** were further evaluated by a molecular docking analysis to assess their bonding patterns with the functional reactive pockets of the prospective target and further analyzed by molecular computations to assess their capability to inhibit the function of VEGFR‐2 kinase. Docking experiments were performed on the ATP active pocket of the 3D crystal structure (PDB ID: 2OH4) of the VEGFR‐2 kinase using AutoDock 4.2 software. The aim was to examine the binding interactions and explore the potential binding modes of these new bis‐thiazolopyrimidine derivatives. Additionally, the co‐crystallized ligand was subjected to docking versus VEGFR‐2 kinase to predict the possible binding modes, affinity, and preferred orientation of each docking posture, with sorafenib being used as a standard drug. The production of vascular endothelial growth factor (VEGF) by cancer cells and its interaction with its receptor (VEGFR‐2) is crucial for the genesis and advancement of cancer [64]. Thus, targeting the VEGFR‐2 signaling pathway is a promising therapeutic strategy to diminish tumor growth by suppressing angiogenesis [65].

Results of the docking analysis with the reactive pocket of VEGFR‐2, including the binding energy and binding interactions of the most potent bis‐hydrazothiazoles **8a** and **8b**, are shown (Table 4 and Figure 4), with sorafenib as a standard reference drug. Its binding energy was estimated at −12.75 kcal mol^−1^ and involves H‐bonding interaction with the amino acid CYS917 at 2.00 Å as shown (Figure 4A). Furthermore, two binding interactions were provided by amino acid residue ASP1044 forming a H‐bond, a pi–anion bond, and a pi–sulfur bond. Additionally, a pair of H‐bonds and a pair of pi‐sigma interactions were established through the amino acid sequence GLU883 and the amino acids LEU838 and VAL914, respectively. On the other hand, and despite its high binding energy (−11.06 kcal mol^−1^), the reduced inhibitory effect of derivative **8a** could be attributed to the lack of H‐bonding connections with the crucial amino acid residue in the ATP binding pocket of VEGFR‐2 (Figure 4B). These docking data suggest that the bis‐thiazolopyrimidine derivative **8a** and the reference drug sorafenib would interact with VEGFR‐2 through two different mechanisms. The promising diethylene glycol‐linked bis‐thiazolopyrimidine derivative **8b** displayed superior binding with a higher binding score (−13.29 kcal mol^−1^) measured against the co‐crystalized ligand sorafenib (−12.75 kcal mol^−1^), outperforming the latter via a pair of H‐bonds with crucial amino acid terminals LYS866 and ARG1049 (Figure 4C).

**TABLE 4:** Molecular docking data of the most potent bis‐thiazolopyrimidine derivatives 8a and 8b with the VEGFR 2 kinase and FGFR 2 TK active sites compared with the co‐crystalized inhibitors sorafenib and staurosporine, respectively.

Compound	VEGFR‐2			FGFR‐2		
	Binding energy	Interactions	Distance, Å	Binding energy	Interactions	Distance, Å
Reference drug^a^	−12.75	CYS917 H‐bond ASP1044 H‐bond GLU883 H‐bond GLU883 H‐bond LEU838 π‐sigma VAL914 π‐sigma ASP1044 π‐sulfur ASP1044 π‐anion	2.00 2.96 2.16 2.00 3.96 3.63 5.41 4.73	−10.40	ASP644 H‐bond ALA567 H‐bond LEU633 π‐sigma LEU633 π‐sigma LEU633 π‐alkyl LEU487 π‐alkyl ALA515 π‐alkyl ALA515 π‐alkyl ILE548 π‐alkyl ALA643 alkyl	2.20 2.23 3.36 3.19 5.00 5.14 4.46 5.24 4.86 5.16
**8a**	−11.06	LEU1047 Amide π‐stacked ALA1048 π‐sigma ALA1048 π‐sigma ARG1030 π‐cation ASP1050 π‐anion ILE1051 π‐sigma ILE1051 π‐sigma	5.46 3.57 3.46 3.16 4.55 3.74 3.79	−10.63	ASN571 H‐bond LYS517 H‐bond ASP664 π‐anion ASP664 π‐anion LEU487 π‐sigma LEU487 Amide π‐stacked GLY488 Amide π‐stacked VAL495 alkyl ARG630 alkyl PHE492 alkyl	1.79 2.20 3.76 3.54 3.81 3.55 4.07 4.76 5.14 4.70
**8b**	−13.29	LYS866 H‐bond LEU887 π‐sigma ASP1062 π‐anion ARG1049 H‐bond LEU838 π‐sigma CYS1043 π‐sulfur	2.46 3.79 3.96 2.91 3.85 5.44	−9.74	GLU489 H‐bond LYS517 H‐bond ASP644 π‐anion GLY488 π‐sigma LEU633 π‐sigma LEU633 π‐alkyl VAL495 π‐sigma VAL495 π‐sigma ALA567 π‐alkyl ALA515 π‐alkyl ALA578 π‐alkyl	2.04 2.29 3.35 3.85 3.40 5.49 3.62 3.58 5.06 3.58 4.45

a Ligands used as reference drugs were sorefinib for binding to VEGFR2 and staurosporine for binding to FGFR2.

#### 2.3.2 Molecular Docking with the FGFR‐2 Active Site

Fibroblast growth factor receptor 2 (FGFR2) is part of the receptor tyrosine kinase group, essential for numerous cellular activities including proliferation, differentiation, and tissue development [66]. Additionally, random signaling of FGFR2 has been associated with multiple health problems, including cancer [67]. Therefore, clinical trials are seeking the advancement of powerful, selective FGFR2 kinase inhibitors that can target the FGFR2 signaling pathway, offering an efficient therapeutic strategy to diminish tumor growth and suppress cell proliferations in FGFR2‐implicated cancers [68].

The most potent members of the new diethylene glycol‐linked bis‐heterocycles **8a** and **8b** were further evaluated by molecular computational estimation to assess their capability to inhibit the activity of FGFR‐2 (fibroblast growth factor receptor 2) kinase. The docking simulations were performed on the ATP active pocket of the 3D crystal structure (PDB ID: 7KIA) of the FGFR‐2 kinase using AutoDock 4.2 software to examine the binding interactions and explore the potential binding modes of **8a** and **8b**. Additionally, the co‐crystallized ligand staurosporine was subjected to docking against FGFR‐2 kinase as a reference to predict and contrast the possible binding modes, affinity, and preferred orientation of each docking posture (Table 4 and Figure 5). The co‐crystallized reference kinase inhibitor staurosporine and its interactions with the reactive centers of FGFR2 were examined: its binding energy was estimated at −10.40 kcal mol^−1^, involving a pair of crucial H‐bonds with the important amino acids ASP644 and ALA567 (see Figure 5A). Furthermore, five π‐alkyl binding interactions were provided by amino acid residues LEU487, ALA515, ILE548, and ALA643. On the other hand, and despite its relatively high binding energy (−10.63 kcal mol^−1^) resulting from multiple binding interactions (as shown in Table 4), the reduced inhibitory activity of bis‐thiazolopyrimidine derivative **8a** (Figure 5B) could be attributed to the lack of H‐bonding connections with the crucial amino acid residues ASP644 and ALA567, within the ATP binding site of FGFR2 and the co‐crystallized ligand staurosporine (Figure 5A).

Alternatively, and in addition to multiple π‐sigma, π‐alkyl, and π‐anion binding interactions, the bis‐thiazolopyrimidine derivative **8b** established two key H‐bonding interactions with amino acid terminals ASP644 and ALA567 (Table 4 and Figure 5C). As a result, bis‐thiazolopyrimidine derivative **8b** binds into the FGFR2 active site with a binding tally of −9.74 kcal mol^−1^, measured against a binding tally of −10.40 kcal mol^−1^ for the co‐crystallized ligand staurosporine. The addition of the 7,7′‐phenyl substituents into the bis(thiazolopyrimidinone) core of **8b** as compared with **8a** was found to introduce additional π‐alkyl and π‐sigma interactions with Ala, Leu, and Ile residues in a hydrophobic pocket of the active site (Figure 5C′), thereby accounting for its dramatically enhanced activity. The binding energy scores of bis‐thiazolopyrimidinone derivative **8b** are in good accord with the experimental inhibitory activity data.

**FIGURE 6:**
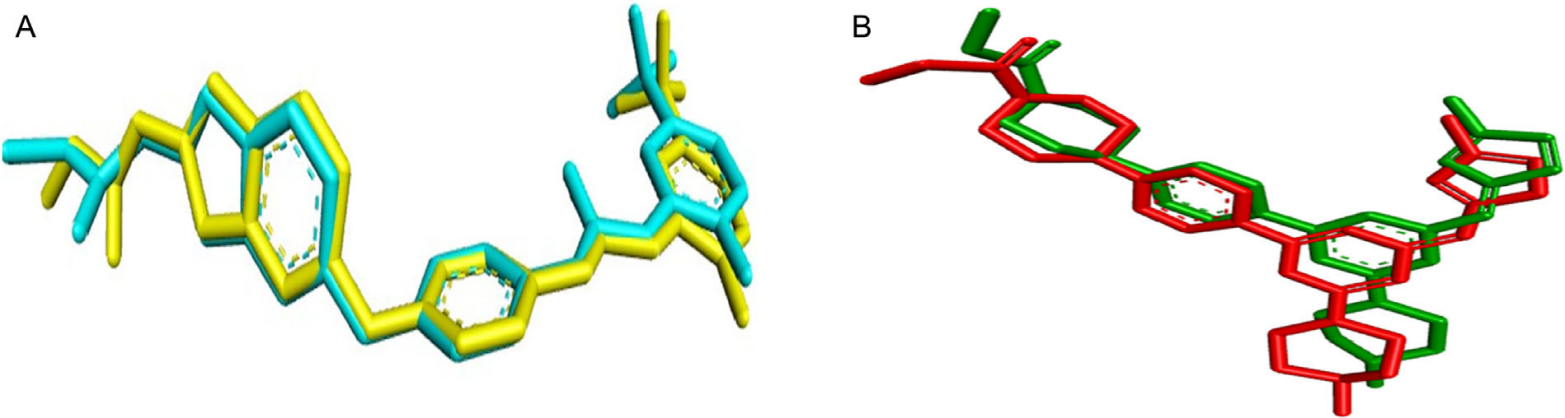
Validation of the molecular docking protocol: (A) the ligand re‐docked pose and crystal structure pose within the VEGFR‐2 active site shown in cyan and gold, respectively, and (B) the ligand re‐docked pose and crystal structure pose within the FGFR‐2 active site shown in red and green, respectively.

#### 2.3.3 Verification of the Molecular Docking Methods

The legitimacy of the molecular computational method employed in this study was validated by executing a re‐docking approach of the ligand inside the X‐ray crystal structure, as illustrated (Figure 6). Afterward, the root mean square deviation (RMSD) was estimated by comparing the redocked position with the facade identified in the crystal structure. Successful docking is achieved when the RMSD is smaller than a given threshold of 2.0 Å after docking. Therefore, a ligand RMSD in the redocked crystal structure poses of 0.54 and 1.2 Å for VEGFR‐2 and FGFR‐2, respectively, confirmed the success of the adopted molecular docking protocol.

#### 2.3.4 In Silico Pharmacokinetics and Drug‐Likeness Assessment

Pharmacokinetic drug–drug exchanges between two distinct drugs occur when a drug alters the absorption, distribution, metabolism, excretion, and/or toxicity (ADMET) of another drug. Potency, toxicity, and possible pharmacokinetic interactions are all important determinants in developing effective medications [69]. The optimal binding characteristics between a given drug and the corresponding medicinal targets are crucial factors in drug discovery. Additionally, drug delivery to the intended site at a sufficient concentration level to initiate the desired physiological response is another important requirement to fulfill before its therapeutic application. Therefore, the recognition of ADMET attributes has become an integral part of the preliminary phases of any drug discovery program. This has markedly diminished the quantity of drugs that failed clinical trials owing to improper ADMET capabilities. Lipinski's rule is utilized to evaluate drug‐likeness and ascertain if a candidate, possessing certain pharmacological or biological activity, holds the requisite chemical and physical qualities to qualify as a prospective orally administered medicine [70]. According to Lipinski's rule of five, for a medicine to be effective when taken orally, it should not violate more than one of the subsequent requirements: a molecular weight (MW) ≤ 500, H‐bond donors ≤ 5, H‐bond acceptors ≤ 10, and a calculated octanol‐water partition coefficient (log P o/w) ≤ 5.

From the compiled data (Table 5), the most potent diethylene glycol‐linked bis‐thiazolopyrimidine derivatives **8a** and **8b** had zero H‐bond donors (HBD = 0), which are fewer than sorafenib and staurosporine (HBD = 3 and 2, respectively). Additionally, the H‐bond acceptors in both bis‐thiazolopyrimidine derivatives **8a** and **8b** are identical to those of sorafenib (HBA = 7). Furthermore, and aside from a single violation (MW > 500), both bis‐thiazolopyrimidine derivatives **8a** and **8b** complied with Lipinski's rule of five. Moreover, a topological polar surface area (TPSA) of 152.91 Å^2^, falling below the threshold of 160 Å^2^, serves as a strong indicator of the potential bioavailability of these bis‐thiazolopyrimidine derivatives. Additionally, lipophilicity (log P(o/w)) of 5.25 and 6.15 for bis‐thiazolopyrimidine derivatives **8a** and **8b**, respectively, indicates good lipophilicity. Finally, a drug‐likeness score of 0.55 for both **8a** and **8b** is identical to that of the reference drugs sorafenib and staurosporine. In conclusion, both bis‐thiazolopyrimidine derivatives **8a** and **8b** adhere to Lipinski's rule of five, indicating their drug‐likeness characteristics and consequently, their high potential for oral bioactivity. ADMET computational prediction analyzes the pharmacokinetic characteristics of a drug prototype and helps foresee its pharmacological fate post‐administration. The two most potent bis‐thiazolopyrimidine derivatives 8a and 8b were further subjected to pharmacokinetics prediction using pkCSM computational application as a predictive model of central ADMET properties for drug discovery and development. Full ADMET data for compounds **8a** and **8b** are provided (Table 6).

**TABLE 5:** Pharmacokinetic properties of the most potent derivatives as computed by SwissADME.

Property	8a	8b	Sorafenib	Staurosporine
MW	586.68	710.82	464.82	466.53
H‐bond donors (HBD)	0	0	3	2
H‐bond acceptors (HBA)	7	7	7	4
TPSA	152.91	152.91	92.35	69.45
Lipophilicity (Log P(o/w))	5.25	6.15	3.42	3.19
Water solubility (Log S)	MS	PS	MS	MS
Lipinski (no. of violations)	Yes (1); MW > 500	Yes (1); MW > 500	Yes (0)	Yes (0)
Bioavailability score	0.55	0.55	0.55	0.55

Abbreviations: MS, moderately soluble; MW, molecular weight (g/mol); PS, poorly soluble; TPSA, topological polar surface area (Å^2^).

**TABLE 6:** ADMET predictions using pkCSM.

Property	8a	8b	Sorafenib	Staurosporine
Absorption				
Water solubility	−3.518	−2.955	−5.089	−3.685
Caco2 permeability	0.238	0.259	0.959	1.073
Intestinal absorption (human)	100	100	85.272	96.58
Skin permeability	−2.735	−2.735	−2.746	−2.735
P‐glycoprotein substrate	No	No	Yes	Yes
P‐glycoprotein I inhibitor	Yes	Yes	Yes	Yes
P‐glycoprotein II inhibitor	Yes	Yes	Yes	Yes
Distribution				
VDss (human)	0.968	0.027	−0.218	−0.677
Fraction unbound (human)	0.312	0.361	0	0.286
BBB permeability	−1.907	−2.023	−1.642	−0.161
CNS permeability	−3.365	−2.934	−2.031	−1.973
Metabolism				
CYP2D6 substrate	No	No	No	No
CYP3A4 substrate	Yes	Yes	Yes	Yes
CYP1A2 inhibitor	No	No	No	No
CYP2C19 inhibitor	Yes	No	Yes	No
CYP2C9 inhibitor	Yes	Yes	Yes	No
CYP2D6 inhibitor	No	No	No	No
CYP3A4 inhibitor	Yes	No	No	Yes
Excretion				
Total clearance	−0.036	0.373	−0.212	1.253
Renal OCT2 substrate	No	No	No	Yes
Toxicity				
AMES toxicity	No	No	No	Yes
Max. tolerated dose (human)	0.581	0.444	0.507	0.255
hERG I inhibitor	No	No	No	No
hERG II inhibitor	Yes	Yes	Yes	Yes
Oral rat acute toxicity (LD50)	2.624	2.508	2.131	2.422
Oral rat chronic toxicity (LOAEL)	0.361	−1.219	1.306	1.293
Hepatotoxicity	Yes	No	Yes	No
Skin sensitization	No	No	No	No

Abbreviations: BBB, blood‐brain barrier; Caco2, Caco2 cell permeability; CYP450, cytochrome P450 enzymes; DILI, drug‐induced liver injury; hERG, the human Ether‐à‐go‐go‐related gene; HIA, human intestinal absorption; P‐gp, P‐glycoprotein.

#### 2.3.5 Absorption Analysis

Relevant pharmacokinetic parameters were calculated based on water solubility, Caco‐2 cell permeability, human intestinal absorption (HIA), and the ability to serve as a substrate and inhibitor of P‐glycoprotein. Based on the data (Table 6), bis‐thiazolopyrimidine derivatives **8a** and **8b** exhibited moderate water solubility and favorable distribution. Furthermore, they may demonstrate a favorable degree of in vivo absorption across the small intestinal barrier, which is evaluated by the Caco‐2 permeability assay. Additionally, derivatives **8a** and **8b** exhibited a high intestinal absorption (HIA) value of 100%, surpassing that of sorafenib and staurosporine HIA values of 85.3% and 96.6%, respectively. Therefore, the bis‐thiazolopyrimidine derivatives **8a** and **8b** are expected to exhibit enhanced absorption from the gastrointestinal system following oral dosing. Even though these compounds could not perform as P‐glycoprotein substrates, they could still be regarded as good inhibitors for P‐glycoprotein I/II.

#### 2.3.6 Distribution Analysis

The distributional properties of the most potent diethylene glycol‐linked bis‐thiazolopyrimidine derivatives **8a** and **8b** are summarized (Table 6) and include volume of distribution (VD), plasma protein binding (PPB), blood‐brain barrier diffusion (BBB), and central nervous system (CNS) penetrability. The data presented indicates that **8a** and **8b** possess satisfactory VD, PPB, and CNS characteristics. Computational analysis studies also revealed a low blood‐brain barrier penetration (BBB) of −1.907 and −2.023 for compounds **8a** and **8b**, compared with the reference standards sorafenib and staurosporine, −1.642 and −0.161, respectively (Table 6).

#### 2.3.7 Metabolism and Excretion Analysis

The bis‐thiazolopyrimidine derivatives **8a** and **8b** exhibited substrate activity toward the CYP3A4 enzyme but not towards the CYP2D6 enzyme. Furthermore, derivative **8a** was predicted to inhibit the CYP2C19, CYP2C9, and CYP3A4 enzymes but not the CYP1A2 and CYP2D6 enzymes. In contrast, bis‐thiazolopyrimidine derivative **8b** was predicted to inhibit only the CYP2C9 enzyme while exhibiting a satisfactory clearance rate (Table 6).

#### 2.3.8 Toxicity Analysis

The AMES test identifies the potential mutagenic nature of a drug prototype. Based on the data, bis‐thiazolopyrimidine derivatives **8a** and **8b** exhibited negative AMES test results, and hence they lack mutagenicity potential. Additionally, both derivatives showed no inhibitory activity against hERG I (Table 6). In conclusion, comprehensive computational analysis indicates that the two bis‐thiazolopyrimidine derivatives **8a** and **8b** exhibit anticipated ADME values resembling those of standard drugs sorafenib and staurosporine.

## 3 Experimental Section

Commercially available reagents were used without further purification and solvents were dried by standard procedures. Unless otherwise stated, reactions were performed under an atmosphere of air. Analytical thin layer chromatography (tlc) was carried out using aluminum‐backed plates coated with Merck Kieselgel 60 GF254 that were visualized under UV light (at 254 and/or 360 nm); fully characterized compounds were chromatographically homogeneous by tlc analysis. Melting points were determined on an Electrothermal IA 9000 series digital melting point apparatus and are uncorrected. Infrared spectra were recorded in the range 4000–400 cm^−1^ on a JASCO FT‐IR‐4100 spectrophotometer using an ATR probe or using KBr disks or as a nujol mull for solid samples and thin films between NaCl plates for liquid samples and are reported in cm^−1^. NMR spectra were recorded using a Varian Gemini 300 NMR spectrometer at 300 MHz or a Bruker Avance III 400 MHz for ^1^H spectra and 100 or 75 MHz for ^13^C spectra; *J* values were recorded in Hz and multiplicities were expressed by the usual conventions. Low‐resolution mass spectra were determined using a Shimadzu GCMS‐QP 1000EX single quadrupole gas chromatograph‐mass spectrometer using electron ionization. Elemental analyses were determined using a Elementar Vario‐LIII C‐H‐N‐S analyzer under high‐temperature conditions (>1200 °C), calibrated using acetanilide (CHN) or sulfanilimide (CHNS) standards, on samples dried for at least 48 h in vacuo using an oil pump and stored in a vacuum desiccator prior to analysis; no matrix‐matched standards were employed.

### 3.1 Synthesis of Bis‐Phenacyl Bromide 4

NBS (3.56 g, 20 mmol) was added slowly in portions to a stirred mixture of bis‐acetophenone derivative **3b** (3.42 g, 10 mmol) and *p*‐TsOH (3.8 g, 20 mmol) in acetonitrile (50 mL). The reaction mixture was heated under reflux with continuous mixing for 3 h. Afterward, the reaction mixture was allowed to cool to ambient temperature. The solvent was removed under vacuum, and the remaining material was dissolved in dichloromethane (50 mL), washed with water (2 × 20 mL), and dried with anhydrous MgSO_4_. Upon removal of CH_2_Cl_2_, the resultant solid was purified by recrystallization from benzene to give the expected bis(α‐bromoketone) derivative **4** as fine off‐white crystals, 75% yield; m.p. 125–126 °C (benzene); ^1^H NMR (300 MHz, [D_6_]DMSO): *δ* = 8.02 (d, ^3^ *J* = 9.0 Hz, 4H; ArH), 7.02 (d, ^3^ *J* = 9.0 Hz, 4H; ArH), 5.22 (s, 4H; CH_2_Br), 4.19 (t, *J* = 4.5 Hz, 4H; CH_2_OAr), 3.82 (t, *J* = 4.5 Hz, 4H; CH_2_OCH_2_) ppm; IR: *ν*_max_ = 3073, 3034 (C–H aromatic), 1662 (C = O), 1292, 1257, 1219 cm^−1^; MS: *m/z* (%) = 500 (9) [*M* ^•+^], 482 (24), 454 (23), 410 (12), 376 (20), 319 (9), 257 (16), 240 (16), 193 (20), 138 (19), 105 (43), 97 (100), 82 (44), 71 (48), 66 (69); elemental analysis calcd (%) for C_20_H_20_Br_2_O_5_: C 48.03, H 4.03; found: C 48.21, H 3.87.

### 3.2 Synthesis of Bis‐Thiosemicarbazone Derivative 6

Concentrated HCl (0.30 mL) was added to a solution of bis‐aldehyde **3a** (3.14 g, 0.01 mol) and thiosemicarbazide **5** (1.82 g, 0.02 mol) in EtOH (30 mL) and the mixture was heated at reflux for 3 h. The formed yellow solid was filtered and recrystallized from AcOH to give the *title compound* as fine off‐white crystals, 84% yield; m.p. 225–226 °C; ^1^H NMR (300 MHz, [D_6_]DMSO): *δ* = 11.26 (bs, 2H; NH_2_), 8.13 (bs, 2H; NH_2_), 7.99 (s, 2H; CH = N), 7.84 (bs, 2H; NH_2_), 7.70 (d, ^3^ *J* = 9.0 Hz, 4H; ArH), 6.95 (d, ^3^ *J* = 9.0 Hz, 4H; ArH), 4.15 (t, *J* = 4.2 Hz, 4H; CH_2_OAr), 3.82 (t, *J* = 4.2 Hz, 4H; CH_2_OCH_2_) ppm; IR: *ν*_max_ = 3471, 3356 (NH_2_), 3155 (NH), 3008, 2931 (CH aromatic), 1242 (C = S), 1165, 1103 cm^−1^; MS: *m/z* (%) = 460 (7) [*M* ^•+^], 449 (20), 427 (15), 404 (17), 375 (13), 341 (100), 327 (55), 295 (32), 278 (19), 155 (20), 107 (22); elemental analysis calcd (%) for C_20_H_24_N_6_O_3_S_2_: C 52.16, H 5.25, N 18.25; found: C 52.29, H 5.16, N 18.17%.

### 3.3 General Procedure for the Synthesis of Bis‐Thiazolopyrimidine Derivatives 8a,b

Bis‐phenacyl bromide **4** (500 mg, 1 mmol) and Et_3_N (0.7 mL) were added to a swirled, preheated solution of pyrimidinethione derivative **7a** or **7b** (2 mmol) in dioxane (30 mL). The resulting mixture was heated under reflux for 6 h and monitored by TLC. Upon cooling, the precipitated material was separated by gravity filtration and washed with aqueous ethanol to dissolve any triethylamine hydrochloride crystals, air‐dried, and eventually purified by recrystallization from AcOH to give the corresponding bis‐thiazolopyrimidine derivative **8**.

### 3.4 3,3′‐(((Oxybis(ethane‐2,1‐Diyl))bis(oxy))bis(4,1‐Phenylene))bis(7‐Methyl‐5*H*‐Thiazolo[3,2‐*a*]pyrimidin‐5‐One) (8a)

Using 284 mg of **7a**, bis‐thiazolopyrimidine derivative **8a** was obtained as a yellow solid, 75% yield; m.p. 205–206 °C; ^1^H NMR (300 MHz, [D_6_]DMSO): *δ* = 8.54 (s, 2H; 6,6′‐H), 7.97–7.52 (m, 8H; ArH), 7.22 (s, 2H; 2,2′‐H), 4.12 (t, *J* = 4.5 Hz, 4H; CH_2_OAr), 3.82 (t, *J* = 4.5 Hz, 4H; CH_2_OCH_2_), 2.24 (s, 6H; 2Me) ppm; IR: *ν*_max_ = 2931, 2746 (C–H aromatic), 1658 (C = O), 1172, 1033 cm^−1^; MS: *m/z* (%) = 586 (19) [*M* ^•+^], 570 (14), 533 (12), 521 (18), 449 (31), 420 (18), 301 (88), 268 (65), 238 (28), 209 (68), 168 (46), 149 (63), 141 (32), 104 (42), 77 (56), 40 (100); elemental analysis calcd (%) for C_30_H_26_N_4_O_5_S_2_: C 61.42, H 4.47, N 9.55; found: C 61.58, H 4.35, N 9.43%.

### 3.5 3,3′‐(((Oxybis(ethane‐2,1‐Diyl))bis(oxy))bis(4,1‐Phenylene))bis(7‐Phenyl‐5*H*‐Thiazolo[3,2‐*a*]pyrimidin‐5‐One) (8b)

Using 408 mg of **7b**, bis‐thiazolopyrimidine derivative **8b** was obtained as a yellow solid, 70% yield; m.p. 195–196 °C; ^1^H NMR (300 MHz, [D_6_]DMSO): *δ* = 8.01 (s, 2H; 6,6′‐H), 7.75 (s, 2H; 2,2′‐H), 7.58–6.98 (18H; ArH), 4.19 (t, *J* = 4.5 Hz, 4H; CH_2_OAr), 3.84 (t, *J* = 4.5 Hz, 4H; CH_2_OCH_2_) ppm; ^13^C NMR (75 MHz, [D_6_]DMSO): *δ* = 162.3, 134.3, 133.5, 132.4, 129.4, 128.3, 125.9, 124.3, 122.7, 122.2, 118.1, 115.7, 114.8, 114.1, 70.6, 68.4 ppm; IR: *ν*_max_ = 3062, 2939 (C–H aromatic), 1689 (C = O), 1172, 1041 cm^−1^; MS: *m/z* (%) = 710 (20) [*M* ^•+^], 643 (53), 583 (40), 500 (64), 414 (49), 326 (56), 316 (50), 309 (100), 295 (73), 265 (42), 252 (55), 190 (33), 130 (42); elemental analysis calcd (%) for C_40_H_30_N_4_O_5_S_2_: C 67.59, H 4.25, N 7.88; found: C 67.68, H 4.12, N 7.71%.

### 3.6 General Procedure for the Reaction of Bis‐Phenacyl Bromide 4 with Heteroaromatic Amines

A solution of bis‐phenacyl bromide 4 (500 mg, 1 mmol) and the corresponding heteroaromatic amine **9‐11**, **15**, or **16** (2 mmol) in ethanol (20 mL) was heated at reflux for 7–10 h and monitored by TLC. The reaction mixture was cooled to ambient temperature, and the resulting precipitate was filtered, washed with water (2 × 30 mL), air‐dried, and purified by recrystallization from EtOH to give the desired product **12‐14**, **17**, or **18**.

### 3.7 5,5′‐(((Oxybis(ethane‐2,1‐Diyl))bis(oxy))bis(4,1‐Phenylene))bis(1*H*‐Imidazo[1,2‐*b*][1,2,4] Triazole) (12)

Using 168 mg of amine **9**, bis‐imidazotriazole derivative **12** was obtained as an off‐white solid, 68% yield; m.p. 180–181 °C; ^1^H NMR (300 MHz, [D_6_]DMSO): *δ* = 8.69 (s, 2H; 2NH), 8.47 (s, 2H; triazolyl 2‐H), 8.31 (s, 2H; imidazolyl 6,6′‐H), 8.03–7.03 (m, 8H; ArH), 4.26 (t, *J* = 4.5 Hz, 4H; CH_2_OAr), 3.86 (t, *J* = 4.5 Hz, 4H; CH_2_OCH_2_) ppm; IR: *ν*_max_ = 3441 (NH), 3055, 2931 (C–H aromatic), 1118, 1041 cm^−1^; MS: *m/z* (%) = 470 (27) [*M* ^•+^], 437 (86), 422 (25), 410 (30), 407 (37), 390 (56), 352 (55), 332 (39), 293 (33), 217 (49), 176 (57), 168 (64), 151 (69), 122 (100), 81 (86); elemental analysis calcd (%) for C_24_H_22_N_8_O_3_: C 61.27, H 4.71, N 23.82; found: C 61.43, H 4.58, N 23.69%.

### 3.8 5,5′‐(((Oxybis(ethane‐2,1‐Diyl))bis(oxy))bis(4,1‐Phenylene))bis(1*H*‐Imidazo[1,2‐*d*]tetrazole) (13)

Using 170 mg of amine **10**, bis‐imidazotetrazole derivative **13** was obtained as an off‐white solid, 70% yield; m.p. 169–170 °C; ^1^H NMR (300 MHz, [D_6_]DMSO): *δ* = 8.03 (d, ^3^ *J* = 9.0 Hz, 4H; ArH), 7.11 (bs, 2H; 2NH), 7.04 (d, ^3^ *J* = 9.0 Hz, 4H; ArH), 5.64 (s, 2H; 6,6′‐H), 4.22 (t, *J* = 4.5 Hz, 4H; CH_2_OAr), 3.82 (t, *J* = 4.5 Hz, 4H; CH_2_OCH_2_) ppm; IR: *ν*_max_ = 3610 (NH), 3050, 2930 (C–H aromatic), 1111, 1049 cm^−1^; MS: *m/z* (%) = 472 (33) [*M* ^•+^], 446 (49), 434 (44), 410 (33), 365 (44), 315 (23), 298 (70), 294 (49), 284 (61), 274 (86), 264 (100), 253 (67), 224 (50), 177 (54), 156 (69), 92 (45), 68 (66); elemental analysis calcd (%) for C_22_H_20_N_10_O_3_: C 55.93, H 4.27, N 29.65; found: C 56.11, H 4.14, N 29.52%.

### 3.9 2,2′‐(((Oxybis(ethane‐2,1‐Diyl))bis(oxy))bis(4,1‐Phenylene))bis(9*H*‐Benzo[*d*]imidazo[1,2‐*a*]imidazole) (14)

Using 266 mg of amine **11**, bis‐imidazoimidazole derivative **14** was obtained as a yellow solid, 74% yield; m.p. 175–176 °C; ^1^H NMR (300 MHz, [D_6_]DMSO): *δ* = 8.91 (s, 2H; 2NH), 8.06–7.16 (16H, ArH), 5.88 (s, 2H; 3,3′‐H), 4.27 (t, *J* = 4.5 Hz, 4H; CH_2_OAr), 3.86 (t, *J* = 4.5 Hz, 4H; CH_2_OCH_2_) ppm; ^13^C NMR (75 MHz, [D_6_]DMSO): *δ* = 145.3, 142.0, 141.2, 139.7, 137.4, 136.8, 133.5, 132.7, 130.4, 129.1, 122.8, 121.5, 120.3, 70.4, 68.9 ppm; IR: *ν*_max_ = 3518 (NH), 3055, 2931 (C–H aromatic), 1126, 1049 cm^−1^; MS: *m/z* (%) = 568 (16) [*M* ^•+^], 565 (24), 548 (27), 500 (39), 445 (29), 438 (38), 412 (37), 361 (42), 318 (27), 301 (100), 241 (23), 185 (35), 165 (35), 99 (31), 75 (48); elemental analysis calcd (%) for C_34_H_28_N_6_O_3_: C 71.82, H 4.96, N 14.78; found: C 71.98, H 4.85, N 14.66%.

### 3.10 6,6′‐(((Oxybis(ethane‐2,1‐Diyl))bis(oxy))bis(4,1‐Phenylene))bis(imidazo[2,1‐*b*]thiazole) (17)

Using 200 mg of amine **15**, bis‐imidazothiazole derivative **17** was obtained as an off‐white solid, 72% yield; m.p. 211–212 °C; ^1^H NMR (300 MHz, [D_6_]DMSO): *δ* = 8.42−7.02 (12H; ArH), 4.71 (s, 2H; 5,5′‐H), 4.20 (t, *J* = 4.5 Hz, 4H; CH_2_OAr), 3.83 (t, *J* = 4.5 Hz, 4H; CH_2_OCH_2_) ppm; IR: *ν*_max_ = 2940, 2885 (C–H), 1257, 1180, 1126, 1049 cm^−1^; MS: *m/z* (%) = 502 (19) [*M* ^•+^], 499 (13), 488 (27), 470 (33), 363 (36), 347 (35), 255 (18), 208 (14), 131 (12), 121 (100), 66 (32), 64 (54); elemental analysis calcd (%) for C_26_H_22_N_4_O_3_S_2_: C 62.14, H 4.40, N 11.14; found: C 62.25, H 4.32, N 11.04%.

### 3.11 2,2′‐(((Oxybis(ethane‐2,1‐Diyl))bis(oxy))bis(4,1‐Phenylene))bis(benzo[*d*]imidazo[2,1‐*b*]thiazole) (18)

Using 300 mg of amine **16**, bis(benzimidazothiazole) derivative **18** was obtained as a brown solid, 69% yield; m.p. 220–221 °C; ^1^H NMR (300 MHz, [D_6_]DMSO): *δ* = 8.38−7.04 (16H; ArH), 6.03 (s, 2H; 3,3′‐H), 4.14 (t, *J* = 4.5 Hz, 4H; CH_2_OAr), 3.83 (t, *J* = 4.5 Hz, 4H; CH_2_OCH_2_) ppm; ^13^C NMR (75 MHz, [D_6_]DMSO): *δ* = 141.2, 136.7, 135.4, 134.2, 133.8, 133.0, 131.4, 130.1, 128.0, 126.7, 125.7, 124.6, 122.2, 69.4, 67.5 ppm; IR: *ν*_max_ = 2924, 2889 (C–H aromatic), 1110, 1041 cm^−1^; MS: *m/z* (%) = 602 (12) [*M* ^•+^], 596 (17), 515 (17), 416 (30), 402 (41), 360 (17), 338 (25), 277 (66), 188 (40), 108 (47), 70 (100); elemental analysis calcd (%) for C_34_H_26_N_4_O_3_S_2_: C 67.75, H 4.35, N 9.30; found: C 67.87, H 4.22, N 9.12%.

### 3.12 General Procedure for the Preparation of Bis(dihydrazothiazole) Derivatives 20a‐g [54]

Et_3_N (0.7 mL) was added to an agitated solution of bis‐thiosemicarbazone derivative **6** (460 mg, 1 mmol) and the corresponding hydrazonoyl chloride derivative **19a‐g** [55, 56] (2 mmol) in dioxane (30 mL). The mixture was heated at reflux for 5 h and then allowed to cool. The precipitated triethylamine hydrochloride (Et_3_N.HCl) was removed by filtration, and the organic solvent was evaporated under vacuum. The remaining solid was filtered, washed with water (2 × 30 mL), air‐dried, and recrystallized from dioxane to give the corresponding bis(dihydrazothiazole) derivative **20a‐g**.

### 3.13 2,2′‐(((((Oxybis(ethane‐2,1‐Diyl))bis(oxy))bis(4,1‐Phenylene))bis(methanylylidene))bis(hydrazin‐1‐Yl‐2‐Ylidene))bis(4‐Methyl‐5‐(phenyldiazenyl)thiazole) (20a)

Using 393 mg of hydrazonoyl halide **19a**, the *title compound* was obtained as a dark red solid, 70% yield; m.p. 140–141 °C; ^1^H NMR (300 MHz, [D_6_]DMSO): *δ* = 8.22 (s, 2H; NH), 7.15 (s, 2H; CH = N), 7.89–7.36 (18H; ArH), 4.20 (t, *J* = 4.5 Hz, 4H; CH_2_OAr), 3.83 (t, *J* = 4.5 Hz, 4H; CH_2_OCH_2_), 2.46 (s, 6H; 2Me) ppm; IR: *ν*_max_ = 3433 (NH), 3055, 2877 (C–H aromatic), 1242, 1165 cm^−1^; MS: *m/z* (%) = 744 (12) [*M* ^•+^], 709 (13), 611 (8), 591 (5), 505 (6), 434 (6), 338 (10), 301 (9), 292 (9), 279 (9), 238 (15), 199 (32), 183 (24), 155 (24), 141 (19), 122 (100), 108 (37), 60 (72); elemental analysis calcd (%) for C_38_H_36_N_10_O_3_S_2_: C 61.27, H 4.87, N 18.80; found: C 61.41, H 4.75, N 18.64%.

### 3.14 2,2′‐(((((Oxybis(ethane‐2,1‐Diyl))bis(oxy))bis(4,1‐Phenylene))bis(methanylylidene))bis(hydrazin‐1‐Yl‐2‐Ylidene))bis(4‐Methyl‐5‐(*p*‐Tolyldiazenyl)thiazole) (20b)

Using 421 mg of hydrazonoyl halide **19b**, the *title compound* was obtained as a dark red solid, 75% yield; m.p. 151–152 °C; ^1^H NMR (300 MHz, [D_6_]DMSO): *δ* = 9.84 (s, 2H; NH), 8.51 (s, 2H; CH = N), 7.79–7.05 (16H; ArH), 4.19 (t, *J* = 4.5 Hz, 4H; CH_2_OAr), 3.83 (t, *J* = 4.5 Hz, 4H; CH_2_OCH_2_), 2.76 (s, 6H; 4,4′‐Me), 2.34 (s, 6H; 2Me) ppm; ^13^C NMR (75 MHz, [D_6_]DMSO): *δ* = 141.5, 137.8, 133.0, 130.2, 128.3, 125.4, 124.7, 124.0, 122.8, 120.4, 118.3, 117.2, 70.0, 69.1, 19.2 ppm; IR: *ν*_max_ = 3441 (NH), 3050, 2916 (C–H aromatic), 1249, 1165 cm^−1^; MS: *m/z* (%) = 772 (77) [*M* ^•+^], 733 (28), 684 (38), 645 (41), 617 (48), 578 (66), 544 (54), 432 (100), 410 (76), 314 (56), 291 (66), 279 (66), 257 (65), 219 (64), 111 (57), 99 (63); elemental analysis calcd (%) for C_40_H_40_N_10_O_3_S_2_: C 62.16, H 5.23, N 18.12; found: C 62.35, H 5.10, N 18.00%.

### 3.15 2,2′‐(((((Oxybis(ethane‐2,1‐Diyl))bis(oxy))bis(4,1‐Phenylene))bis(methanylylidene))bis(hydrazin‐1‐Yl‐2‐Ylidene))bis(5‐((4‐Chlorophenyl)diazenyl)‐4‐Methylthiazole) (20c)

Using 462 mg of hydrazonoyl halide **19c**, the *title compound* was obtained as a red solid, 73% yield; m.p. 158–159 °C; ^1^H NMR (300 MHz, [D_6_]DMSO): *δ* = 9.86 (s, 2H; NH), 8.54 (s, 2H; CH = N), 7.76–7.02 (16H; ArH), 4.19 (t, *J* = 4.5 Hz, 4H; CH_2_OAr), 3.84 (t, *J* = 4.5 Hz, 4H; CH_2_OCH_2_), 2.76 (s, 6H; 4,4′‐Me) ppm; IR: *ν*_max_ = 3441 (NH), 3045, 2924 (C–H aromatic), 1242, 1165 cm^−1^; MS: *m/z* (%) = 813 (45) [*M* ^•+^], 802 (77), 755 (33), 714 (55), 692 (52), 644 (100), 611 (44), 543 (60), 480 (32), 433 (63), 404 (66), 392 (57), 294 (61), 223 (82), 178 (81), 145 (48), 118 (60), 98 (54), 65 (51), 55 (46); elemental analysis calcd (%) for C_38_H_34_Cl_2_N_10_O_3_S_2_: C 56.09, H 4.21, N 17.21; found: C 56.21, H 4.05, N 17.08%.

### 3.16 2,2′‐(((((Oxybis(ethane‐2,1‐Diyl))bis(oxy))bis(4,1‐Phenylene))bis(methanylylidene))bis(hydrazin‐1‐Yl‐2‐Ylidene))bis(5‐((4‐Fluorophenyl)diazenyl)‐4‐Methylthiazole) (20d)

Using 429 mg of hydrazonoyl halide **19d**, the *title compound* was obtained as a red solid, 69% yield; m.p. 162–164 °C; ^1^H NMR (300 MHz, [D_6_]DMSO): *δ* = 9.84 (s, 2H; NH), 8.52 (s, 2H; CH = N), 7.78–7.02 (16H; ArH), 4.19 (t, *J* = 4.5 Hz, 4H; CH_2_OAr), 3.84 (t, *J* = 4.5 Hz, 4H; CH_2_OCH_2_), 2.76 (s, 6H; 4,4′‐Me) ppm; ^13^C NMR (75 MHz, [D_6_]DMSO): *δ* = 137.6, 136.5, 134.8, 131.2, 128.6, 127.2, 125.6, 124.8, 122.7, 121.7, 120.2, 118.2, 70.8, 69.5, 19.8 ppm; IR: *ν*_max_ = 3433 (NH), 3065, 2924 (C–H aromatic), 1242, 1168 cm^−1^; MS: *m/z* (%) = 780 (37) [*M* ^•+^], 744 (10), 699 (11), 653 (10), 648 (12), 566 (11), 488 (12), 396 (13), 346 (12), 295 (100), 210 (18), 173 (14), 77 (27), 62 (35); elemental analysis calcd (%) for C_38_H_34_F_2_N_10_O_3_S_2_: C 58.45, H 4.39, N 17.94; found: C 58.61, H 4.22, N 17.81%.

### 3.17 2,2′‐(((((Oxybis(ethane‐2,1‐Diyl))bis(oxy))bis(4,1‐Phenylene))bis(methanylylidene))bis(hydrazin‐1‐Yl‐2‐Ylidene))bis(5‐((4‐Bromophenyl)diazenyl)‐4‐Methylthiazole) (20e)

Using 551 mg of hydrazonoyl halide **19e**, the *title compound* was obtained as a dark red solid, 70% yield; m.p. 154–155 °C; ^1^H NMR (300 MHz, [D_6_]DMSO): *δ* = 9.84 (s, 2H; NH), 8.56 (s, 2H; CH = N), 7.82–7.04 (16H; ArH), 4.19 (t, *J* = 4.5 Hz, 4H; CH_2_OAr), 3.84 (t, *J* = 4.5 Hz, 4H; CH_2_OCH_2_), 2.76 (s, 6H; 4,4′‐Me) ppm; IR: *ν*_max_ = 3441 (NH), 3050, 2916 (C–H aromatic), 1242, 1165 cm^−1^; MS: *m/z* (%) = 902 (10) [*M* ^•+^], 634 (19), 588 (12), 491 (13), 457 (49), 455 (100), 427 (49), 394 (15), 307 (66), 206 (24), 128 (16), 108 (34), 90 (38), 77 (37), 64 (38); elemental analysis calcd (%) for C_38_H_34_Br_2_N_10_O_3_S_2_: C 50.56, H 3.80, N 15.52; found: C 50.71, H 3.67, N 15.41%.

### 3.18 2,2′‐(((((Oxybis(ethane‐2,1‐Diyl))bis(oxy))bis(4,1‐Phenylene))bis(methanylylidene))bis(hydrazin‐1‐Yl‐2‐Ylidene))bis(5‐((4‐Iodophenyl)diazenyl)‐4‐Methylthiazole) (20f)

Using 654 mg of hydrazonoyl halide **19f**, the *title compound* was obtained as a dark red solid, 76% yield; m.p. 170–171 °C; ^1^H NMR (300 MHz, [D_6_]DMSO): *δ* = 9.90 (s, 2H; NH), 8.50 (s, 2H; CH = N), 7.74–7.11 (16H; ArH), 4.16 (t, *J* = 4.5 Hz, 4H; CH_2_OAr), 3.83 (t, *J* = 4.5 Hz, 4H; CH_2_OCH_2_), 2.72 (s, 6H; 4,4′‐Me) ppm; IR: *ν*_max_ = 3441 (NH), 3035, 2924 (C–H aromatic), 1249, 1049 cm^−1^; MS: *m/z* (%) = 996 (32) [*M* ^•+^], 983 (40), 930 (32), 846 (67), 835 (65), 812 (52), 760 (44), 718 (45), 612 (41), 590 (38), 514 (75), 474 (93), 434 (50), 385 (100), 333 (33), 284 (62), 154 (90), 134 (99), 91 (42), 66 (54); elemental analysis calcd (%) for C_38_H_34_I_2_N_10_O_3_S_2_: C 45.79, H 3.44, N 14.05; found: C 45.96, H 3367, N 13.87%.

### 3.19 2,2′‐(((((Oxybis(ethane‐2,1‐Diyl))bis(oxy))bis(4,1‐Phenylene))bis(methanylylidene))bis(hydrazin‐1‐Yl‐2‐Ylidene))bis(4‐Methyl‐5‐((4‐Nitrophenyl)diazenyl)thiazole) (20g)

Using 483 mg of hydrazonoyl halide **19g**, the *title compound* was obtained as a dark red solid, 67% yield; m.p. 159–160 °C; ^1^H NMR (300 MHz, [D_6_]DMSO): *δ* = 9.84 (s, 2H; NH), 8.52 (s, 2H; CH = N), 8.15–6.98 (16H; ArH), 4.17 (t, *J* = 4.5 Hz, 4H; CH_2_OAr), 3.83 (t, *J* = 4.5 Hz, 4H; CH_2_OCH_2_), 2.76 (s, 6H; 4,4′‐Me) ppm; IR: *ν*_max_ = 3425 (NH), 3040, 2950 (C–H aromatic), 1319, 1249 cm^−1^; MS: *m/z* (%) = 834 (33) [*M* ^•+^], 773 (43), 756 (57), 745 (37), 683 (39), 677 (96), 645 (89), 510 (35), 468 (34), 462 (51), 442 (100), 418 (45), 383 (55), 259 (59), 202 (54), 154 (77), 111 (52), 85 (76); elemental analysis calcd (%) for C_38_H_34_N_12_O_7_S_2_: C 54.67, H 4.10, N 20.13; found: C 54.82, H 4.00, N 20.00%.

### 3.20 In Vitro Cytotoxic Antiproliferative Assay

The freshly produced bis‐heterocyclic compounds were investigated for antiproliferative potency toward four human tumor cell lines: HepG‐2 (liver), A‐549 (lung), HCT‐116 (colorectal carcinoma), and PC‐3 (prostatic adenocarcinoma) using the MTT assay. These cell lines were acquired from the American Type Culture Collection (ATCC, Manassas, VA, USA). The cells were cultured using Dulbecco's Modified Eagle Medium (DMEM) or RPMI 1640 Medium (Gibco, Thermo Fisher Scientific, Waltham, MA, USA). The sample medium was infused with 10% fetal bovine serum, L‐glutamine (2 mM), penicillin (100 U/mL), streptomycin (100 μg/mL), and amphotericin B (25 μg/mL) (Gibco, Thermo Fisher Scientific, Waltham, MA, USA). The cells were incubated at 37 °C in an environment of 5% CO_2_. The cells were inoculated into 96‐well flat‐bottom microtiter plates at a density of 5 × 10^3^ cells per well, utilizing 100 μL of growth medium. The samples were subsequently incubated for 24 h at 37 °C in a 5% CO_2_ atmosphere. Upon subsequent incubation, the cells were administered various concentrations of the compound under study (1–100 μM) in DMSO (Sigma‐Aldrich, Merck, Darmstadt, Germany) and brewed for 48 h. Following 48 h of exposure, MTT solution (20 μL at 5 mg/mL concentration) was introduced and maintained for 4 h. An equivalent volume of DMSO (0.1% (v/v)) was administered to the negative control wells containing untreated cells. Afterward, 100 μL of the solubilization solution was introduced into each well, and the microtiter plates were incubated overnight at 37 °C in a 5% CO_2_ environment to solubilize the purple formazan crystals. The absorbance of the samples was quantified at 570 nm wavelength using a plate reader (ELX 800, USA). Cell viability was assessed using the formula: (cell viability)% = (absorbance of treated cells/absorbance of untreated cells) × 100. The 50% inhibitory concentration (IC_50_) of any compound is the concentration that causes a 50% decrease in cell proliferation compared with untreated control cells. IC_50_ values were recorded as the mean ± standard deviation (SD) from three separate measurements using GraphPad Prism 9.0 software (GraphPad, Dotmatics, Boston, MA, USA).

### 3.21 Selectivity Index Determination

The selectivity of each compound was determined by evaluating their cytotoxic activity on WI38 (a normal fibroblast cell line) utilizing an MTT assay, as previously described. The selectivity index (SI) for each compound was determined by dividing the IC_50_ in the normal cell line (IC_50_‐normal) by the IC_50_ in the malignant cell line (IC_50_‐cancerous).

### 3.22 VEGFR‐2 and FGFR‐2 Inhibition Assay

The inhibitory efficacy of the most potent bis‐thiazolopyrimidine derivatives **8a** and **8b** against VEGFR‐2 and FGFR‐2 kinases was evaluated utilizing the VEGFR‐2 kinase assay kit (BPS Bioscience, Catalog no. 40 325, San Diego, CA, USA) and the FGFR‐2 kinase assay kit (BPS Bioscience, Catalog no. 79 804, San Diego, CA, USA), following the guidelines recommended by the manufacturer. Three separate experiments were performed for each compound, and the mean ± standard deviation was determined.

### 3.23 Molecular Docking

Molecular computations of the most active bis‐heterocyclic derivatives **8a** and **8b** in inhibiting the activities of VEGFR‐2 and FGFR‐2 were performed using AutoDock 4.2 to investigate their binding interactions and to explore their binding modes. Crystal structures of both VEGFR‐2 (PDB ID: 2OH4) and FGFR‐2 (PDB ID: 7KIA) were acquired from the Protein Data Bank. Default AutoDock settings were used. The docking procedure was accomplished using a Lamarck Genetic Algorithm (LGA). The ligand's starting position, alignment, and torsions were randomly appointed, and every rotatable torsion was then allowed to relax. Every ligand experienced 100 docking runs, with a maximum of 2.5 × 10^6^ energy calculations recorded for every run. The collected data were evaluated using Discovery Studio Visualizer 2.5.

### 3.24 In Silico Pharmacokinetics and Drug‐Likeness Evaluation

The newly synthesized bis‐heterocyclic compounds were further screened to assess their pharmacokinetic characteristics. Physicochemical and pharmacokinetic parameters, medicinal chemistry compatibility, drug‐like characteristics, and ADMET were projected and estimated using the online tools SwissADME (http://www.swissadme.ch) and pkCSM software.

## 4 Conclusions

The design of a large group of diethylene glycol‐linked bis‐imidazo/thiazolo‐fused mono‐, bi‐, and tricyclic derivatives was accomplished. A simple, efficient synthesis accessed the bis‐heterocyclic templates using inexpensive commercially available materials to construct two building blocks, bis‐phenacyl bromide derivative **4** and bis‐thiosemicarbazone derivative **6**. From these precursors, diverse bis‐heterocyclic compounds were prepared efficiently, including bis(thiazolo[3,2‐*a*]pyrimidinone) derivatives **8a** and **8b**, bis(imidazo) derivatives **12‐14**, bis(imidazo[2,1‐*b*]thiazole **17** and its benzo[*d*] analog **18** and a small library of bis(thiazole) derivatives **20a‐g**.

All new bis‐heterocyclic templates were evaluated for cytotoxic activity against four diverse human tumor cell lines, HepG‐2 (liver), A‐549 (lung), HCT‐116 (colon), and PC‐3 (prostate), and their selectivity established using normal fibroblasts (WI‐38) with cisplatin as a reference standard. The diethylene glycol‐linked bis‐heterocycles exhibited greater cytotoxicity and remarkable selectivity to hepatocellular carcinoma (HepG‐2) than the other three cancer cell lines. With promising cytotoxicity against HepG‐2, the most potent bis‐imidazo/thiazolo‐fused derivatives **8a**, **8b**, **12**, and **13** showed IC_50_ values of 6.8–17.8 µM, with exceptional selectivity against HepG‐2 over normal cells, with selectivity indices of SI 5.6–14.6 compared with SI of 2.4 for cisplatin itself. Displaying the highest cytotoxicity against HepG‐2, along with exceptional selectivity, bis(thiazolo[3,2‐*a*]pyrimidinone) derivatives **8a** and **8b** were submitted to further mechanistic investigation as VEGFR‐2 TK and /or FGFR‐2 TK inhibitors. Derivative **8a** had a moderate anti‐VEGFR‐2 and anti‐FGFR‐2 activity, with IC_50_ 0.61±0.02 and 0.27±0.01 µM, respectively, whereas the phenyl‐substituted derivative **8b** displayed respectable anti‐VEGFR‐2 activity and remarkable anti‐FGFR‐2 activity, with IC_50_ 0.19 ± 0.01 and 0.03 ± 0.01 µM, measured against the standard drugs sorafenib and staurosporine (IC_50_ 0.12 ± 0.01 and 0.03 ± 0.01 µM, respectively).

With satisfactory binding scores and RMSDs of 0.54 and 1.2 Å for the active sites of VEGFR‐2 and FGFR‐2, respectively, molecular docking experiments of the two most potent bis(thiazolo[3,2‐*a*]pyrimidinones) **8a** and **8b** with VEGFR‐2 and FGFR‐2 TKs were successful. Docking simulations were performed on the ATP active pockets of the 3D crystal structures (PDB ID: 2OH4) and (PDB ID: 7KIA) of the VEGFR‐2 and FGFR‐2 kinases, respectively, using the co‐crystallized drugs sorafenib and staurosporine as standard ligands, respectively. The binding energy score of derivative **8b** reflected its high affinity for the VEGFR‐2 and FGFR‐2 ATP binding pockets, which is consistent with the experimental results. Pharmacokinetics and ADMET drug‐likeness evaluation indicated that both bis(thiazolo[3,2‐*a*]pyrimidinone) derivatives **8a** and **8b** have drug‐like characteristics.

A comprehensive review of the biological properties of these novel bis‐imidazo‐ and thiazolo‐fused derivatives as well as further functional improvements is presently under full consideration.

## Conflicts of Interest

The authors declare no conflicts of interest.

## Supporting information

Supplementary Material

## Data Availability

The data that supports the findings of this study are available in the Supporting Information of this article.
