# Supplementary material for: Diethylene Glycol‐Linked Ring‐Fused Bis‐Azoles: Target‐Oriented Synthesis, In Silico Screening, and Antitumor Activity
Source: ChemMedChem. 2026 Sep 29;21(19):e70504. doi: 10.1002/cmdc.70504 (PMC13624519; doi:10.1002/cmdc.70504)
Supplement: Supplementary file 1 [file CMDC-21-e70504-s001.pdf]

Diethyl Ether-linked Bis-Imidazo-Fused Heterocyclic Anticancer Prototypes: Target-Oriented Synthesis, In Silico Assessment and Cytotoxic Evaluation in Hepatocellular Carcinoma (HepG2) Cells

Sami A. Al-Hussain[1], Abdulrahman S. Alharbi[2], Zeinab A. Muhammad[3], Alyaa S. Abdel Halim[4], Magdi E. A. Zaki[1], Mark C. Bagley*[5], Refaie M. Kassab[6]

[1]Department of Chemistry, Faculty of Science, Imam Mohammad Ibn Saud Islamic University (IMSIU), Riyadh 11623, KSA
[2]Department of Chemistry, College of Science and Humanities-Dawadmi, Shaqra University, Saudi Arabia
[3]Department of Pharmaceutical Chemistry, Egyptian Drug Authority (EDA), Giza 12311, Egypt
[4]Department of Biochemistry, Faculty of Science, Ain Shams University, Cairo 11566, Egypt
[5]Department of Chemistry, University of Sussex, Falmer, Brighton, East Sussex, BN1 9QJ, UK
[6]Department of Chemistry, Faculty of Science, Cairo University, Giza 12613, Egypt

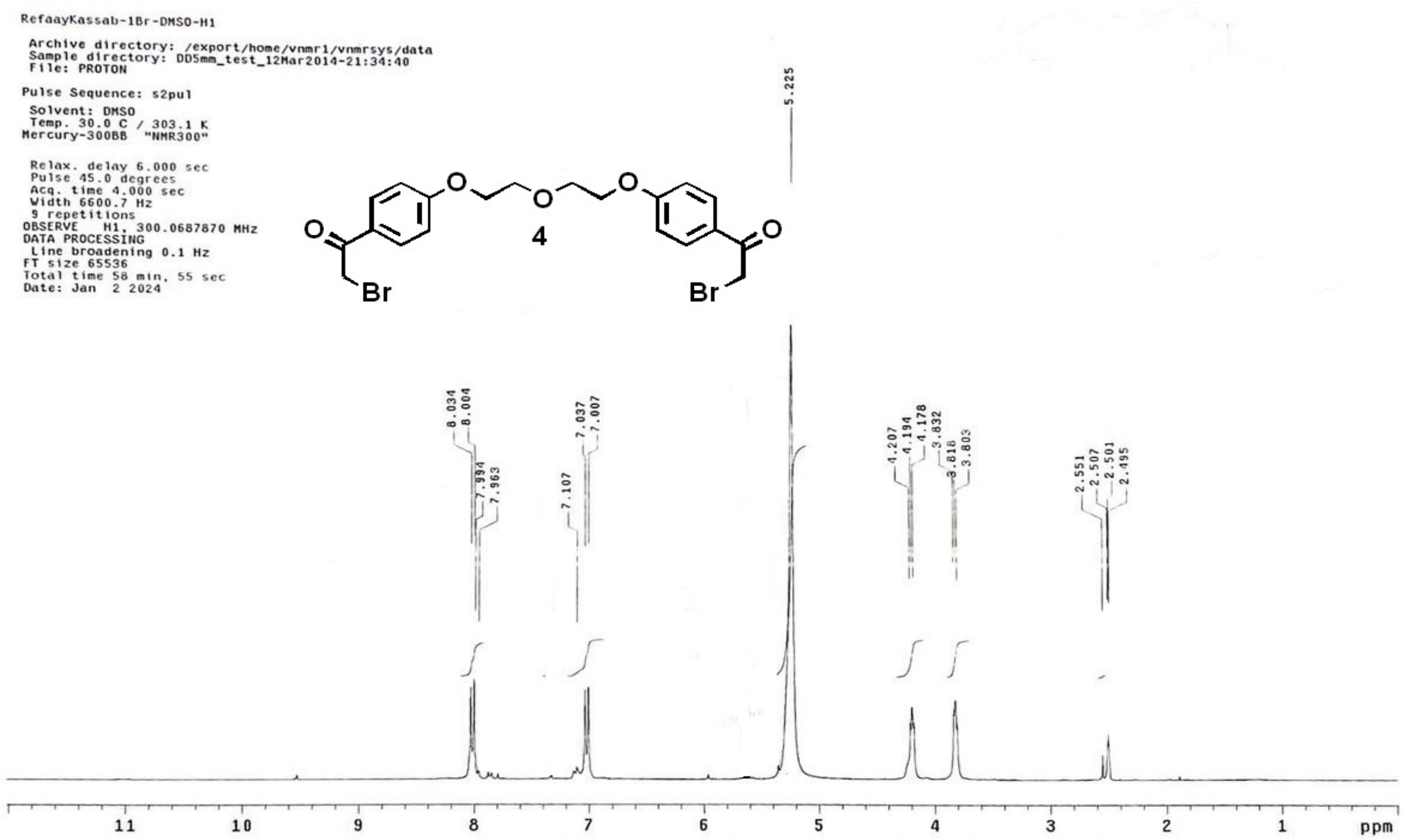

^{1}H NMR spectrum compound 4

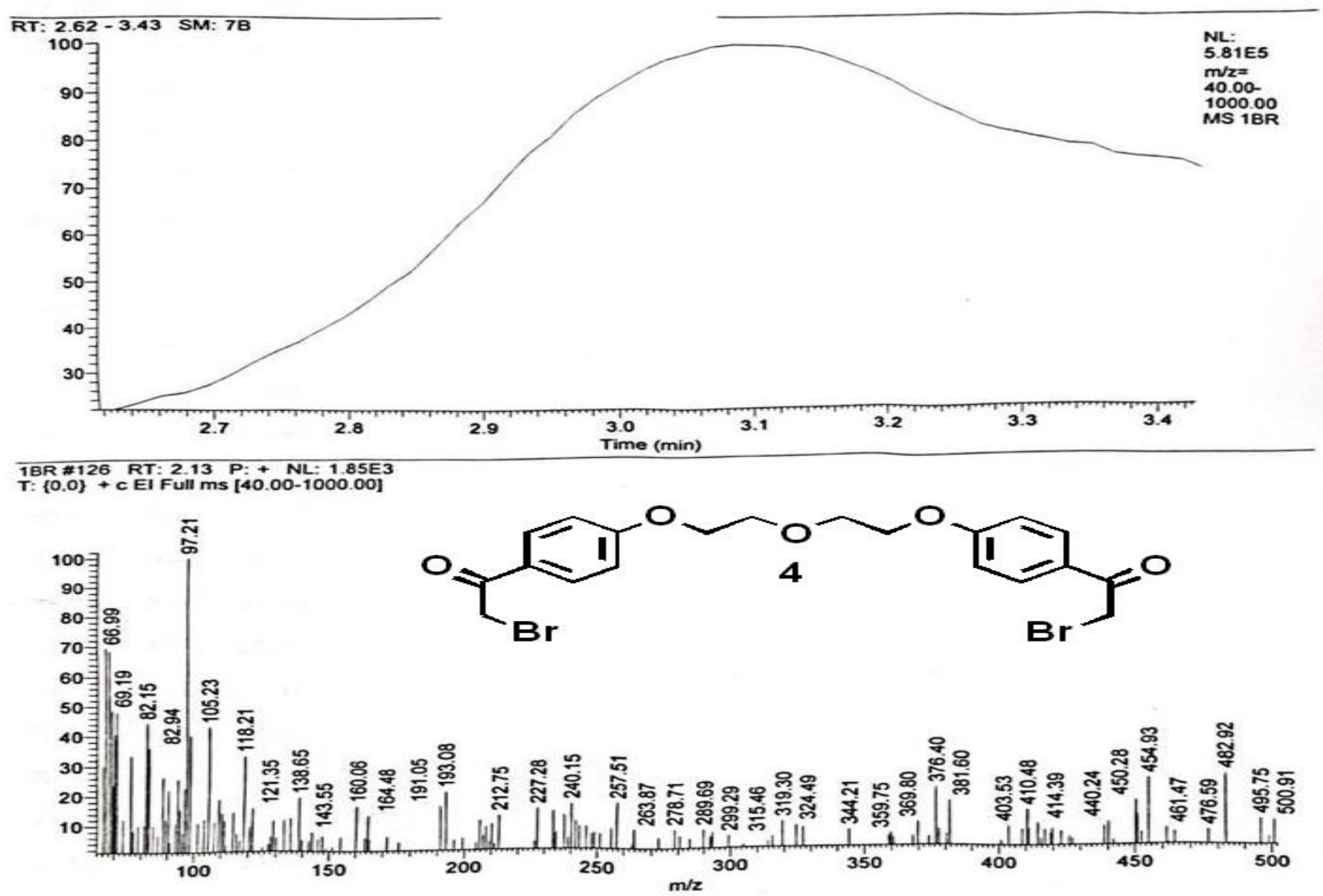

Mass spectrum compound 4

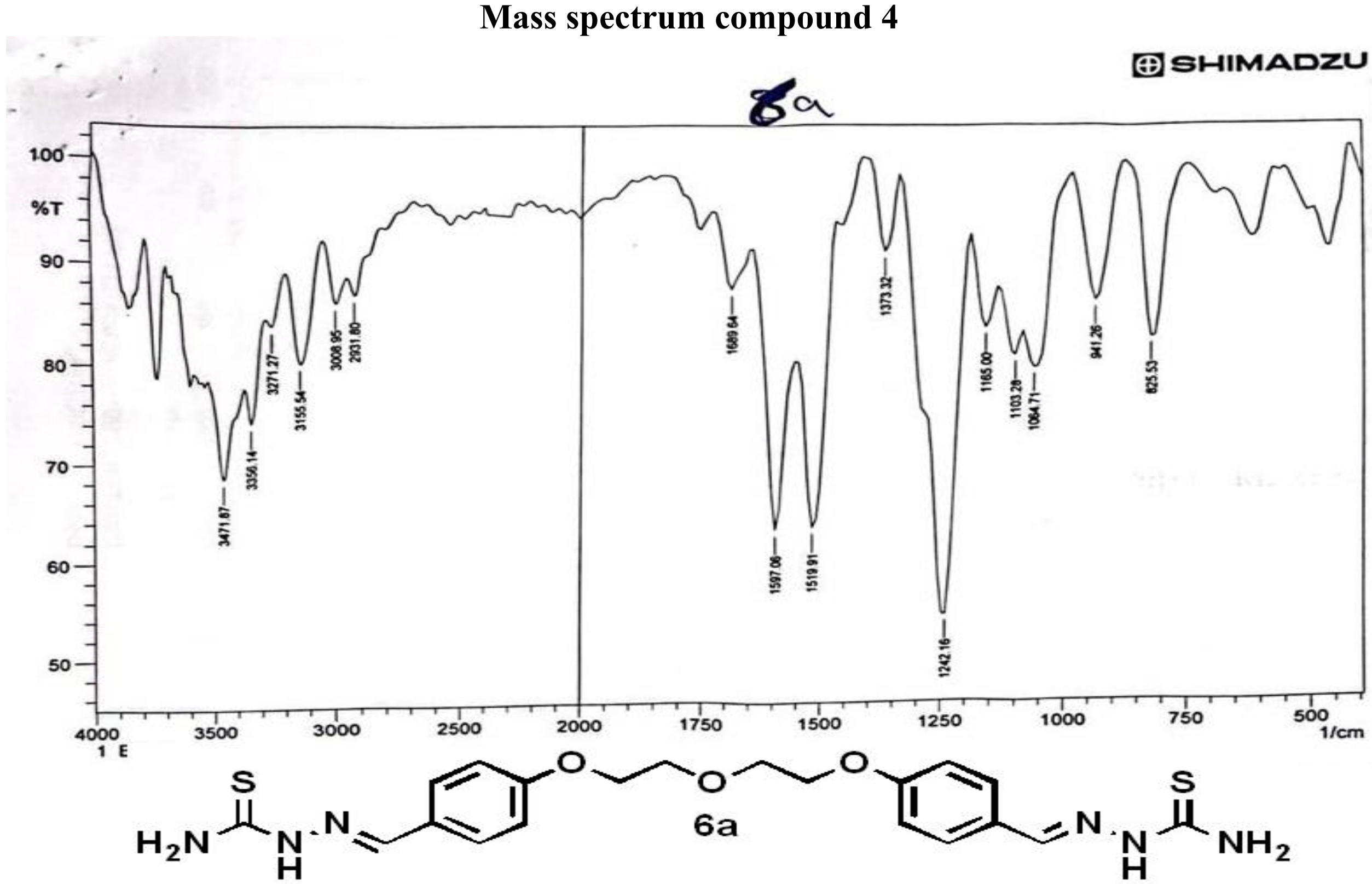

IR spectrum compound 6a

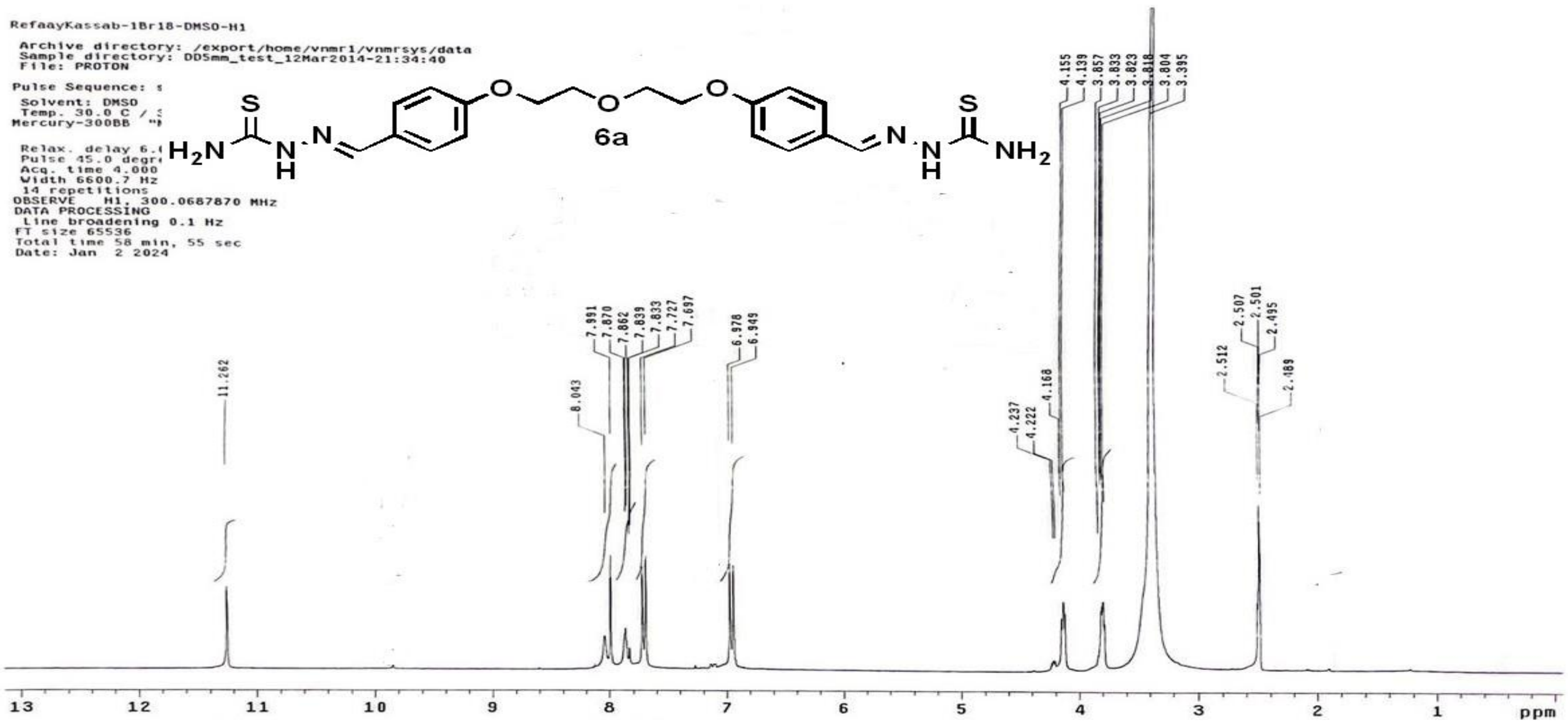

^{1}H NMR spectrum compound 6a

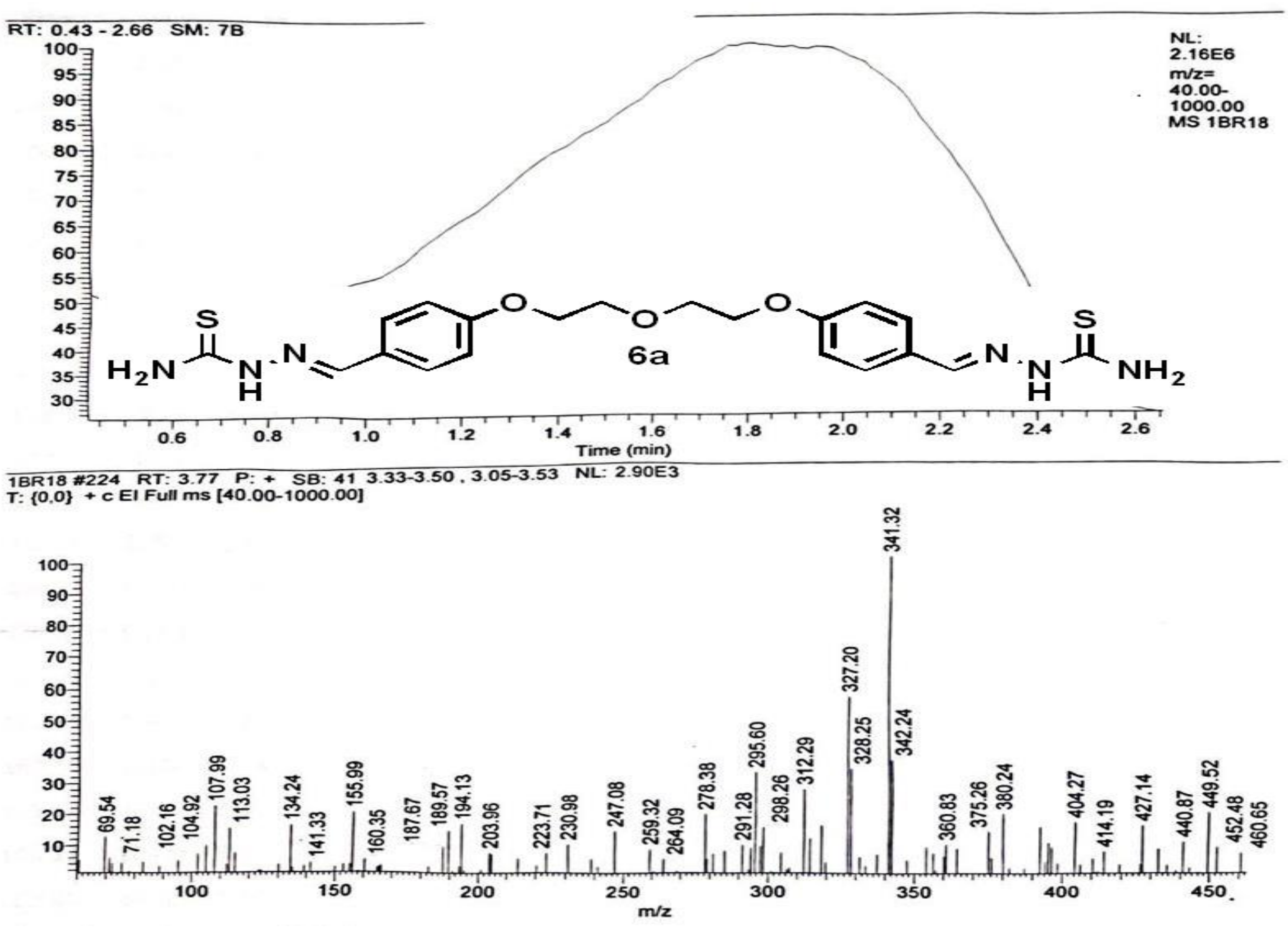

Mass spectrum compound 6a

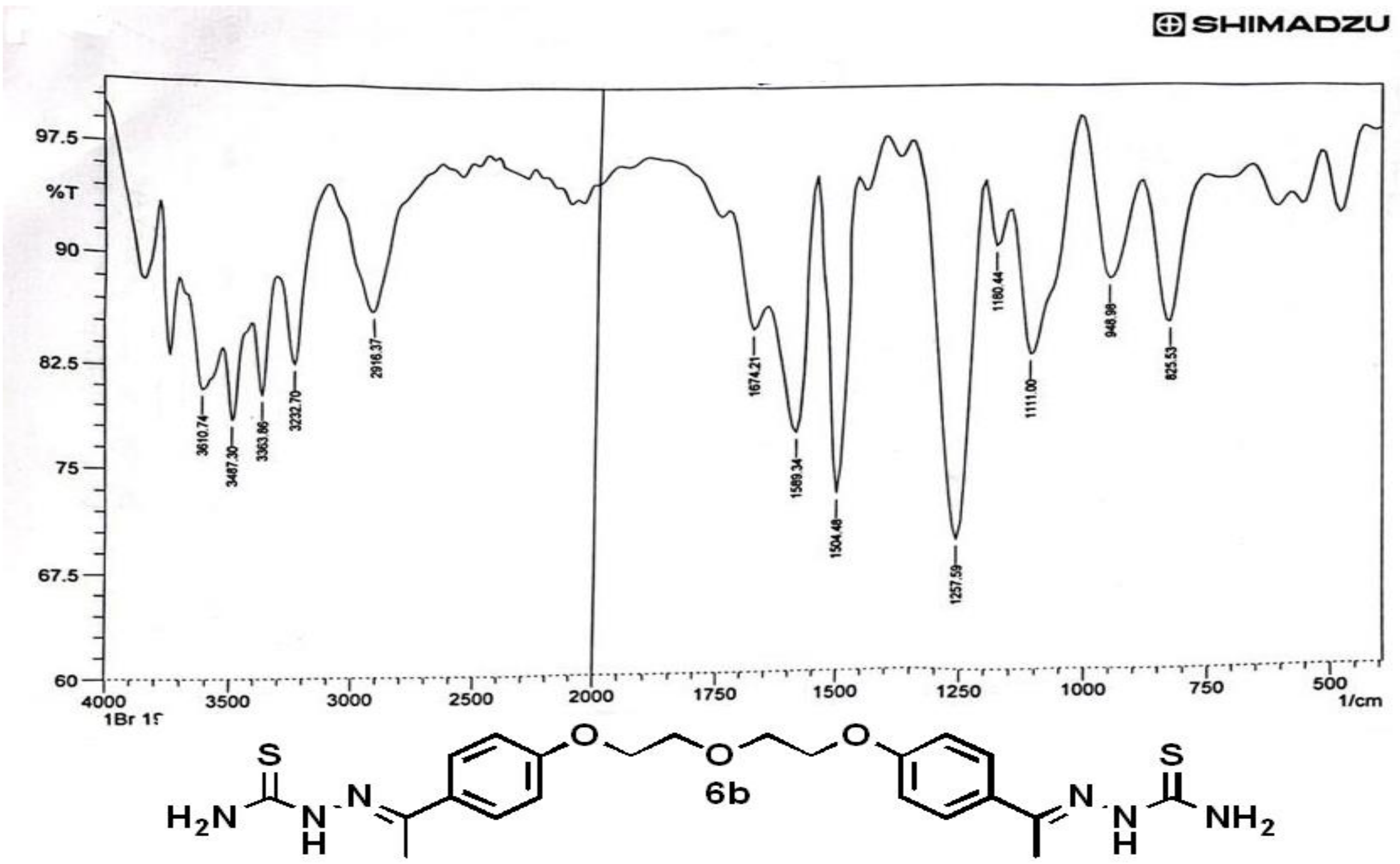

IR spectrum compound 6b

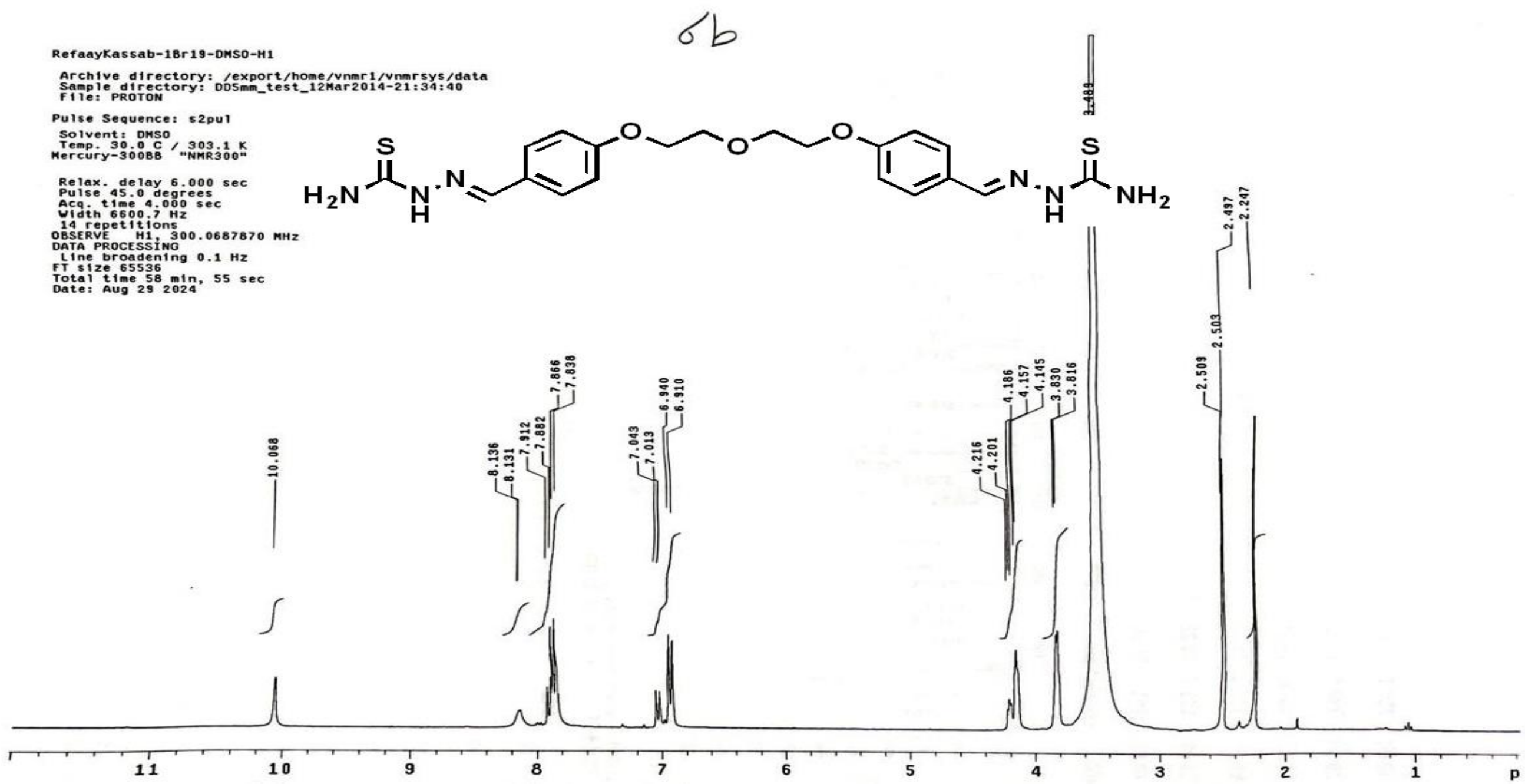

^{1}H NMR spectrum compound 6b

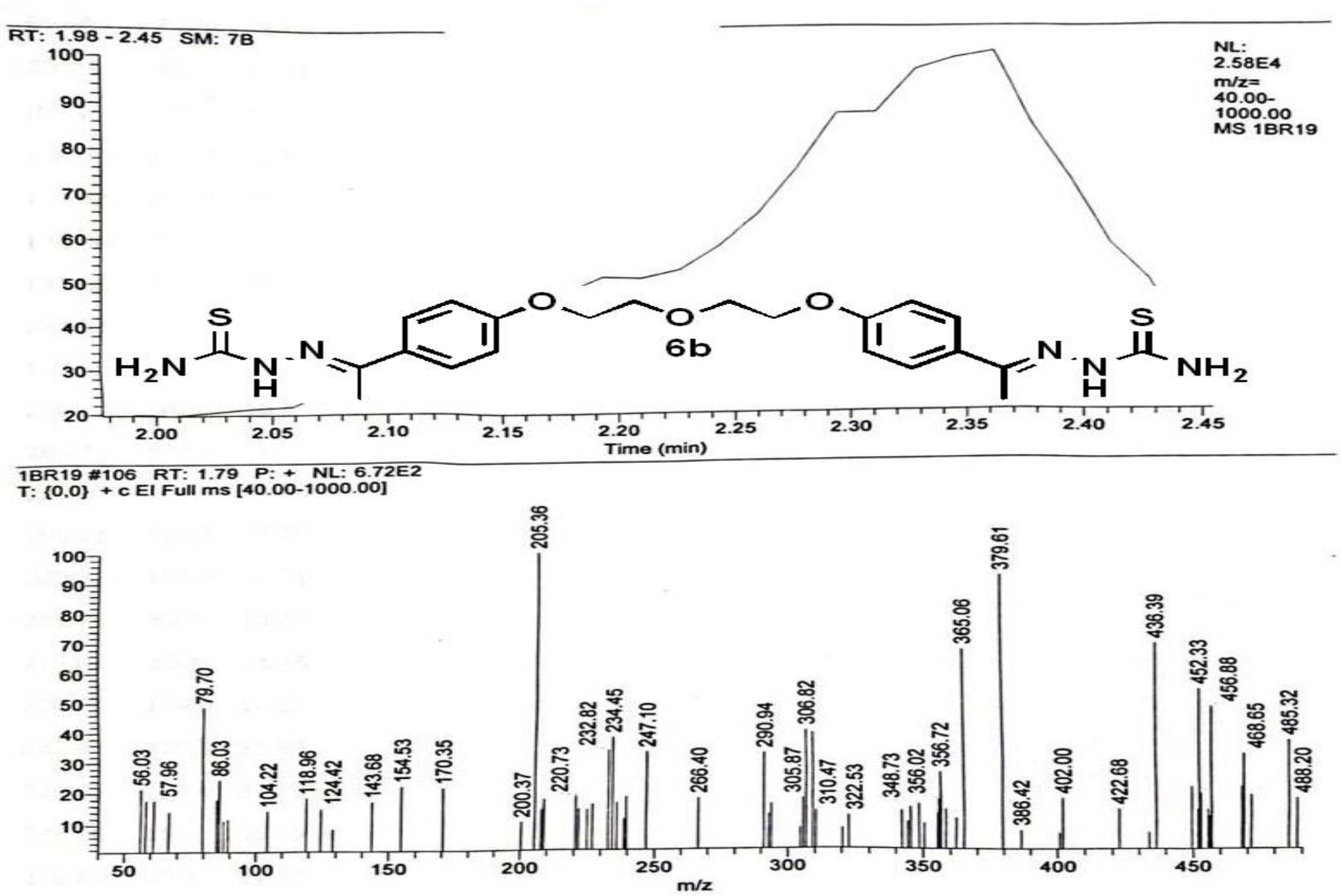

Mass spectrum compound 6b

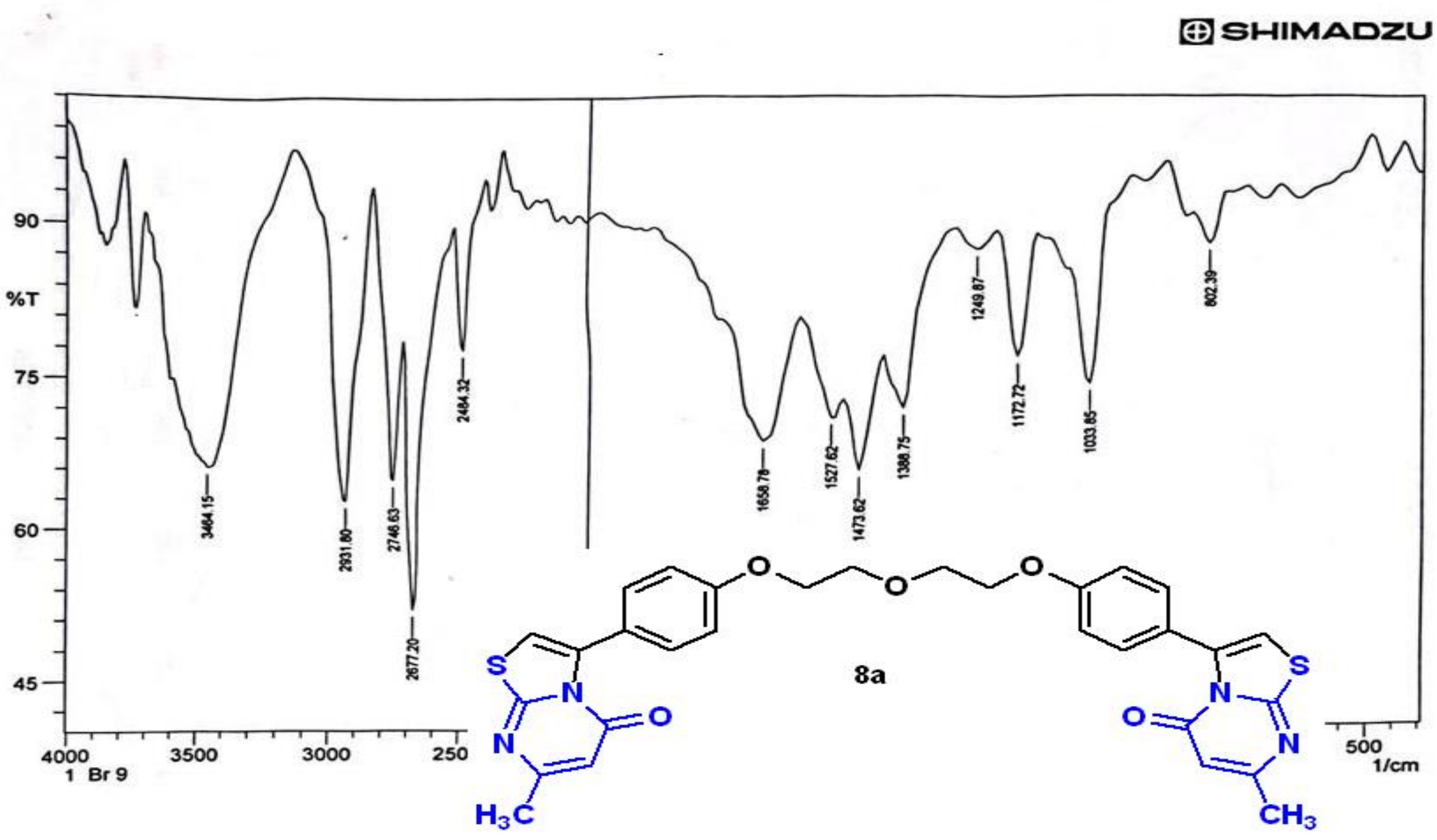

IR spectrum compound 8a

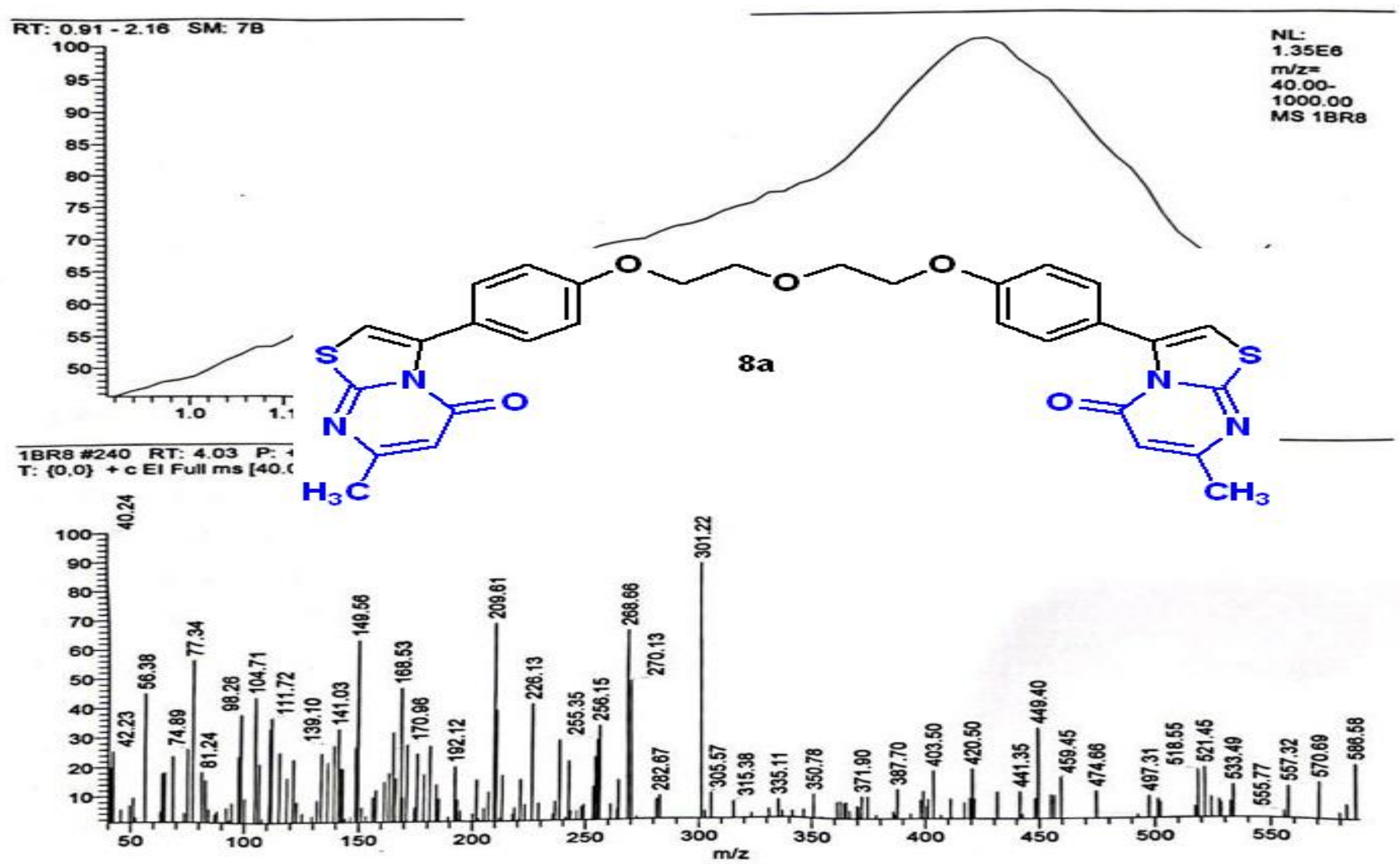

Mass spectrum compound 8a

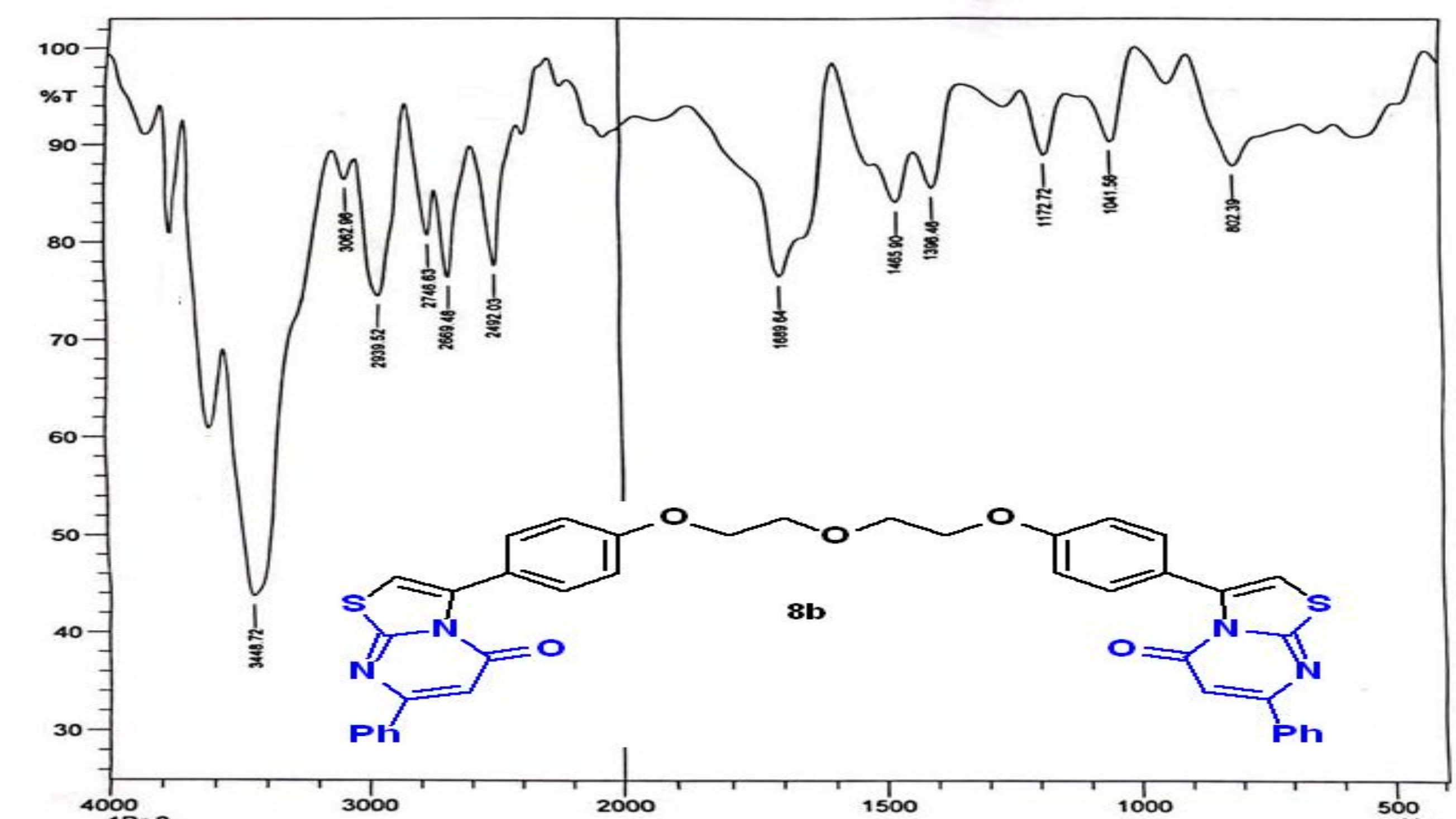

IR spectrum compound 8b

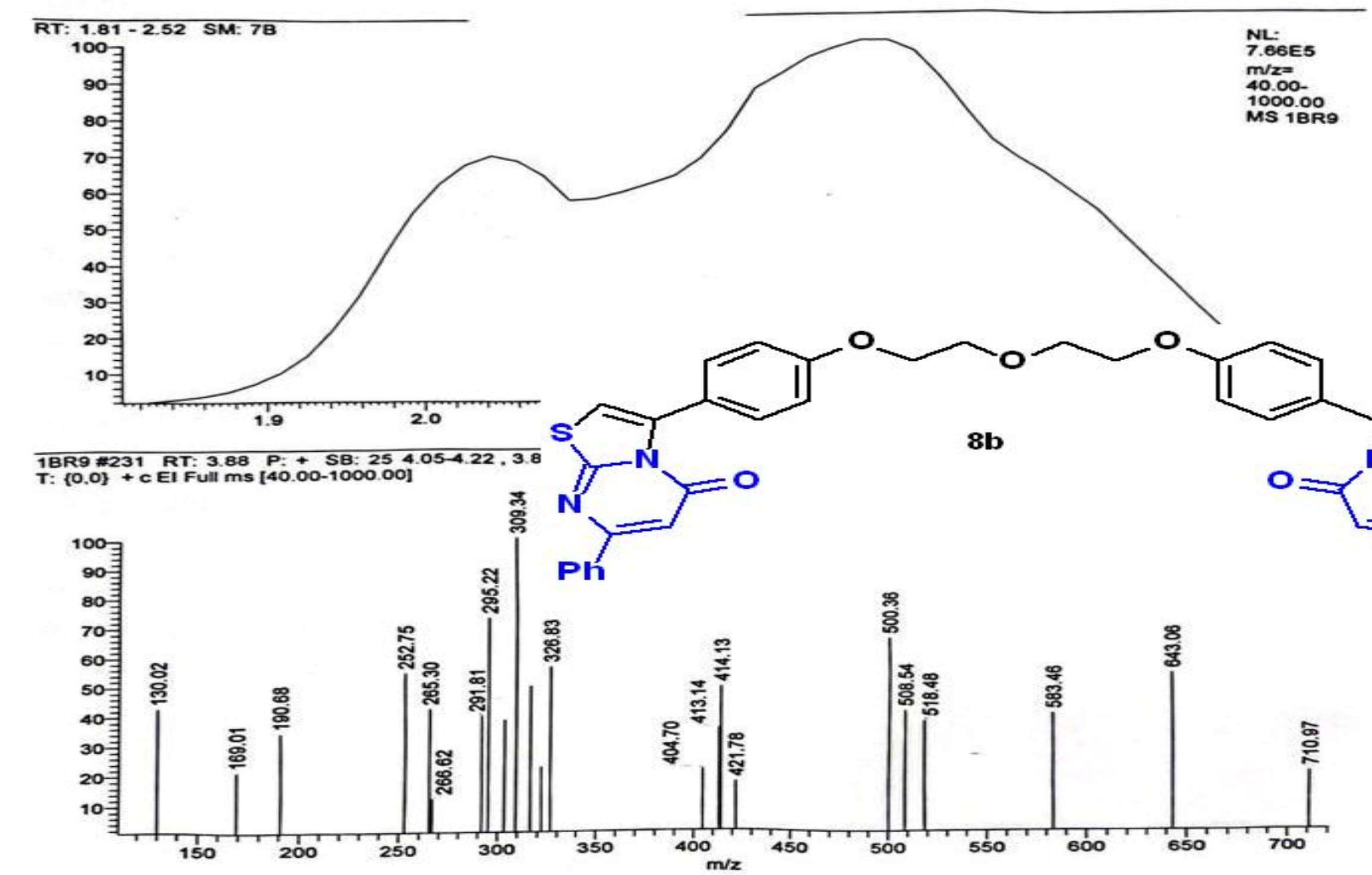

Mass spectrum compound 8b

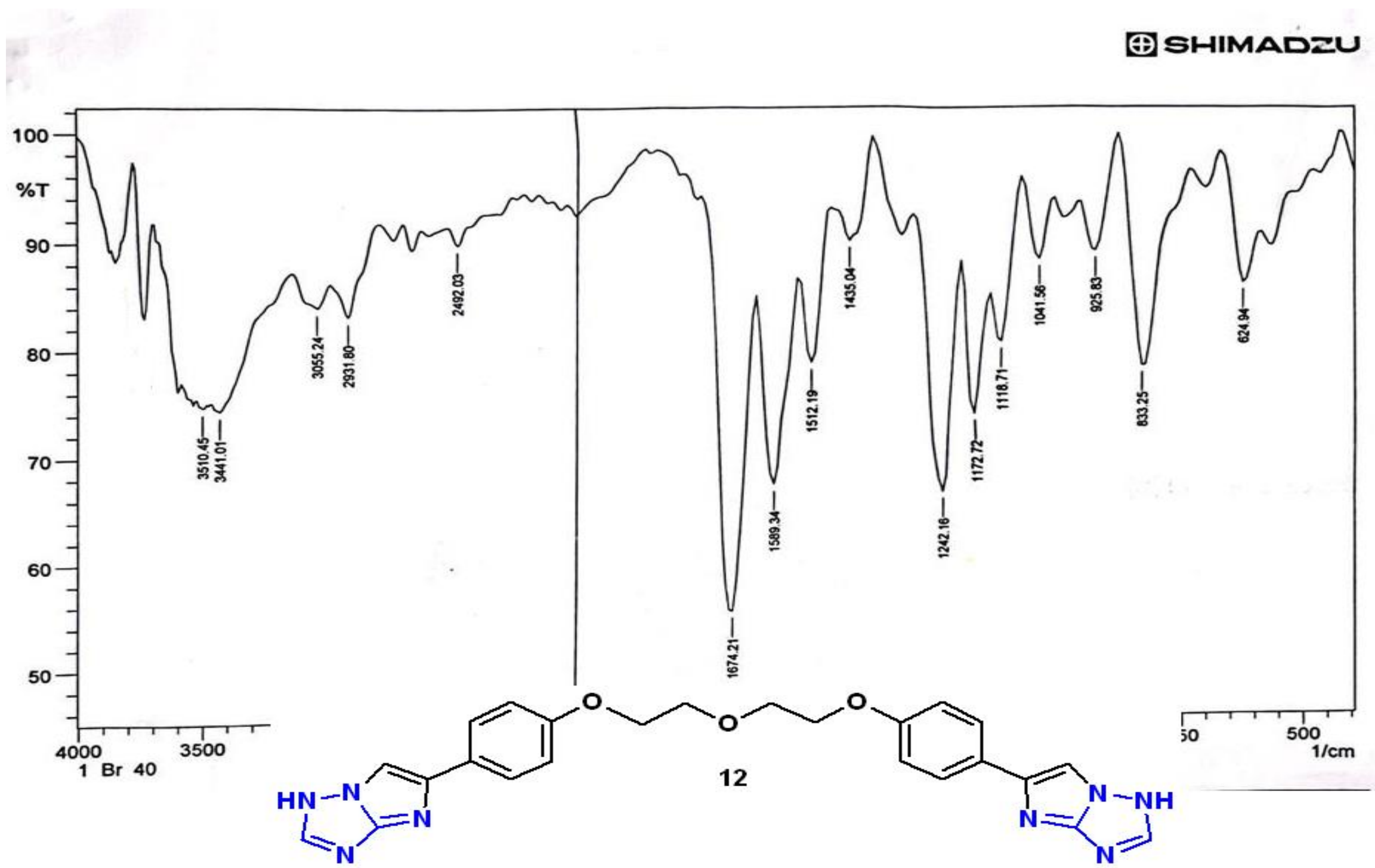

IR spectrum compound 12

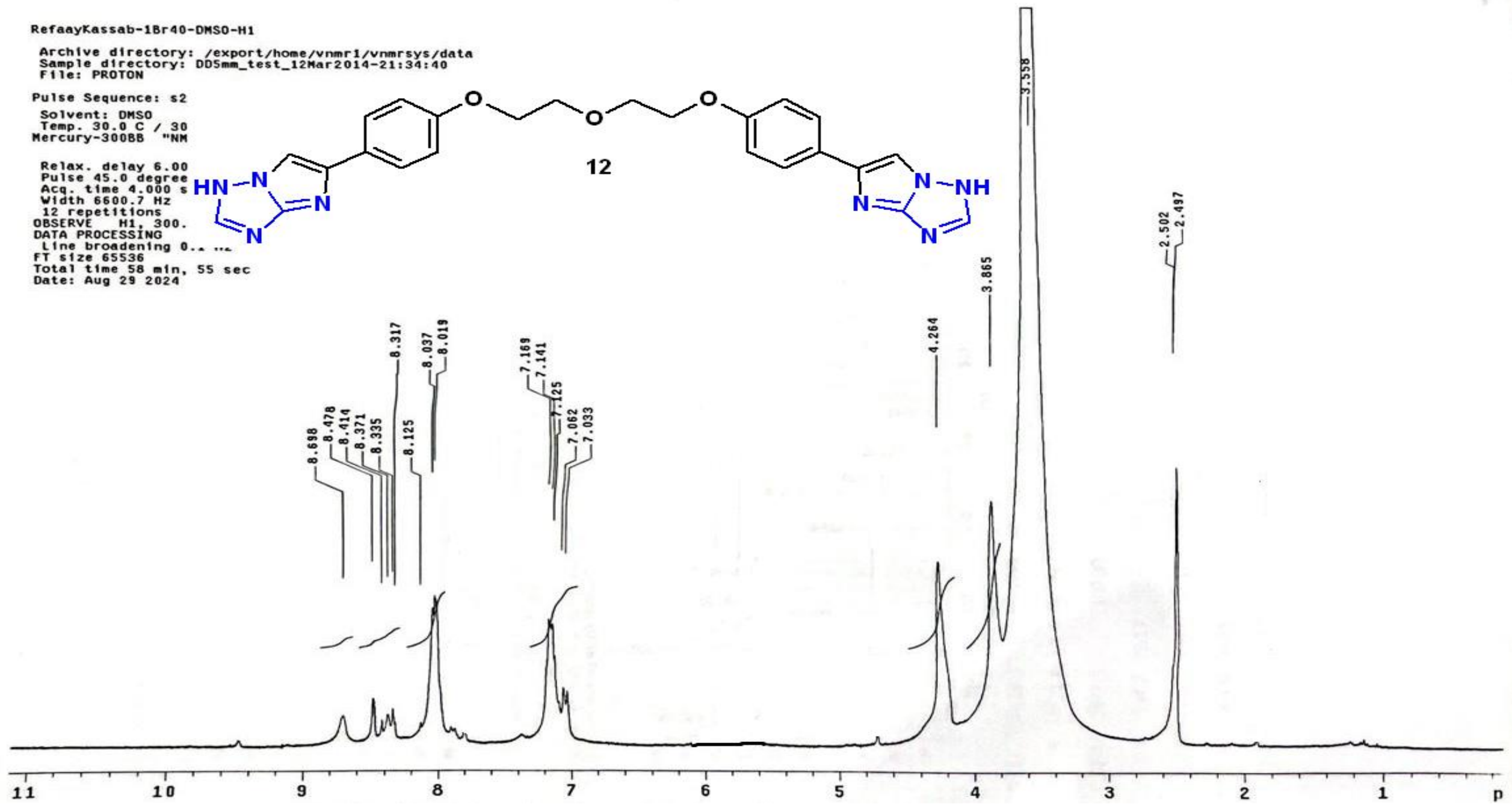

^{1}H NMR spectrum compound 12

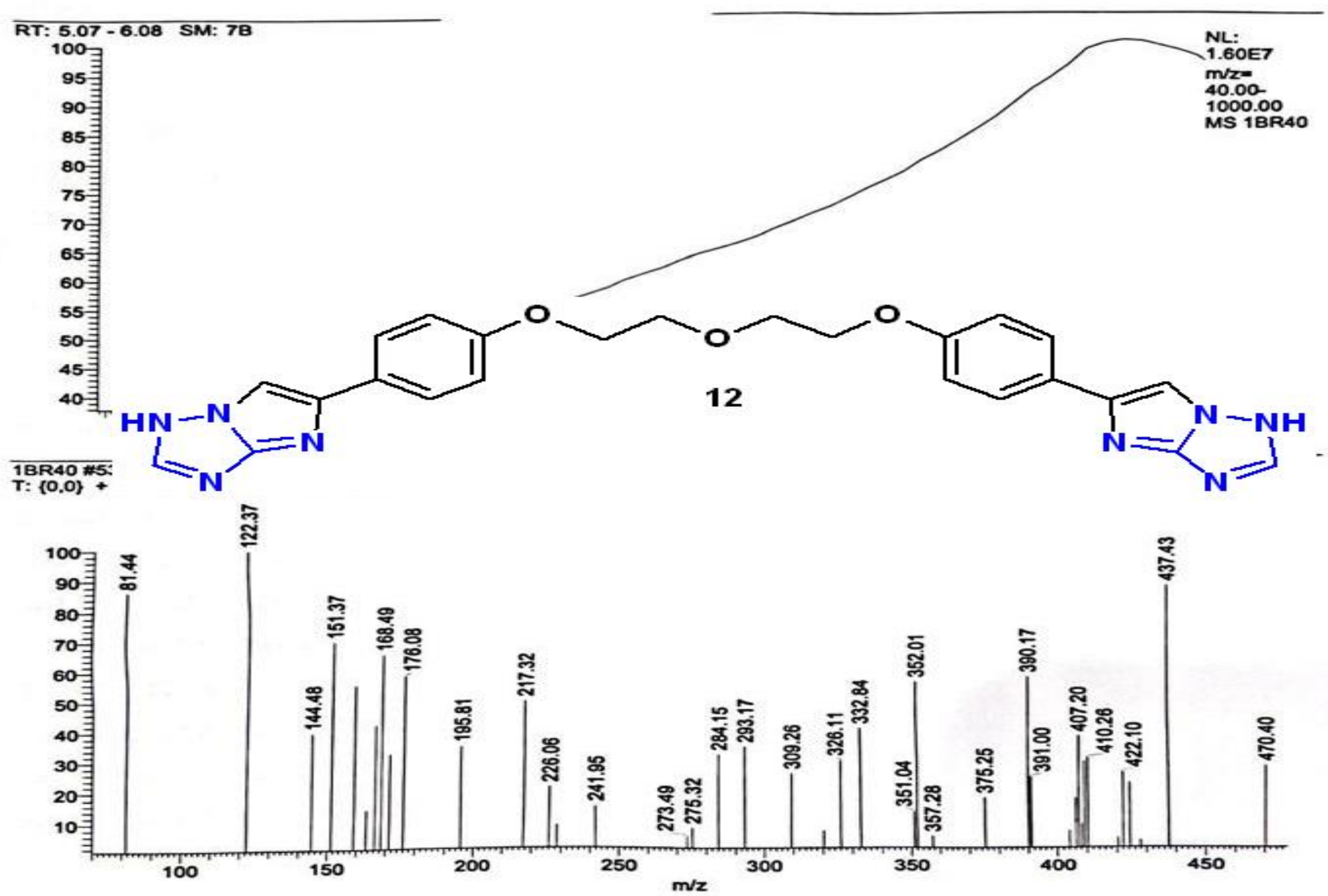

Mass spectrum compound 12

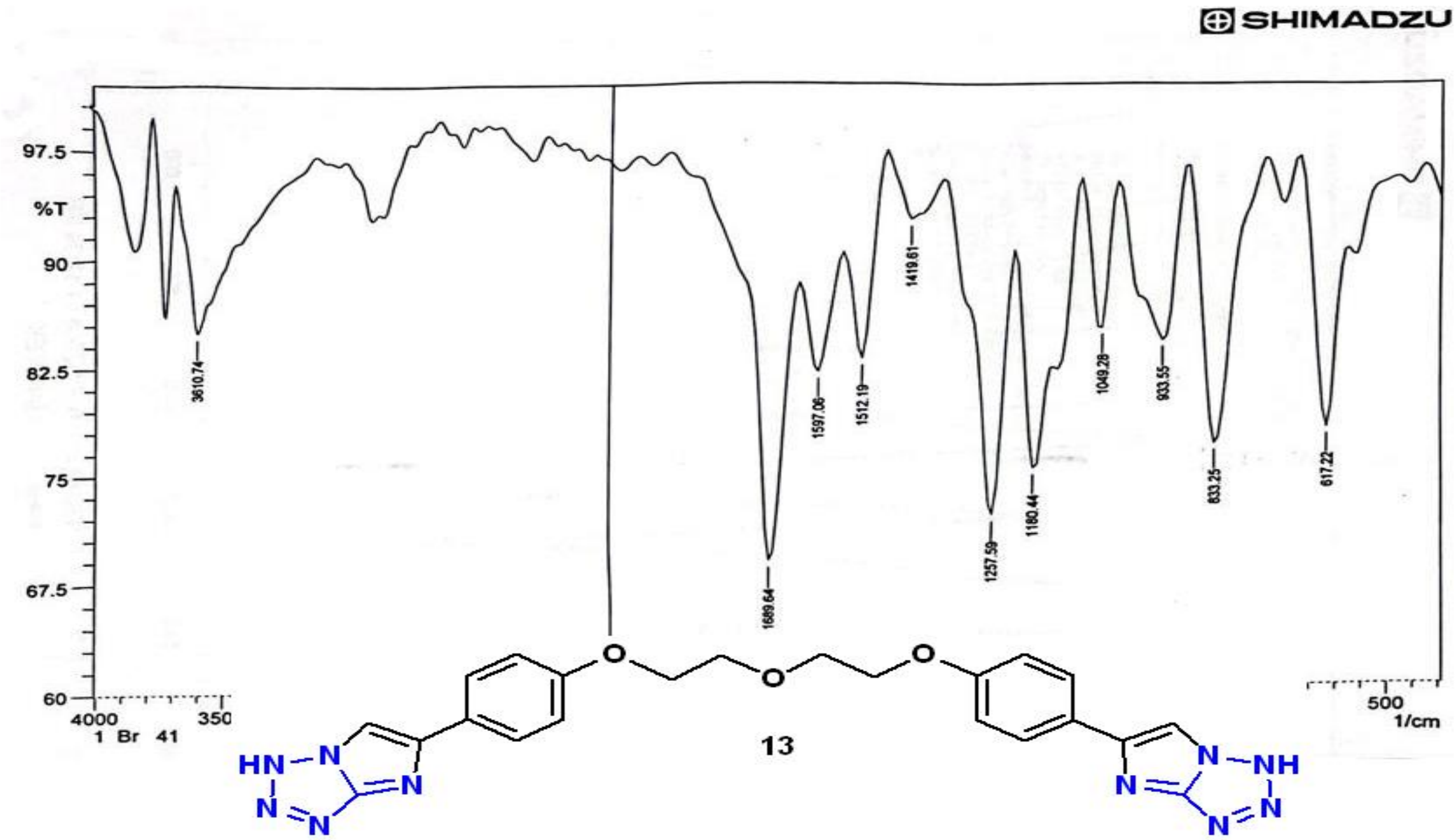

IR spectrum compound 13

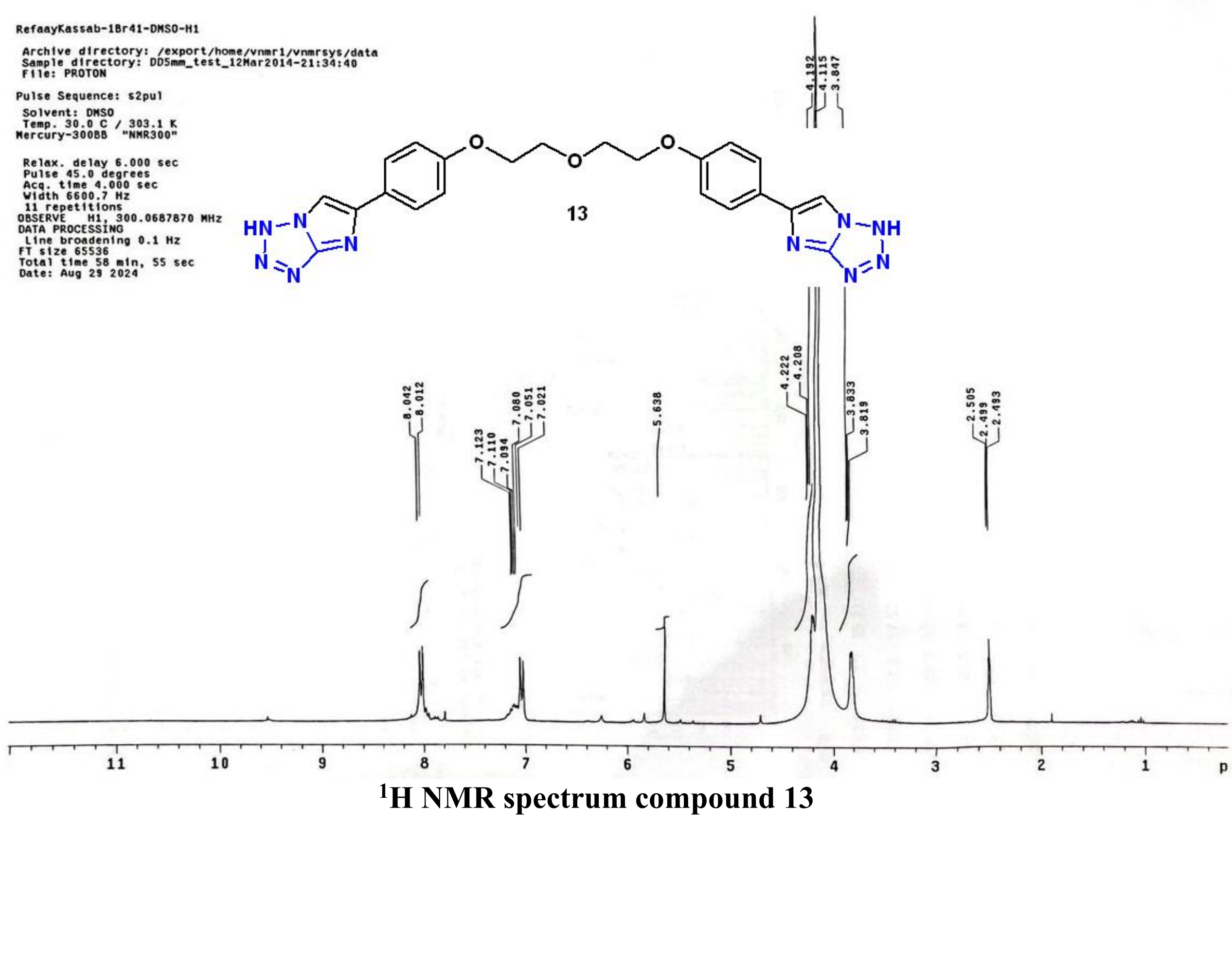

^{1}H NMR spectrum compound 13

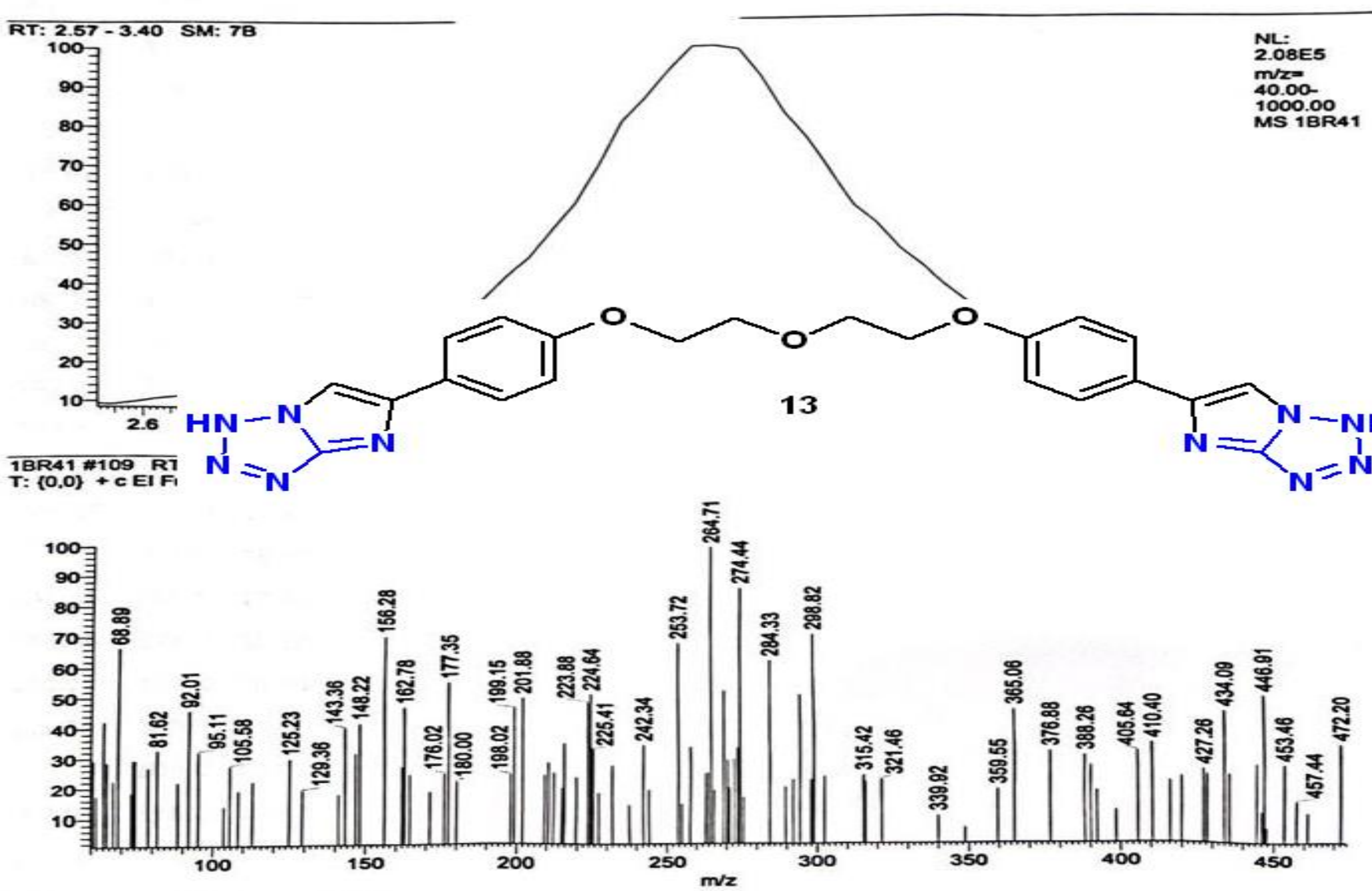

Mass spectrum compound 13

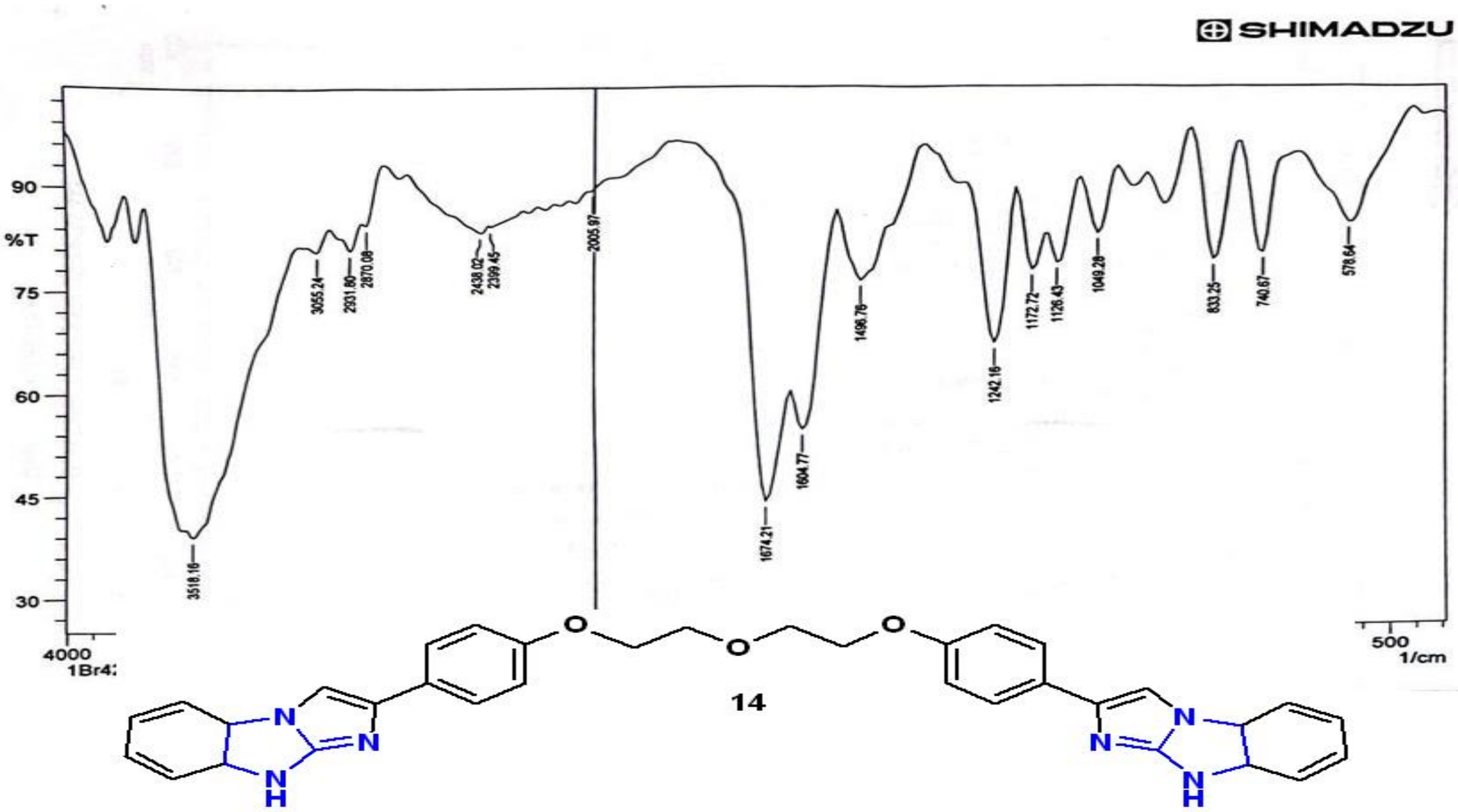

IR spectrum compound 14

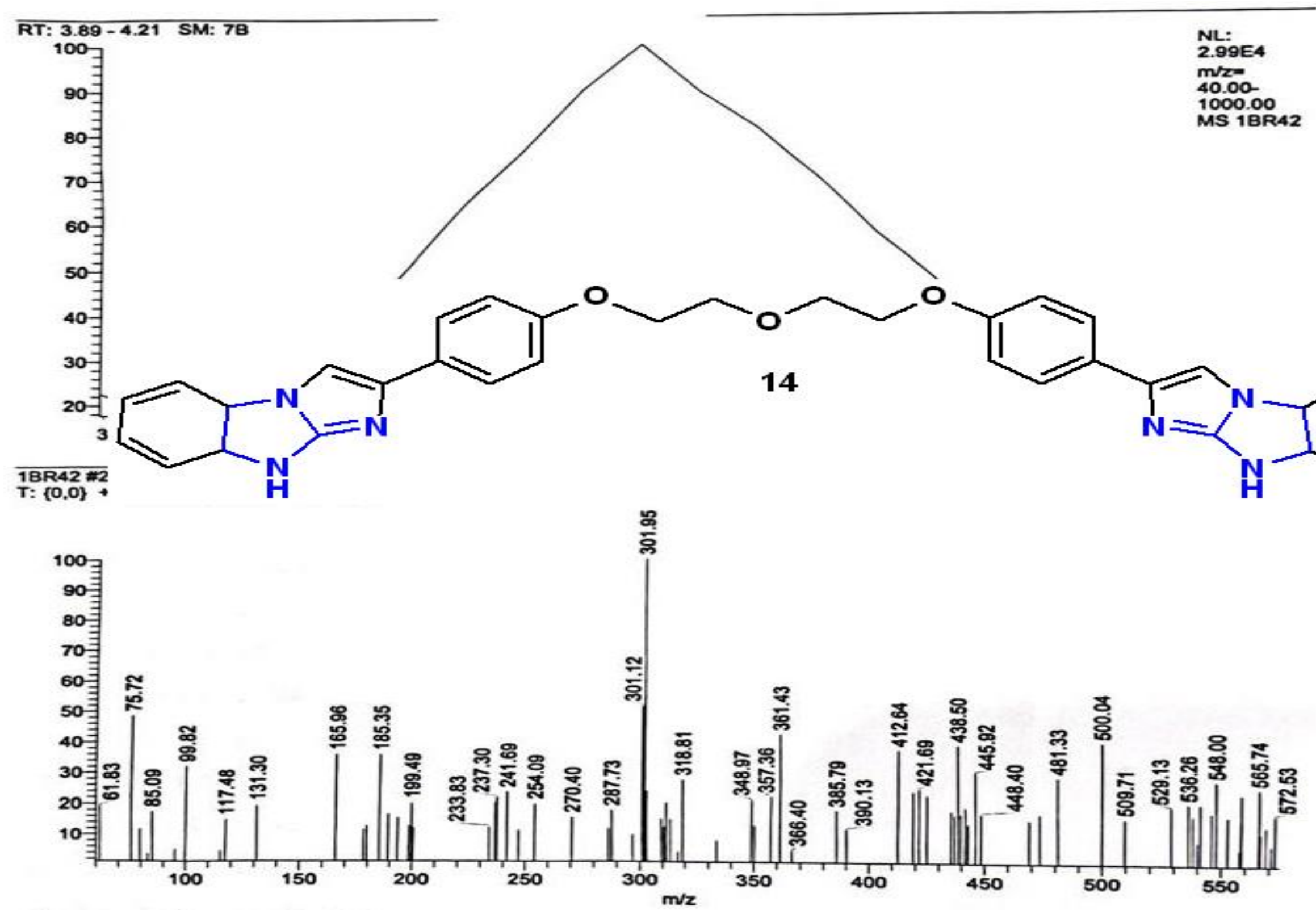

Mass spectrum compound 14

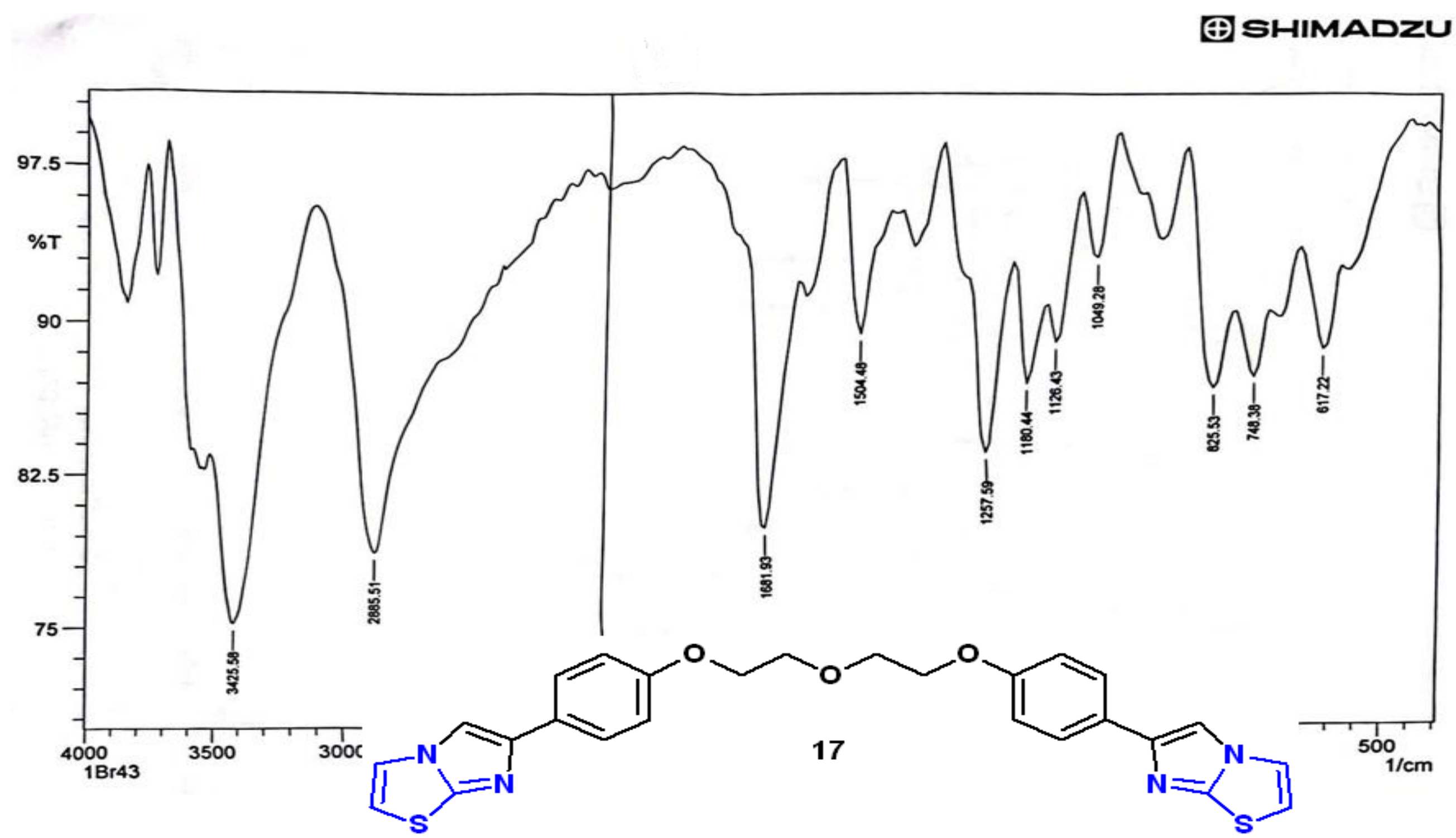

IR spectrum compound 17

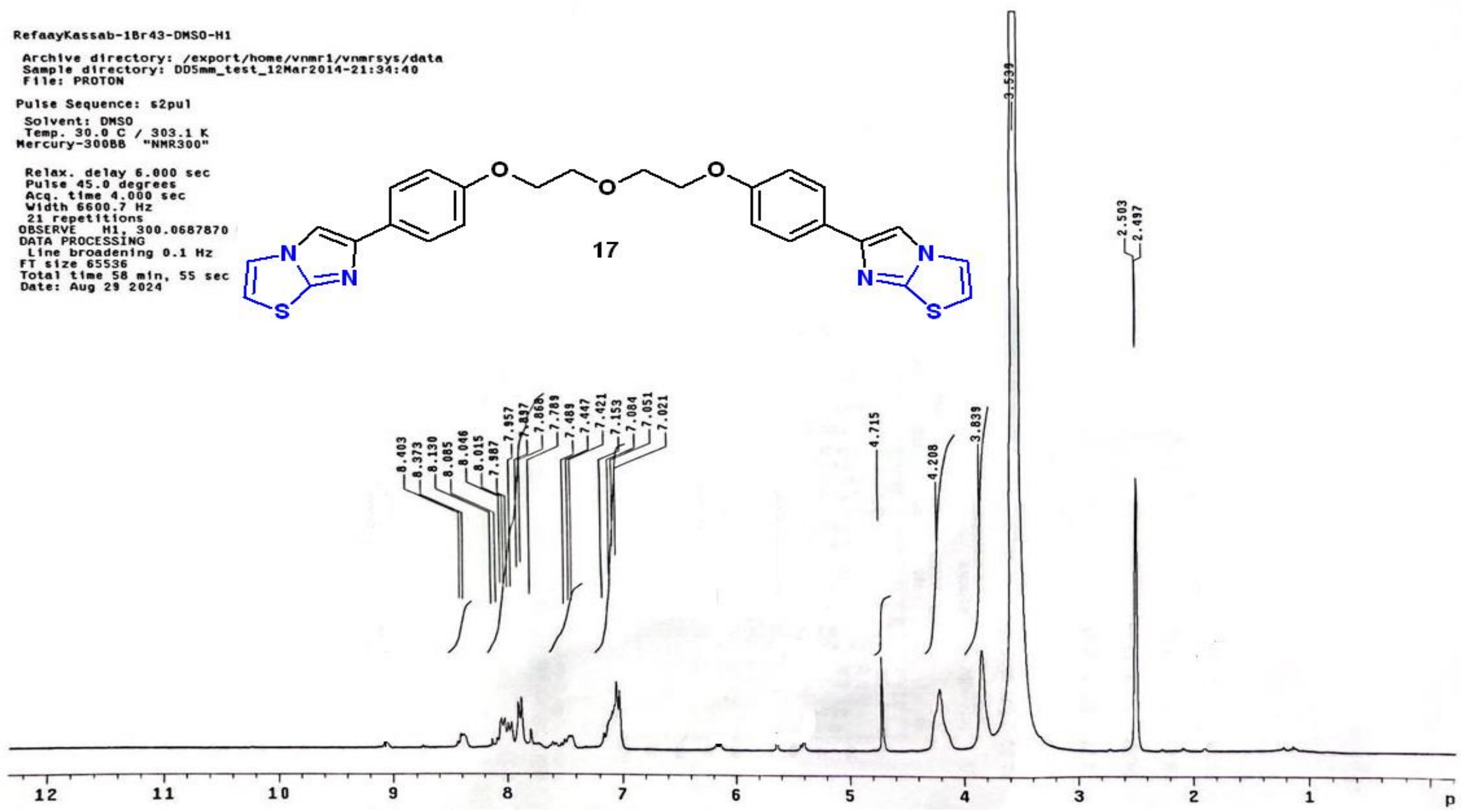

^{1}H NMR spectrum compound 17

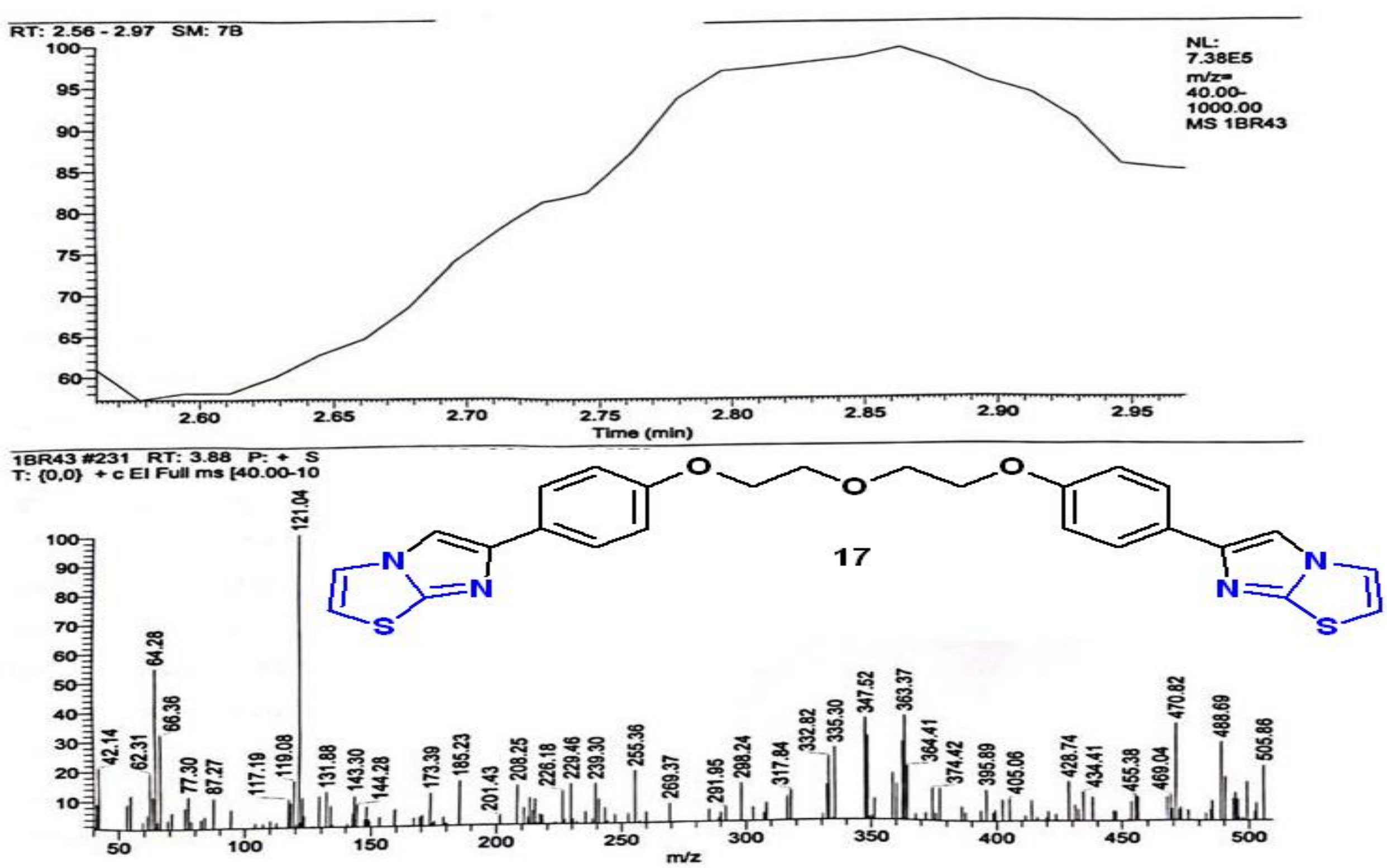

Mass spectrum compound 17

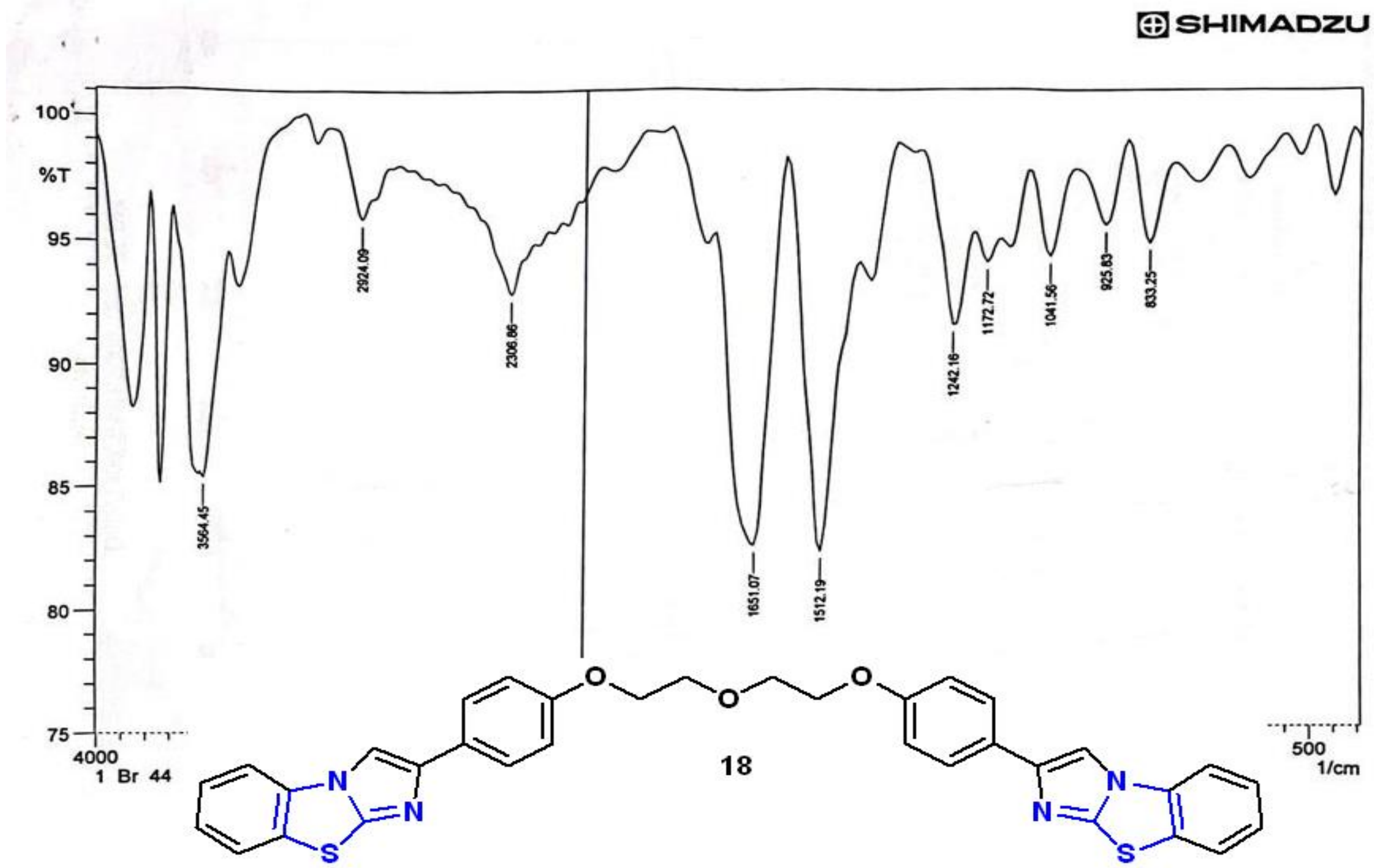

IR spectrum compound 18

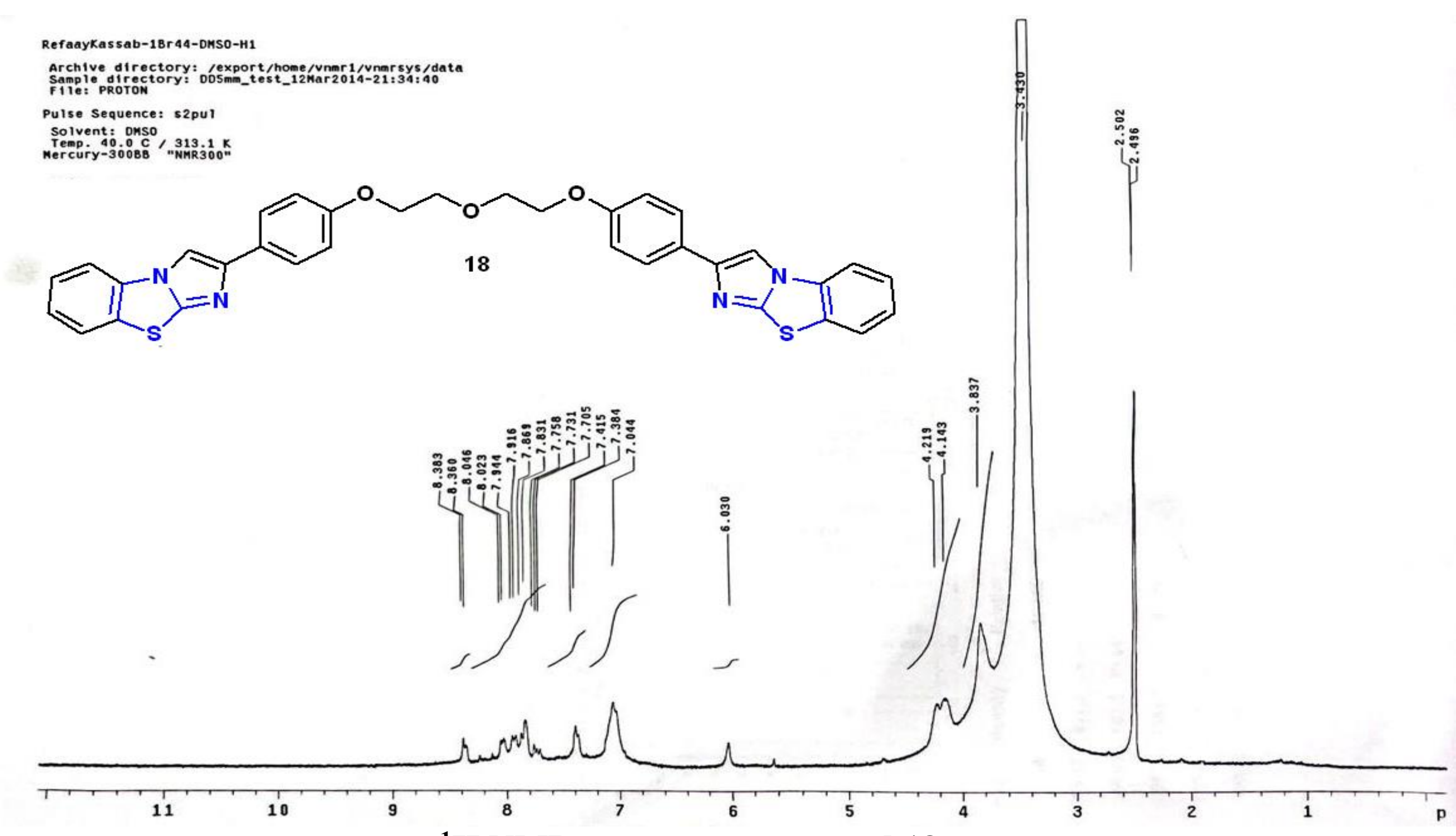

^{1}H NMR spectrum compound 18

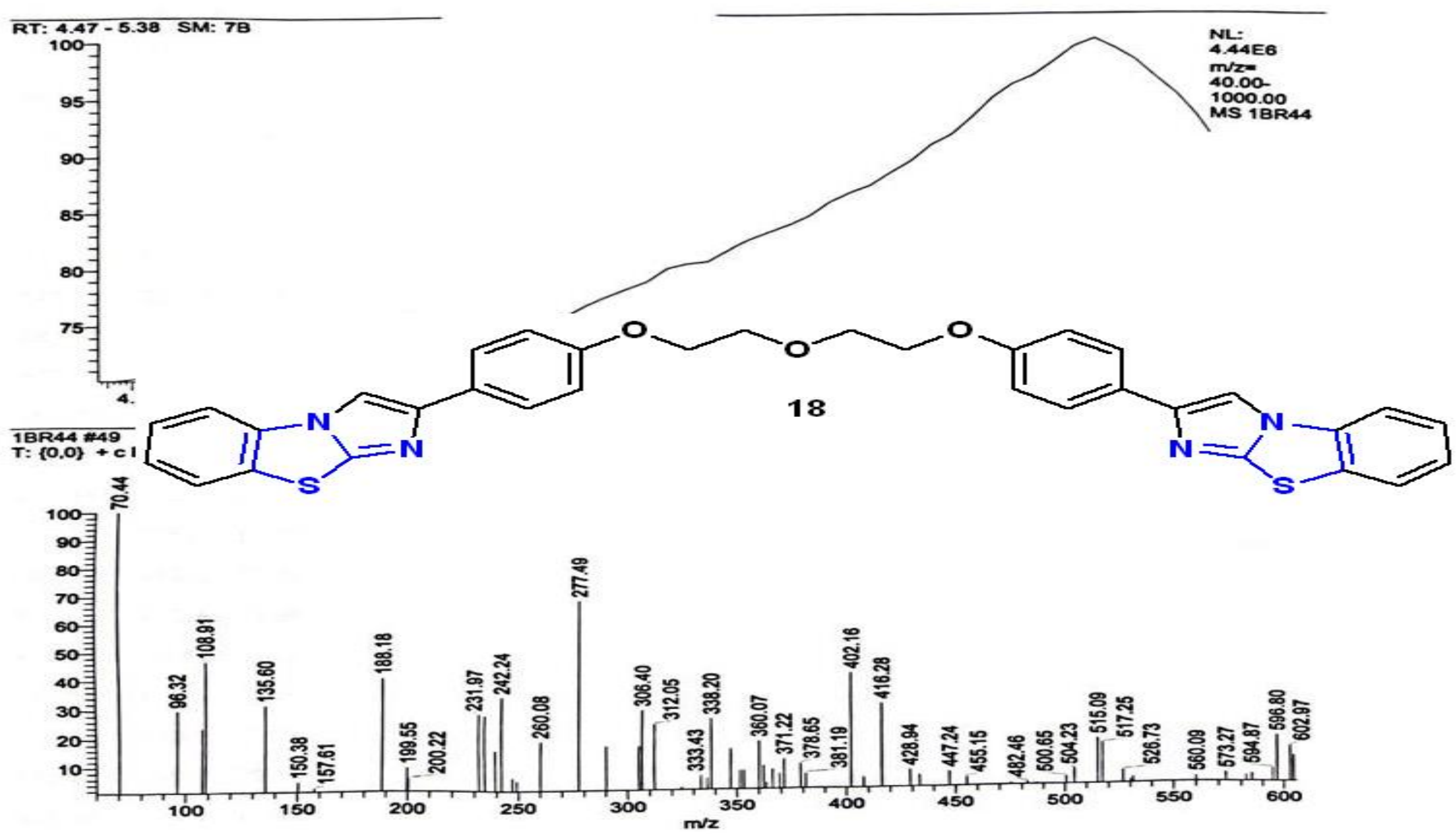

Mass spectrum compound 18

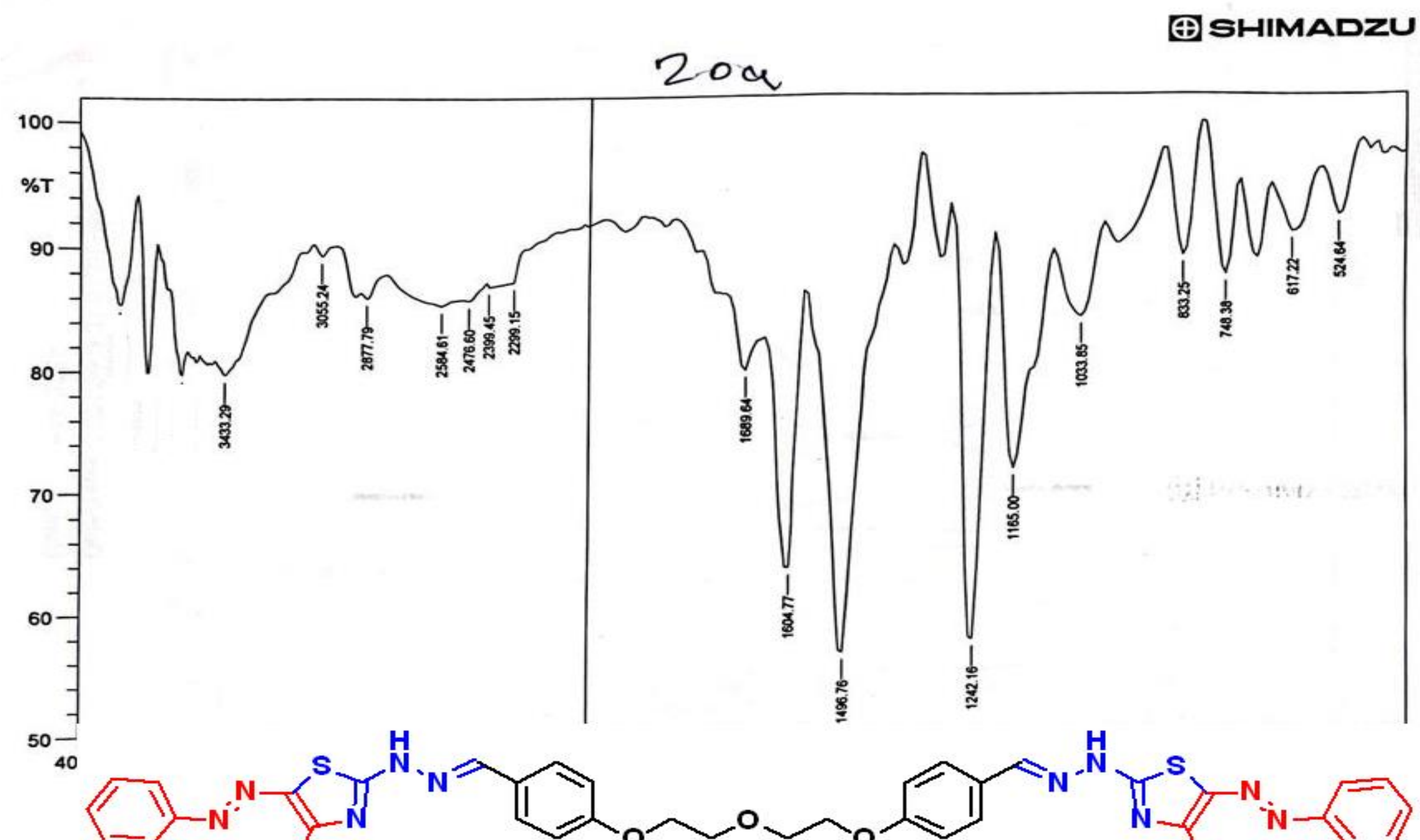

20a

IR spectrum compound 20a

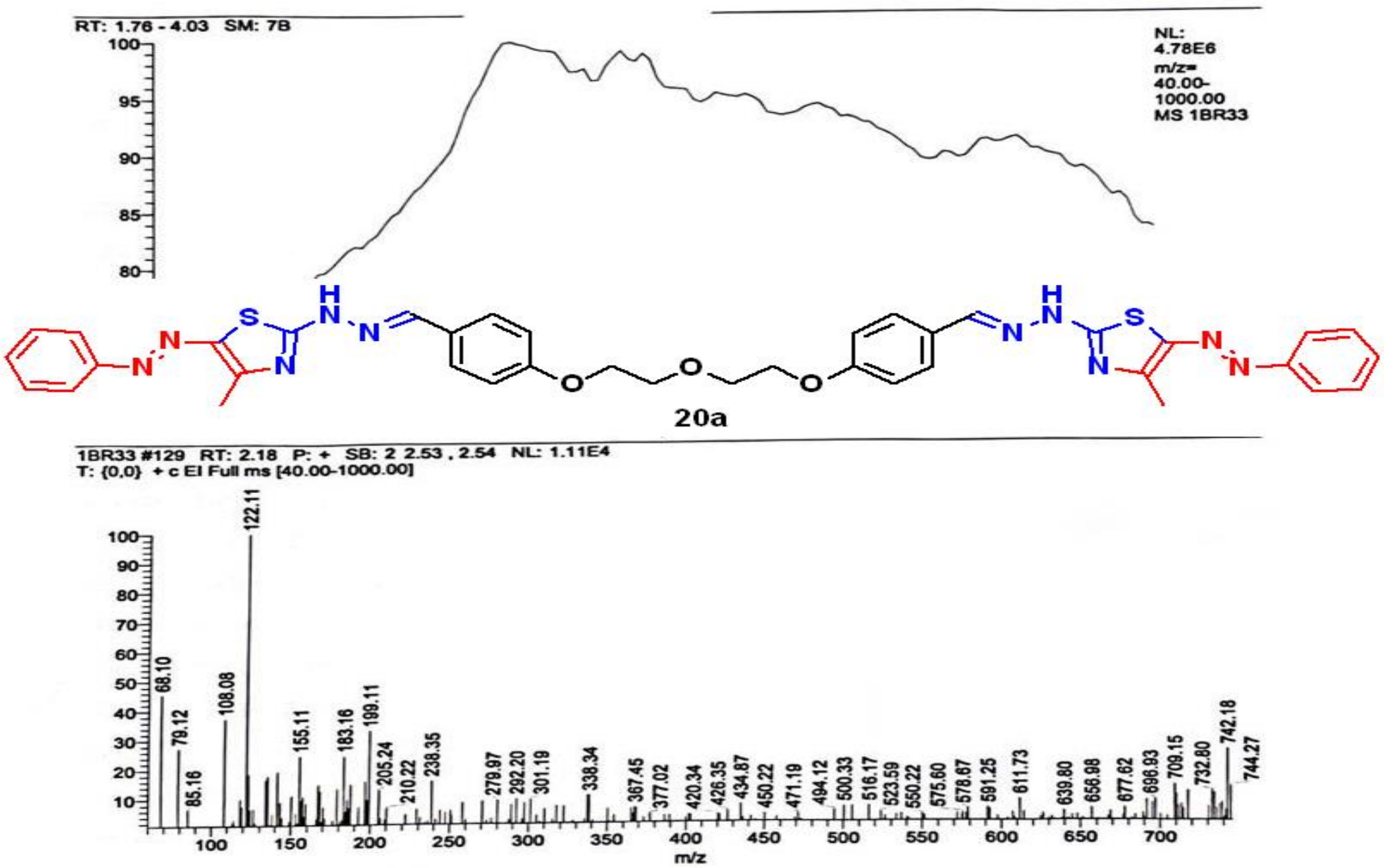

Mass spectrum compound 20a

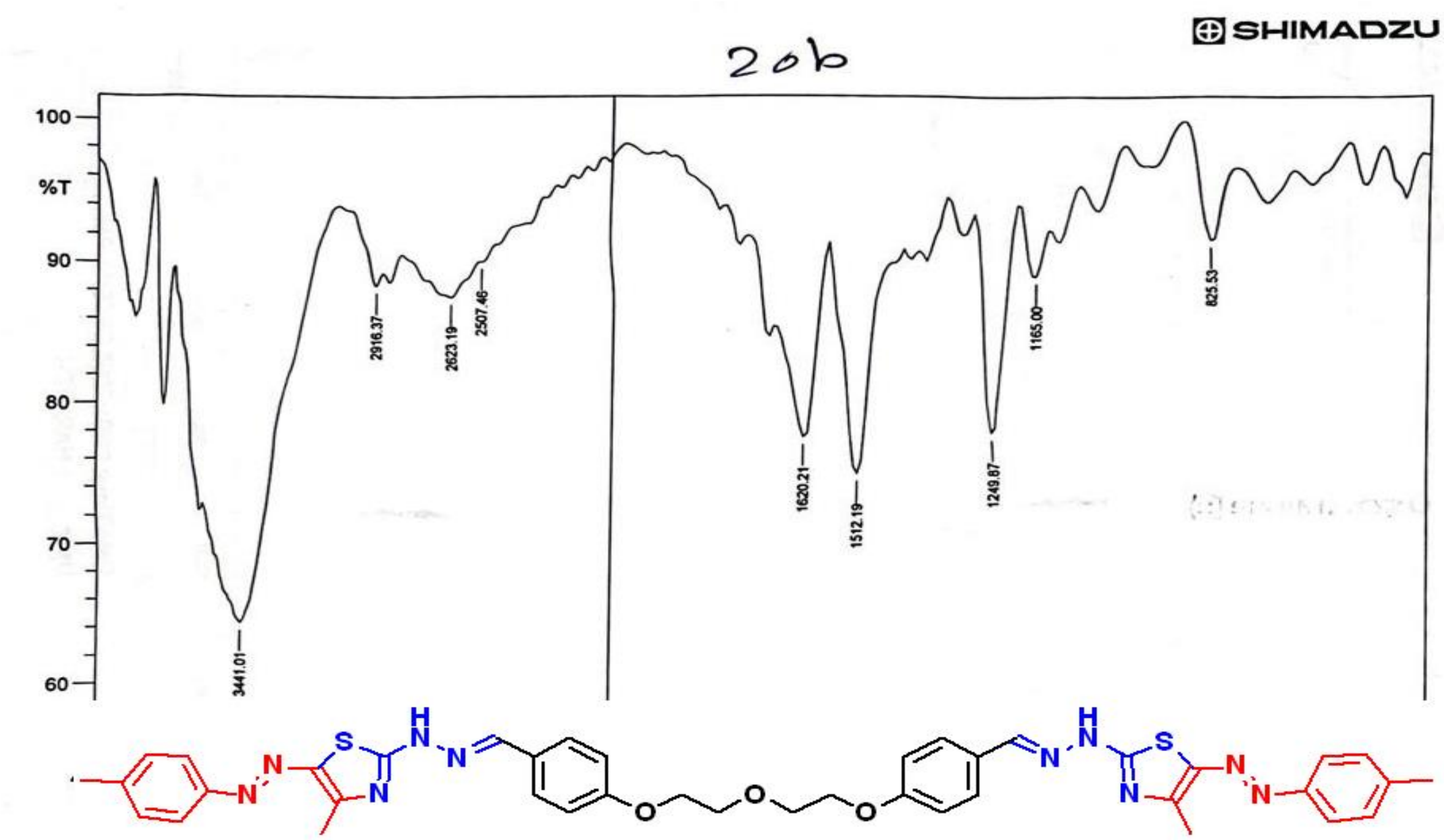

20b

IR spectrum compound 20b

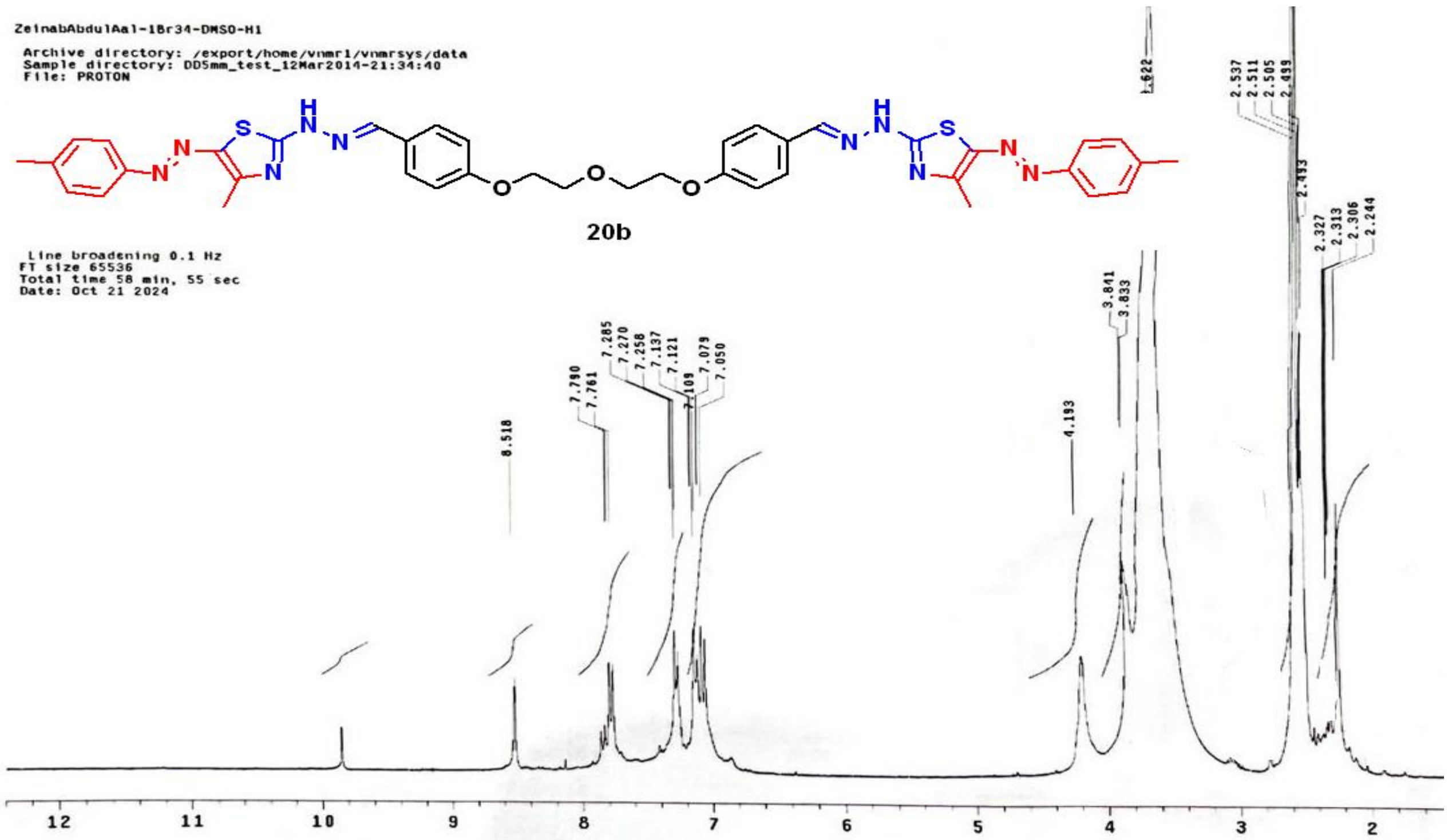

^{1}H NMR spectrum compound 20b

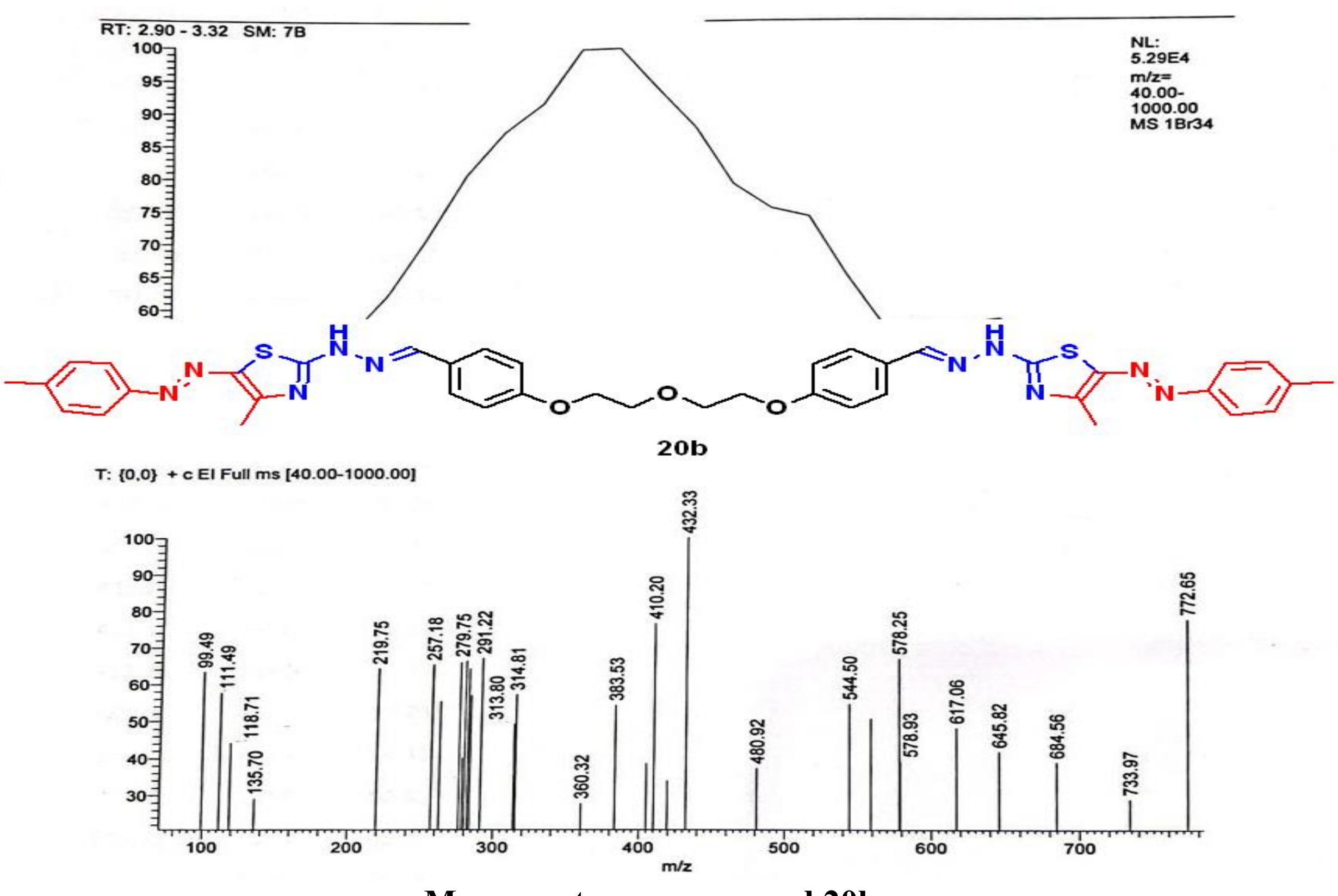

Mass spectrum compound 20b

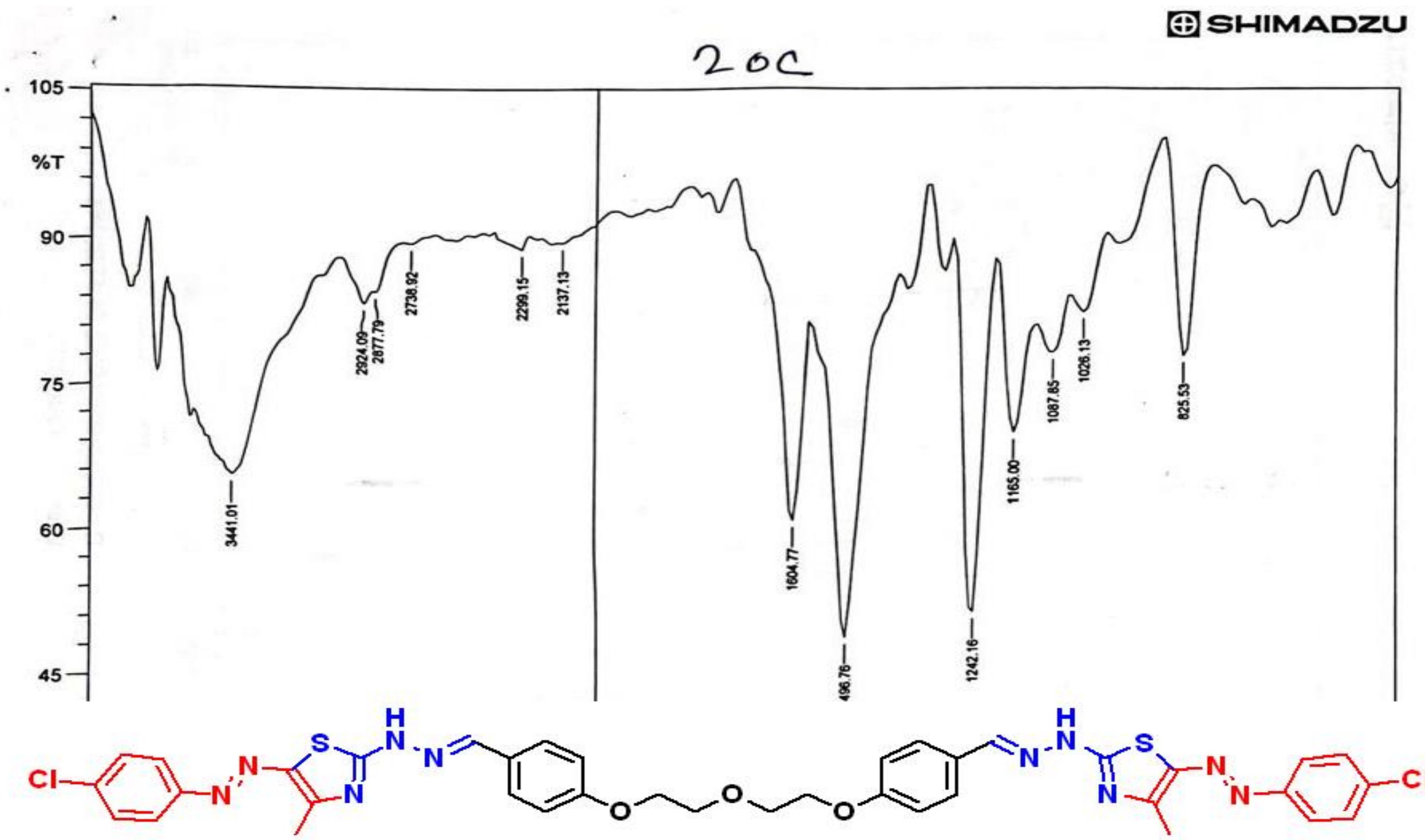

20c

IR spectrum compound 20c

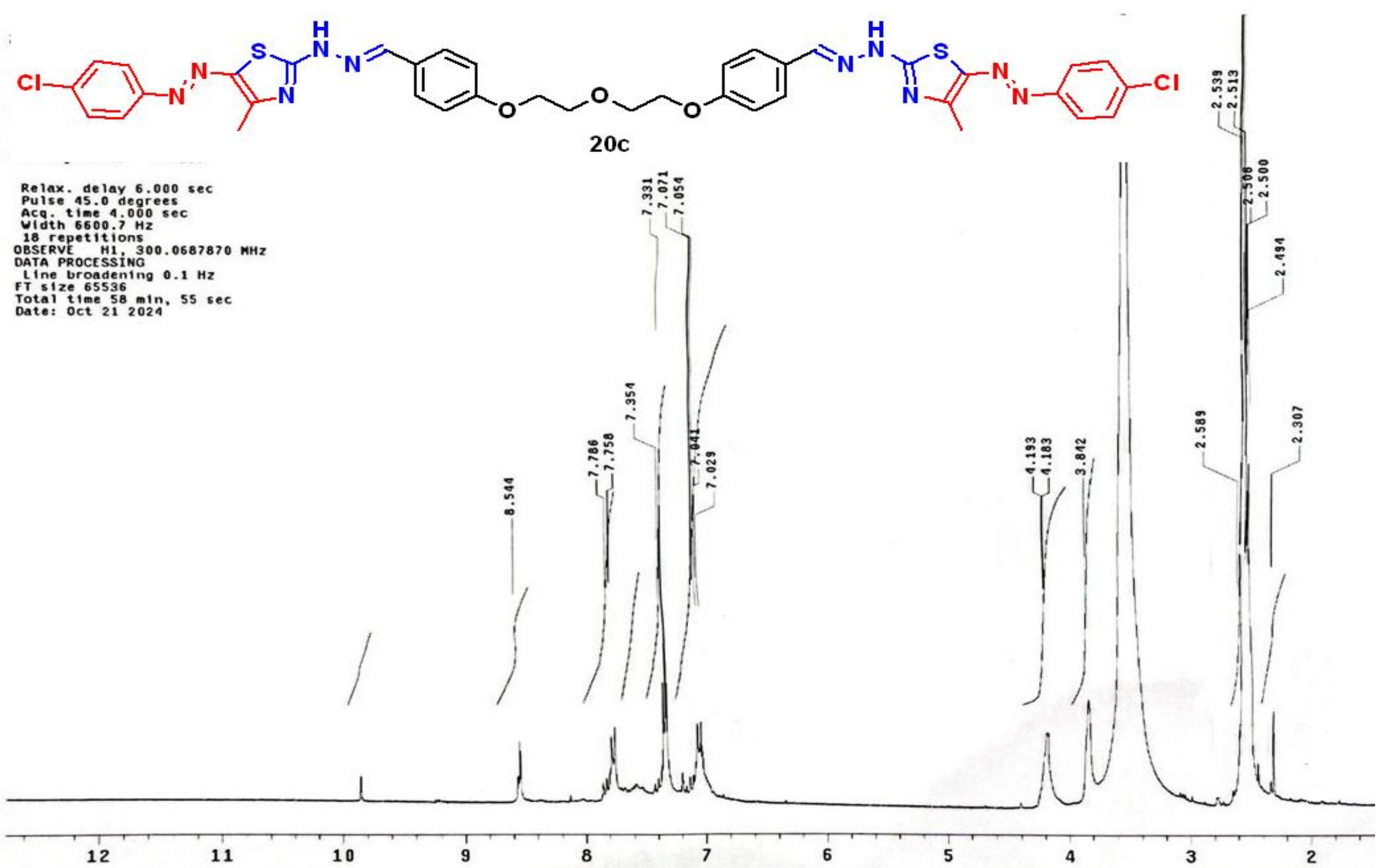

^{1}H NMR spectrum compound 20c

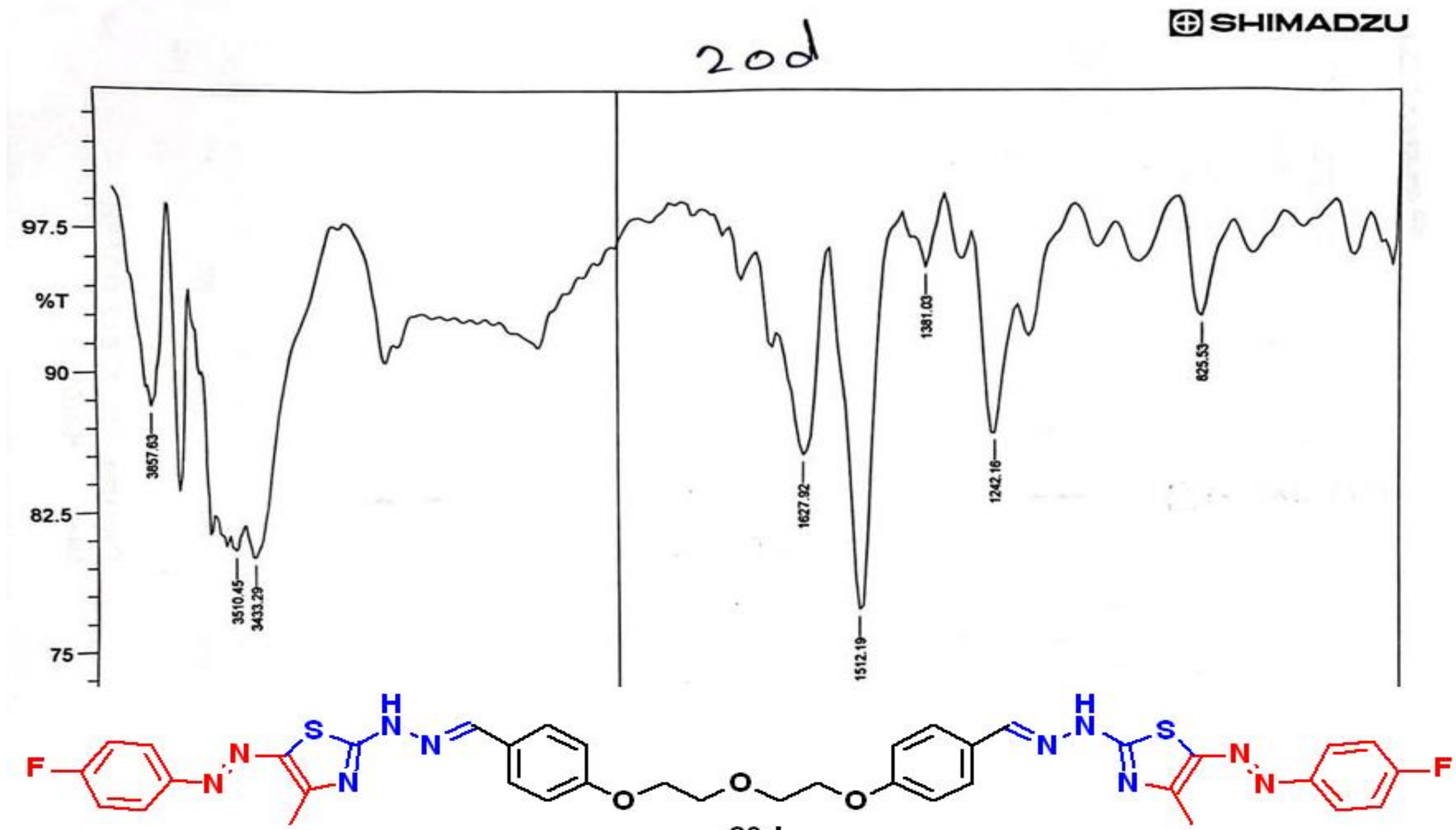

20d

IR spectrum compound 20d

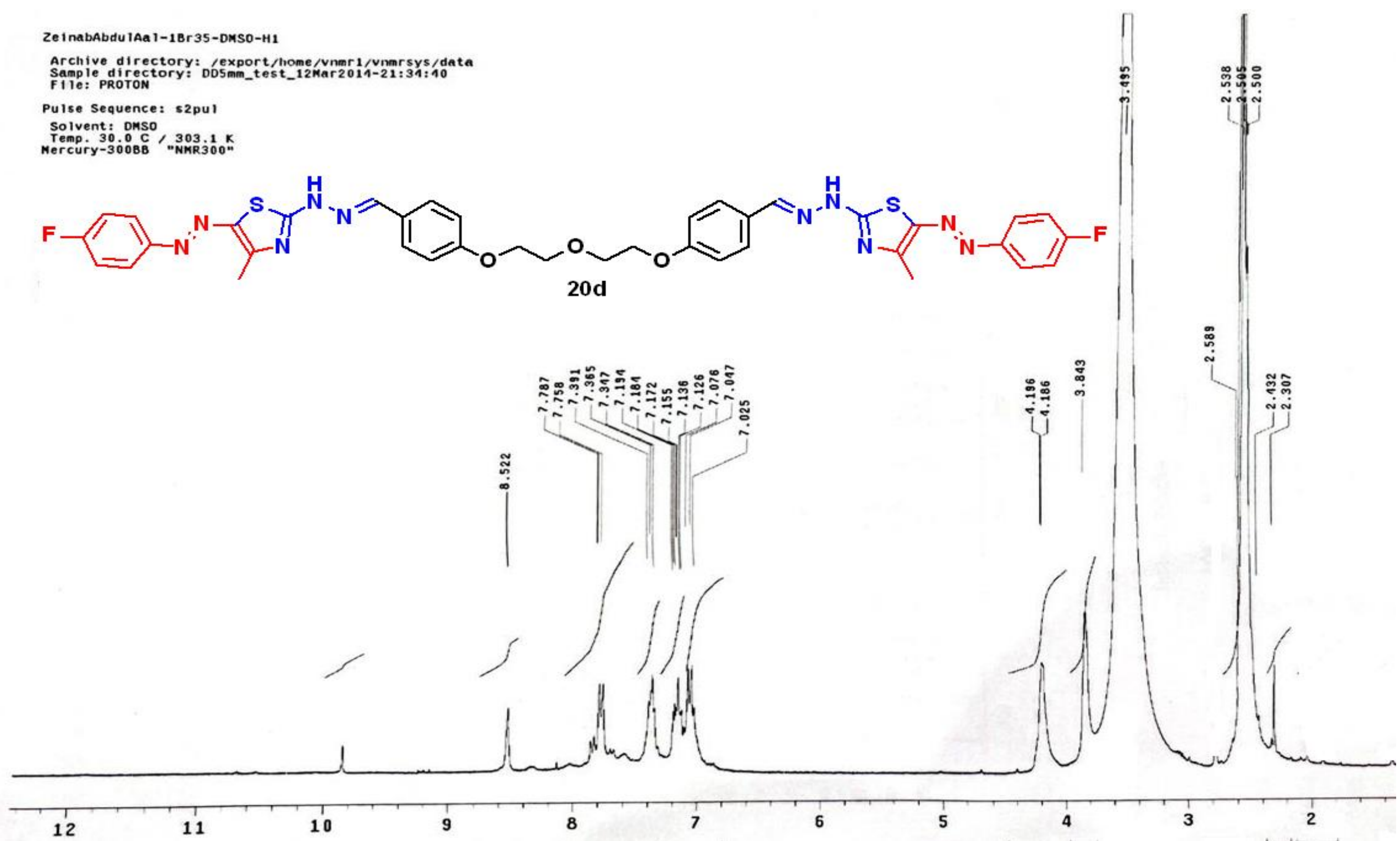

^{1}H NMR spectrum compound 20d

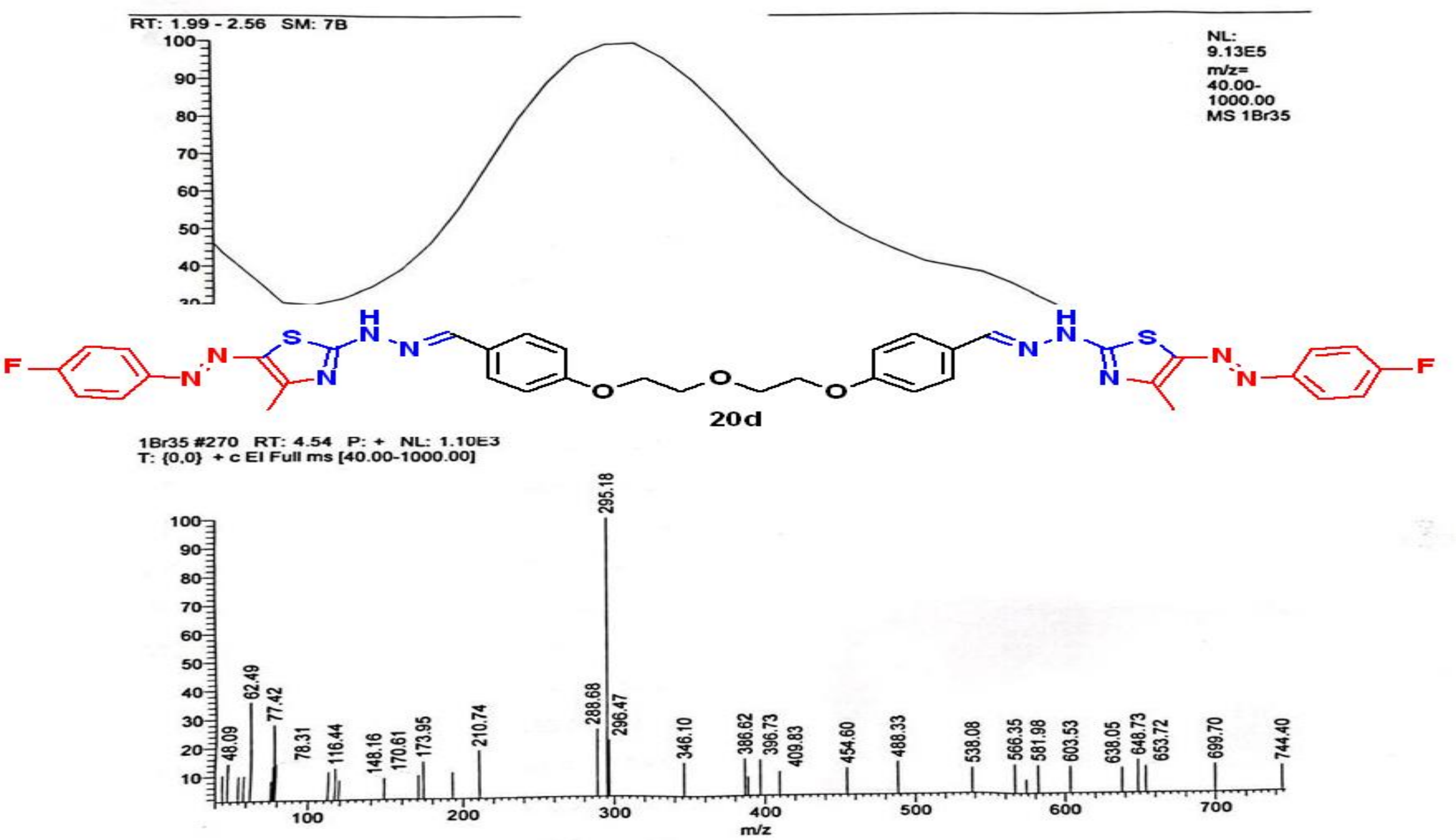

Mass spectrum compound 20d

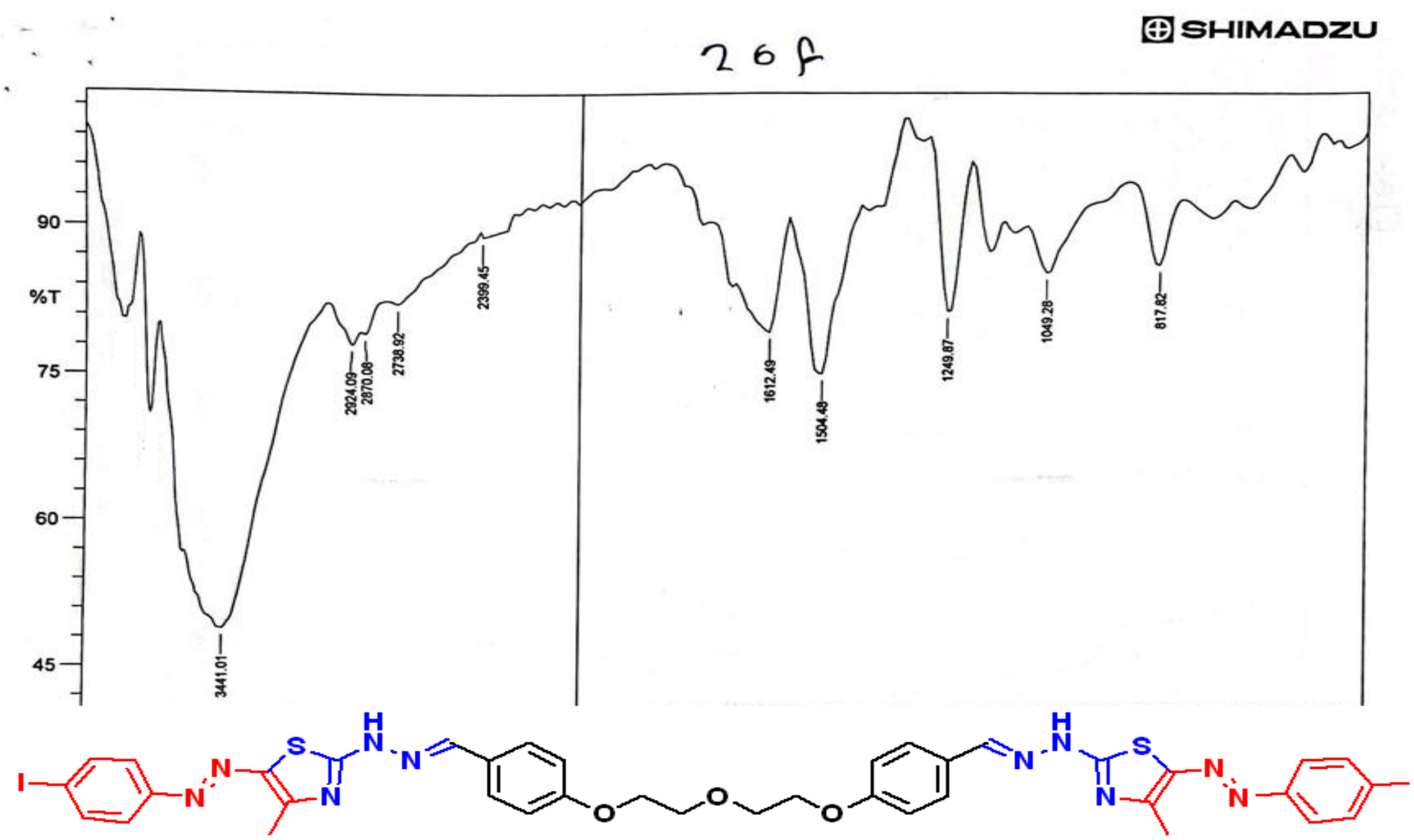

20f

IR spectrum compound 20f

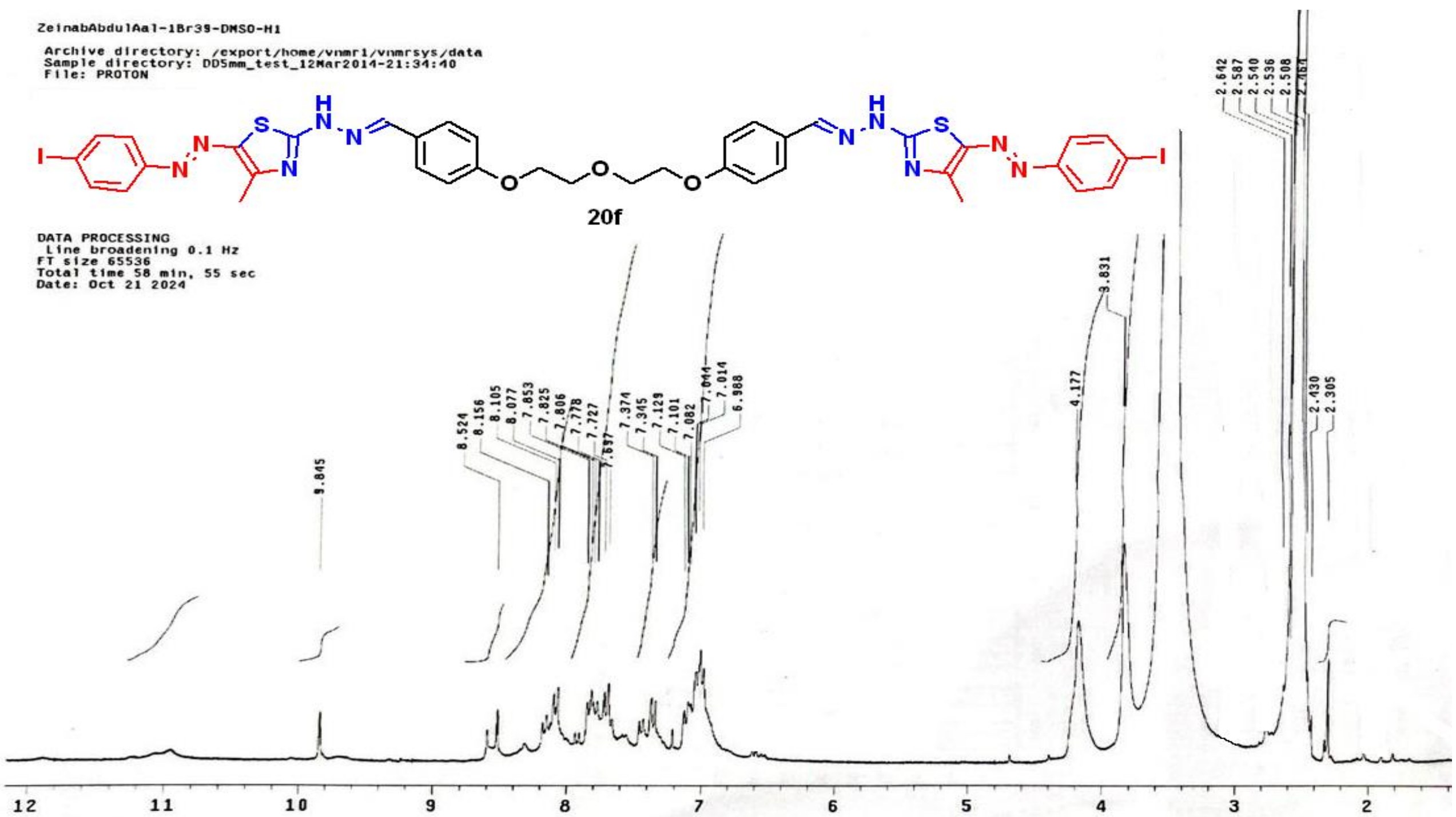

^{1}H NMR spectrum compound 20f

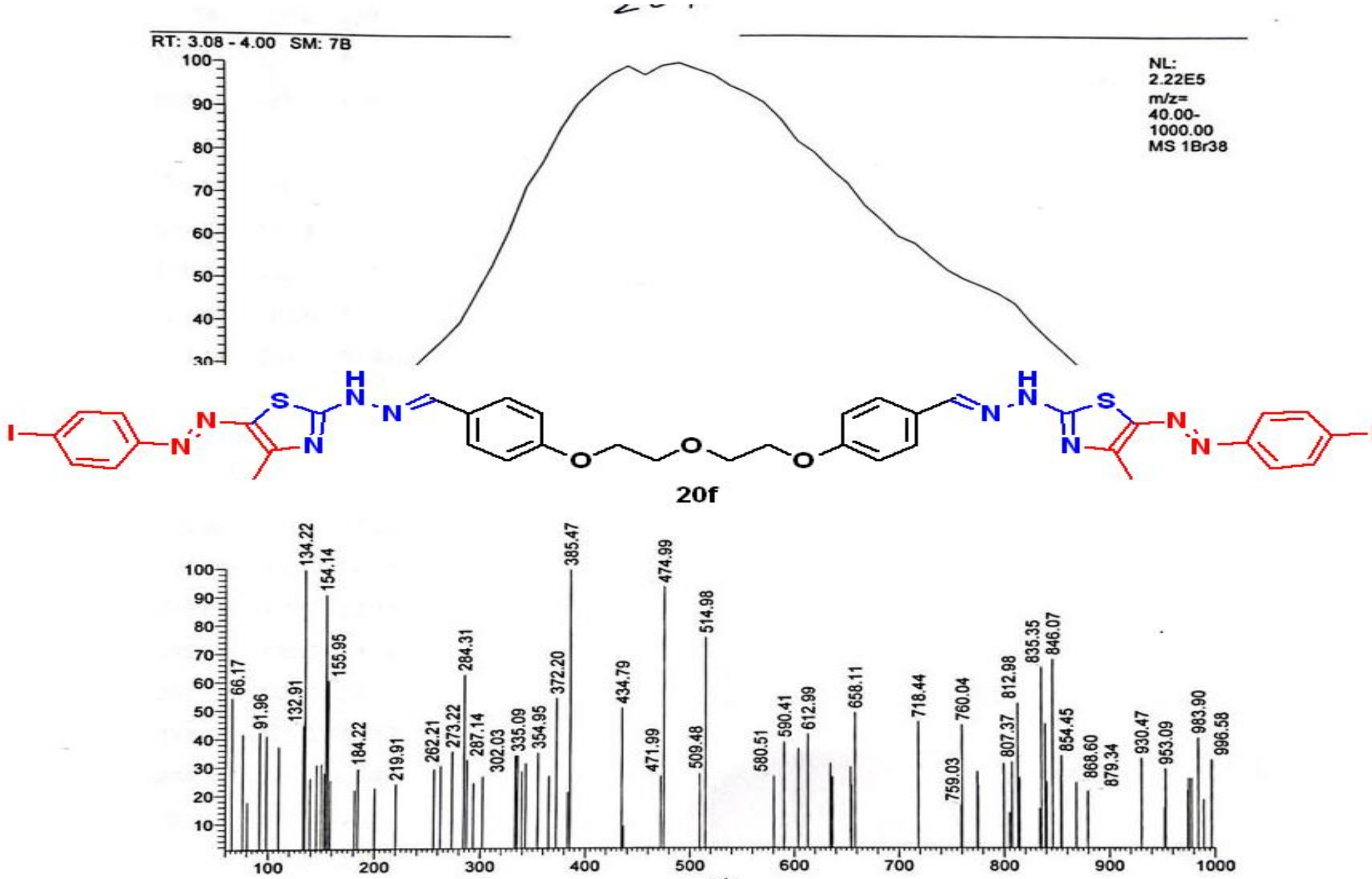

Mass spectrum compound 20f

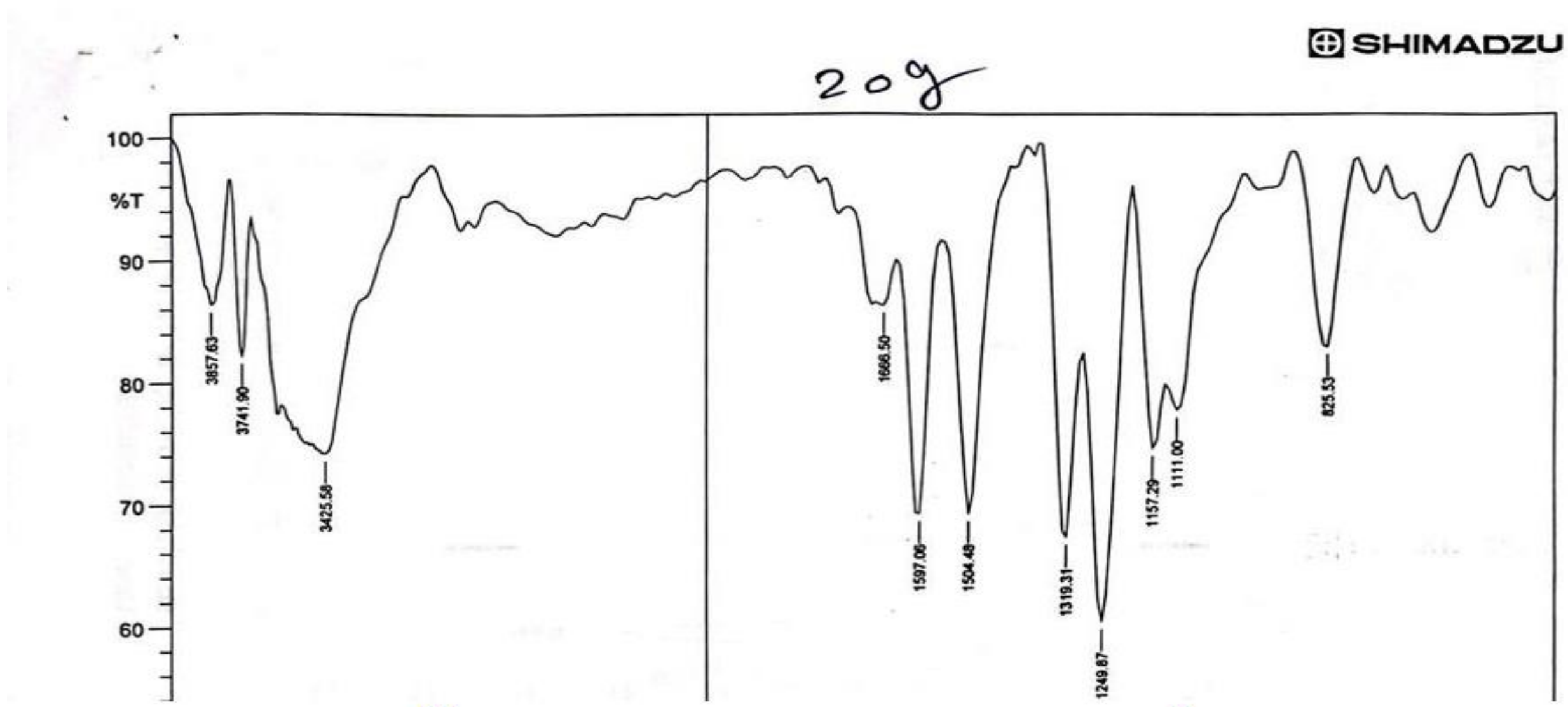

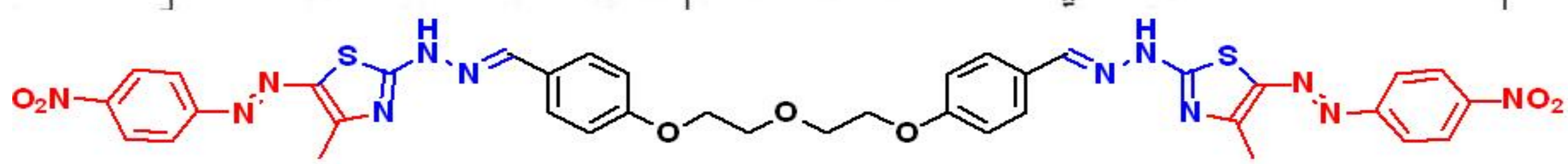

20g

IR spectrum compound 20g

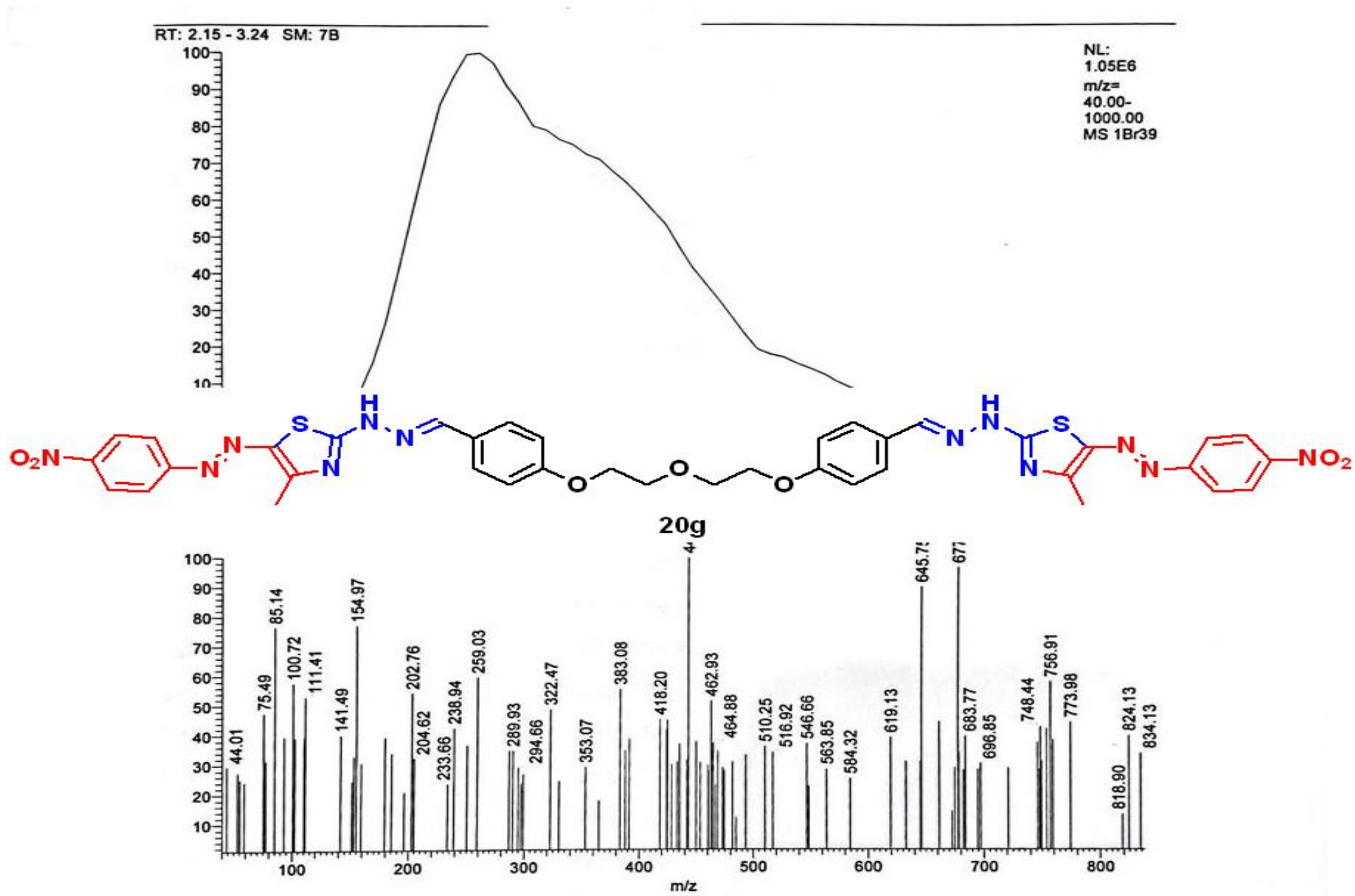

Mass spectrum compound 20g
